# Comprehensive Assessment of Biomolecular Interactions of Morpholine-Based Mixed Ligand Cu(II) and Zn(II) Complexes of 2,2′-Bipyridine as Potential Anticancer and SARS-CoV-2 Agents: A Synergistic Experimental and Structure-Based Virtual Screening

**DOI:** 10.1155/2022/6987806

**Published:** 2022-12-12

**Authors:** Karunganathan Sakthikumar, Rui Werner Maçedo Krause, Bienfait Kabuyaya Isamura

**Affiliations:** ^1^Department of Chemistry, Center for Chemico- and Biomedicinal Research (CCBR), Faculty of Science, Rhodes University, Grahamstown 6140, Eastern Cape, South Africa; ^2^Center for Chemico- and Biomedicinal Research (CCBR), Faculty of Science, Rhodes University, Grahamstown 6140, Eastern Cape, South Africa; ^3^Department of Chemistry, The University of Manchester, Manchester M13 9PL, UK

## Abstract

A new class of pharmacologically active mixed-ligand complexes (**1a**-**2a**) [M^II^(L)_2_ (bpy)], where *L* = 2-(4-morpholinobenzylideneamino)phenol), bpy = 2,2′-bipyridine, M^II^ = Cu (**1a**), and Zn (**2a**), were assigned an octahedral geometry by analytical and spectral measurements. Gel electrophoresis showed that complex (**1a**) demonstrated the complete DNA cleavage mediated by H_2_O_2_. The overall DNA-binding constants observed from UV-vis, fluorometric, hydrodynamic, and electrochemical titrations were in the following sequence: (**1a**) > (**2a**) > (**HL**), which suggests that the complexes might intercalate DNA, a possibility that is further supported by the biothermodynamic characteristics. The binding constant results of BSA by electronic absorption and fluorometric titration demonstrate that complex (**1a**) exhibits the highest binding effectiveness among others, which means that all compounds could interact with BSA through a static approach, additionally supported by FRET measurements. Density FunctionalTheory (DFT) and molecular docking calculations were relied on to unveil the electronic structure, reactivity, and interacting capability of all substances with DNA, BSA, and SARS-CoV-2 main protease (Mpro). These observed binding energies fell within the following ranges: −7.7 to −8.6, −7.2 to −10.2, and −6.7 to −8.2 kcal/mol, respectively. The higher reactivity of the complexes compared to free ligand is supported by the Frontier MolecularOrbital (FMO) theory. The *in vitro* antibacterial, cytotoxic, and radical scavenging characteristics revealed that complex (**1a**) has the best biological efficacy compared to others. This is encouraged because all experimental findings are closely correlated with the theoretical measurements.

## 1. Introduction

Today, cancer has achieved the second highest death rate in the world, behind heart disease, making it one of the deadliest diseases due to the uncontrolled proliferation of cell growth and the capability to spread to essential organs. Current treatment protocols are not always effective, are hurting, and generate chronic disorder for the victims, thus generating the imperative demand for putative medications that are health-friendly and reduce adverse negative impacts [1]. Every year, about 13.2 million people worldwide die of cancer, and an estimated 21.4 million additional cases will occur by 2030, which is also expected to overtake those of communicable diseases in the next decade [2, 3]. Despite ongoing efforts to increase the availability of platinum-based drugs such as cisplatin, carboplatin, lobaplatin, oxaliplatin, heptaplatin, nedaplatin, and miriplatinhydrate for chemotherapeutic treatment in recent decades [4, 5], cancer mortality rates have not yet decreased significantly owing to these medications' limited efficacy, nonselectivity, resistance, and high-risk profiles. Numerous intrinsic limitations arise because of covalent interactions with DNA. These limitations include myelosuppression, thrombocytopenia, neutropenia, nephrotoxicity, ototoxicity, and peripheral neuropathy [6, 7]. This has prompted scientists to generate DNA-targeting anticancer medications that are not reliant on platinum. The majority of noncovalent interactions involve intercalation among base pairs or trapping medicine in the major or minor groove of the deoxyribonucleic acid double helix. These interactions could also be a factor in DNA strand breaks that cause single- or double-strand breaks, which interfere with enzyme function, obstructing fundamental cell functions including replication, transcription, repair, and ultimately resulting in cell death. Consequently, creating safe and powerful anticancer medications with unique modalities of action is extremely intriguing in the realm of bioinorganic chemistry [8]. Transition metal complexes with heterocyclic ligands have received the focus of special attention among chemists across the world during the past four decades because of their structural versatility, ease of formation, and stability under various oxidative and reductive conditions [9]. These have all been essential in the evolution of the coordination chemistry of these complexes with excellent biological activity in many biological systems, as well as their use for a range of pharmacological, analytical, agronomic, commercial, and clinical purposes [10, 11]. Particularly, copper, cobalt, manganese, nickel, and zinc complexes have played an important role in medical imaging, modelling, herbicides, anticancer, antibacterial, antifungal, and other potential biological activities [12, 13].

Recently, a number of mixed ligand complexes possessing O, S, and N-donor binding sites of heterocyclic moieties like morpholine, pyrimidine derivatives incorporated with 2,2′-bipyridine/1,10-phenanthroline coligands have been extensively studied owing to their unusual electromagnetic characteristics, an eccentric structure, and a range of chemical kinetics, which results in their promise for fighting cancer and bacterial strains [14–16]. These ligands may also have an impact on the planarity, hydrophobicity, and coordination structure of the complexes, which could eventually increase their affinity for binding DNA and improve the ability of metal-based medications to treat cancer [17–19]. Generally, the mixed ligand complexes can bind efficiently with biomolecules through hydrophobic, exterior electrostatic, and major/minor groove interactions and cleave the DNA under physiological conditions. It further reveals that the complexes' geometry, size, hydrophobicity, planarity, charge, and ability to form H-bonds with the ligand may influence the binding modes, position, affinity, and even the cleavage action slightly or dramatically [20–22]. Additionally, they are more adaptable to fit into the binding sites of different enzymes and receptors in biological systems and encourage the production of ROS, which ultimately prompts apoptosis or necrosis by inducing DNA damage and mitochondrial dysfunction. Numerous research reports put forward that *N*-functionalized morpholine derivative transition metal complexes show diverse pharmacological activities and are utilized to treat cancer, inflammatory diseases, pain, migraine, asthma, microbial and viral diseases, etc. [23, 24]. Also, the ternary copper(II) mixed ligand complexes that are strongly bound to DNA via non-covalent interactions, have high conformational selectivity, and efficiently oxidatively cleave DNA. Moreover, they have been revealed to have excellent anticancer efficacy compared to cisplatin [25, 26].

Furthermore, the electronic configuration and molecular characteristics of the ground and excited states of biologically active compounds can also be visualized using computational measurements [27]. To realize the binding mechanisms, evaluate the longevity of the guest-host molecules, and clarify the types of interactions that contribute to the stability of the compounds, docking properties are designed to simulate the binding between biomacromolecules and chemical substances. A variety of forces, including ion contacts, sigma-hole interactions, H-bonds, *π*-*π* stacking interactions, and numerous other noncovalent interactions, often support the stability of chemical substances within a protein's active site [28]. Since morpholine scaffold ligands and their heterocyclic derivatives with their mixed ligand transition metal(II) complexes are appealing origin of novel chemical moieties for the creation and discovery of new drugs, our research team has also been engaged in the design and synthesis of much more effective and selective anticancer analogues by chemically altering lead compounds from medicinal plant extractions. The binding affinity of these complexes (**1a**-**2a**) has further been examined in this work utilizing molecular docking studies employing DFT optimized geometry for all substances on DNA/BSA/SARS-CoV-2 proteins. Additionally, our findings encourage additional research targeted at confirming the expected activity and aiding in the fight against existing or upcoming viral pandemics.

## 2. Experimental Section

### 2.1. Materials and Techniques

From the Sigma-Aldrich Company, we acquired the necessary chemicals and other reagents. The ligand (HL) and its mixed ligand complexes (**1a**-**2a**) were measured by a variety of analytical and spectroscopic studies. The complete data sets were summarized in our earlier reports [6, 28].

### 2.2. Assessment of DNA-/BSA-Binding Features

#### 2.2.1. Assessment of DNA Nuclease Efficacy

The characteristics were examined of all substances along with DNA containing H_2_O_2_ and Tris-HCl buffer solution (pH: 7.4) [6, 28]. The tank solution's gel layer was lifted out after the experiment was finished and put in front of a UV transilluminator. Additionally, each band lane was scrutinized with control (DNA + H_2_O_2_) [29, 30].

#### 2.2.2. Analysis of DNA Interaction Characteristics

The DNA-binding experiment was conducted by an electronic absorption spectrophotometer by raising the DNA concentration from zero to 50 *μ*M to the given concentration of all samples (50 *μ*M) in Tris-HCl buffer solution (pH: 7.4) at 25°C [6, 28, 31, 32].

#### 2.2.3. Assessment of Thermal Denaturation Characteristics

Thermal denaturation characteristics were executed by a UV-visible spectrophotometer in a temperature-controlled sample container in both the presence and absence of the substances. In a Tris-HCl buffer solution with a pH of 7.4, all substances were incubated with CT-DNA in a 1 : 1 ratio. Temperature increases of two degrees Celsius per minute were applied to test substances in the range of 25 to 100°C, and the changes in absorbance at 260 nm were carefully scrutinized [33–35].

#### 2.2.4. Assessment of DNA Affinity by Hydrodynamic Technique

The titrations were performed for all compounds including EB (control) at 20, 40, 60, 80, and 100 *μ*M concentrations. In the Ostwald viscometer, these substances were also separately treated with the calf thymus deoxyribonucleic acid solution (100 *μ*M) [6, 28, 36].

#### 2.2.5. Assessment of DNA-/BSA-Binding Characteristics by Fluorometric Approach

The experiment was performed in the 200 to 800 nm region. This is supporting evidence for the complexes' manner of deoxyribonucleic acid binding. When DNA (200 *μ*M) was present or absent, we carefully monitored the intensity variations between 610 nm and 510 nm during the initial emission and excitation of EB [6, 28, 37]. Similarly, emission titrations for BSA interaction with different concentrations between 2.5 *μ*M and 25 *μ*M for all substances were conducted in a Tris-HCl buffer solution (pH: 7.4) between the regions of 300 nm and 400 nm [38].

#### 2.2.6. Förster's Theory-Based FRET Computation

As per Förster's theory, the critical distance of donor-acceptor molecule system can be estimated using FRET to assess the binding affinity between BSA and substance systems [39–41].

#### 2.2.7. Analysis of DNA-Binding Characteristics Using the CV Method

The CV titration for free substances was conducted at 10 *μ*M at 25°C in the presence of Tris-HCl buffer solution (pH: 7.4). While CT-DNA increases (0–10 *μ*M) in each sample solution, a shift in potential, including variations in the peak currents of anodic and cathodic sides, has been monitored [6, 28, 42].

#### 2.2.8. Assessment of BSA-Binding Characteristics by Absorption Titration

In the presence of Tris-HCl buffer solution, the *UV*-*vis* absorption titrations were done with a 25 *μ*M concentration of BSA at room temperature. While the sample concentrations (0–25 *μ*M) increased in the same BSA concentration solution, the change in the absorption band at 278 nm was continuously noted [6, 28, 43].

### 2.3. DFT and Molecular Modelling Properties

All compounds were fully optimized with the help of the hybrid B3LYP functional as accomplished in the Gaussian 09 package [44]. To demonstrate the global and local reactivity of all substances, the FMO hypothesis [45] and electrostatic potentials within molecules [46] were studied. Using the B3LYP-optimized structures of each substance, docking computations were also carried out. The Autodock Vina software was used for input structure preparation and calculations [47], and the visualization was performed by Discovery Studio [48].

### 2.4. UV-Vis Absorption Titration for In Vitro Antioxidant Assay

All samples were evaluated for their scavenging ability with the help of UV-visible spectrophotometer at different concentrations of 40, 80, 120, 160, 200, and 240 *μ*M [6, 28]. While performing the antioxidant properties for the DPPH, hydroxyl, superoxide, and nitric oxide radical scavenging, the absorbance at 517, 230, 590, and 546 nm, respectively, was closely observed. In addition, the observed IC_50_ values of all samples were compared with ascorbic acid [49–51].

### 2.5. Assessment of in Vitro Antimicrobial Properties

In-vitro antimicrobial properties were evaluated for all samples by the agar disc diffusion method towards different fungal and bacterial strains [6, 28, 52–54]. Additionally, the reported inhibition zone values were comparatively analyzed with the standard antifungal drugs *ketoconazole* and *amphotericin B* as well as the standard antibiotic medications *streptomycin* and *amikacin*.

### 2.6. MTT Cell Viability Assay for Anticancer Characteristics

All substances towards the A549, HepG2, MCF-7, and NHDF cell lines were evaluated by using the MTT approach [6, 28]. The collected data were utilized to compute the IC_50_ value and contrast it with the gold standard cisplatin anticancer medication [55].

## 3. Results and Discussions

It is observed that all compounds are highly pigmented and faintly hygroscopic and have high solubility in CH_3_OH, C_2_H_5_OH, CHCl_3_, and DMSO. The evaluated analytical results and structural characteristics are presented in the electronic supplementary information file (**3a**) (Figures S1–S14 and Tables S1–S13).

### 3.1. Synthetic Process and Properties

The evaluated analytical results, structural characteristics, as well as crystallographic data for ligand (**HL**) and its mixed ligand complexes (**1a**-**2a**) (Scheme 1) are presented in the electronic supplementary information file (**3a**) (Tables S1–S13 and Figures S1–S14).

### 3.2. DNA/BSA-Binding Properties

In general, it is recommended to restrict the growth of tumor cells by preventing the reproduction of DNA that has been damaged or broken due to binding or cleavage mechanisms. It deals with the static mode of binding between test compounds and BSA.

#### 3.2.1. Analysis of DNA Cleavage Characteristics

**Figure 1:**
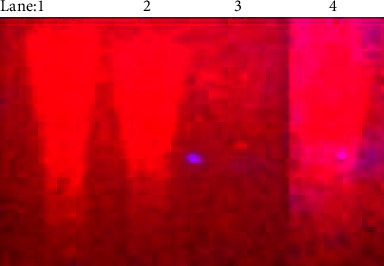
Ethidium bromide displacement assay: gel electrophoresis demonstrates the DNA cleavage property in the H_2_O_2_ environment for the following substances: Lane: 1 DNA alone + H_2_O_2_; lane: 2 ligand (**HL**) + DNA + H_2_O_2_; lane: 3 complex (**1a**) + DNA + H_2_O_2_; lane: 4 complex (**2a**) + DNA + H_2_O_2_. Raw data for electrophoretic gels and blots were also enclosed in the electronic supplementary information file.

Using the gel electrophoresis method, the DNA nuclease properties for all samples were assessed under the H_2_O_2_ environment. The examined DNA nuclease efficacy for all complexes (**1a**-**2a**) was contrasted with free ligand (**HL**) and CT-DNA alone. The raw data for electrophoretic gels and blots were also enclosed in the electronic supplementary file (Figure S15). In Figure 1, no substantial nuclease activity can be seen in the control (lane 1; DNA + H_2_O_2_) even after a lot of time has passed and the free ligand (HL) (lane: 2) was monitored as immobile in an H_2_O_2_ environment. Lane: 3 demonstrates that the mixed ligand complex (**1a**) demonstrates complete DNA cleavage. Similarly, lane: 4 reveals that complex (**2a**) partially cleaves DNA. In addition, the performance of the band reduction in the lanes was revealed in the agarose gel (Figure 1). Furthermore, ROS include O_2_^•−^, H_2_O_2_, OH^•^, ROOH, ROO^•^, HOCl, and ^1^O_2_ (singlet oxygen) and ozone (O_3_), which play essential roles in living systems. Consequently, it has commonly been acknowledged that ROS plays a dual physiological role in controlling a variety of illnesses as well as cellular homeostasis (self-regulating processes such as thermoregulation, blood glucose regulation, calcium/potassium homeostasis, and osmoregulation) [56]. Numerous oxidases, peroxidases, lipoxygenases, dehydrogenases, cytochrome P450, and other enzymes have been demonstrated to be able to produce ROS. Additionally, it is widely known that NADPH oxidase generates reactive oxygen species as a portion of its antibacterial effect on phagocytic cells. Nevertheless, these types of enzymes seem to be present in a variety of other cells as well and may have significant signalling pathway functions. When noncarcinogenic toxicity events occur, ROS has the ability to alter cell function as well as affect the genes of cancer at several levels. OH^•^ can attack DNA, proteins, and lipids due to its high reactivity among ROS. The hydroxyl radical is a key participant in free radical-mediated hazardous reactions because of its great reactivity. The free radicals are also essential for the redox regulation of many cellular signalling pathways and proper cellular functions. Superoxide (O_2_^•−^) was believed to be a typical cellular metabolite. It was then realized that more dangerous radicals may potentially be produced *via* the Haber–Weiss process. The combination of O_2_^•−^ and H_2_O_2_ may produce a powerfully reactive OH^•^ radical [57]. Moreover, as per the Fenton/Haber–Weiss mechanism, it is suggested that it is capable of vigorous nucleolytic cleavage of chemical substances in an oxidizing agent (H_2_O_2_) environment [58]. According to this mechanism, the complexes acted as excellent vehicles for the creation of diffusible ^•^OH free radicals from hydrogen peroxide. Additionally, ^•^OH free radicals abstract the H-atom from the sugar fragment of the deoxyribonucleic acid base pair to generate sugar radicals. Concerning the location of the hydrogen atom, it rapidly induces the hydrolytic nuclease activity at the sugar-phosphate backbone [59]. The rapid migration of deoxyribonucleic acid can lead to the open circular form's transformation into a linear form. Meanwhile, EDTA facilitates the generation of highly reactive diffusible ^•^OH and anions *via* the Fenton or Haber–Weiss processes and further prevents metal ions from binding with DNA *via* intercalation due to the generation of an EDTA-metal system. The diffusible hydroxyl free radicals also stimulate the abstraction of the H-atom from the sugar part of the deoxyribonucleic acid base pair to generate sugar radicals along with the formation of an adduct with nucleobases. Therefore, DNA cleavage occurs owing to the assault of a diffusible ^•^OH on deoxyribonucleic acid base pairs in the metal complex environment. The complex serves as an effective catalyst to produce ^•^OH from hydrogen peroxide according to the Fenton mechanism [60]. If metal complexes have strong hydrogen abstraction ability, they exhibit more DNA cleavage properties. Conversely, if metal complexes have weak hydrogen abstraction ability, they reveal less DNA cleavage properties (Figure S16).

#### 3.2.2. Assessment of DNA-Binding Characteristics Using a UV-Visible Spectrophotometer

**Figure 2:**
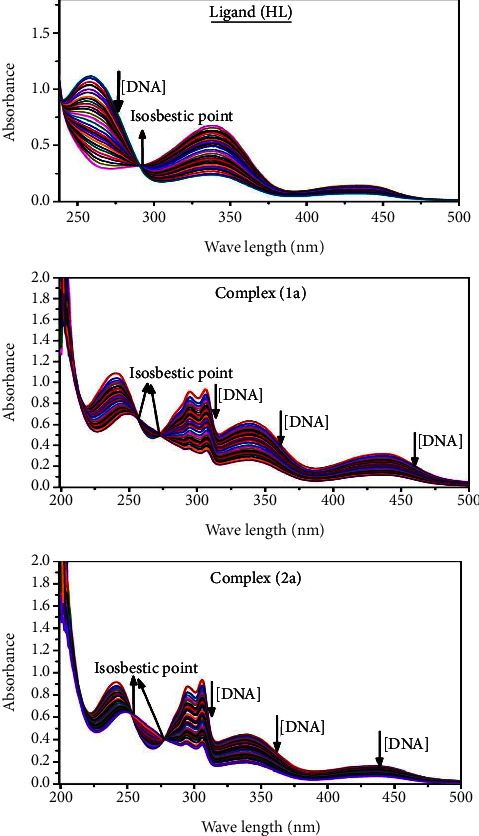
Increasing concentrations of CT-DNA were present while the ligand (**HL**) and its complexes (**1a**-**2a**) were measured for their absorption spectra in a Tris-HCl buffer solution at 25°C. Arrows depict the changes in absorbance that occur as CT-DNA concentration is increased, and another arrow with isosbestic points denotes that equilibrium between DNA and complexes has been achieved.

UV-visible absorption titration is the main imperative approach to observing the tendency of test substances to bind with deoxyribonucleic acid. The approach is commonly utilized to evaluate the potency and mode of binding of the test substances with deoxyribonucleic acid. The essential information regarding conformational change, the effectiveness of the DNA-substance binding, and the negatively charged phosphate on deoxyribonucleic acid are neutralized *via* exterior contact, and intercalation through interactions between *π*-*π* stacks is presented by DNA binding towards metal complexes. All complexes (**1a**-**2a**), including the free ligand (**HL**), were measured in the presence and absence of deoxyribonucleic acid by ultraviolet-visible spectrophotometric titrations under Tris-HCl buffer solution (pH: 7.4) at room temperature (Figure 2). The results are also included in Table 1. In this case, all samples were exposed to two prominent electronic absorption bands at 260 nm and 336–343 nm, consequent to the *π*-*π*^*∗*^ transition of the phenyl chromophore and MLCT, respectively. While the amount of DNA concentration rises in each test compound, the interaction of the chemical substance with DNA base pairs generates noticeable alterations in the intraligand charge transfer bands' strength and wavelength. The hypochromic shift of all compounds was observed in the range of 37.13–52.18% with 4–7 nm red shifts, which occurred due to a diminution in the *π*-*π*^*∗*^ transition energy and the half-packed electrons of bonding orbitals. In contrast, it would be possible for electrostatic interaction if the complex-DNA adduct exhibits hyperchromism with a hypsochromic shift [61, 62]. The *K*_*b*_ findings were measured for all samples by the Wolfe–Shimmer equations (2) and (3) by methods I and II, respectively, from the findings of the slope divided by the intercept in the linear regression plots of [DNA]/(*ɛ*_*a*_ − *ɛ*_*f*_) vs. [DNA] and the intercept divided by intercept slope in the linear regression plot of (*ɛ*_*b*_ − *ɛ*_*f*_)/(*ɛ*_*a*_ − *ɛ*_*f*_) vs. 1/[DNA] M^−1^ (Figure S17), and the *K*_*b*_ results for each sample were in the subsequent sequence: (**1a**) > (**2a**) > (**HL**). Moreover, the observed overall ∆*G*_*b*_° values in all cases were found in the range of –21.55 to –26.04 kJ·mol^−1^ (Table 1), which also indicates that the compounds spontaneously intercalate to DNA. However, complex (**1a**) exhibited excellent binding potency compared to others. It is concluded that the morpholine linked ligand's coplanarity and 2,2′-bipyridine aromatic system complexation with the metal center, which promotes the complex to infiltrate DNA base pairs smoothly, and large aromatic systems may also assist the complex to penetrate the phosphate backbone's core deeply, and those substances may permit the complex to freely penetrate deep into the deoxyribonucleic acid double helix. In addition, the observed isosbestic points are found at 256 and 276 nm for free ligand and complexes (**1a**-**2a**), respectively. It also suggests that DNA and complexes establish a dynamic equilibrium and further conclude that complexes (**1a**-**2a**) spontaneously intercalate into DNA. The Wolfe–Shimmer (1 and 2) [63], Benesi–Hildebrand (3 and 4) [64], and Sakthi–Krause equations (5 and 6) were supported to evaluate *K*_*b*_ results for all samples (Table 1). The Benesi–Hildebrand binding constant (*K*_*b*_) values were measured using equations (3) and (4) by methods I and II, respectively, from the intercept divided by slope in the linear regression plot of [1/(*A*_*x*_ − *A*_0_)] *vs*. {1/[DNA]} M^−1^ and the intercept divided by slope in the linear regression plot of [(*A*_max_ − *A*_0_)/(*A*_*x*_ − *A*_0_)] *vs*. {1/[DNA]} M^−1^ (Figure S18). The *K*_*b*_ values were estimated using Sakthi–Krause equations (5) and (6) by methods I and II, respectively, with the intercept divided by slope in the linear regression plot of [(*A*/(*A*_0_ − *A*)] *vs*. {1/[DNA]} M^−1^ (Figure S19) and the antilogarithm value of the intercept in the linear regression plot of log {1/[DNA]} *vs*. log [(A/(A_0_ − A)]M^−1^ (Figure S20). In addition, the Van't Hoff equation (7) was supported to obtain the ∆*G*_*b*_° values for DNA interaction, and equation (8) was supported to measure the % of chromicity for all substances (Table 1). The overall measured K_b_ findings were in the subsequent sequence: (**1a**) > (**2a**) > (**HL**). In these cases, the observed ∆*G*_*b*_° values were reported in the range of −19.91 to −29.13, −21.55 to −26.04 and −22.50 to –25.22 kJ·mol^−1^, respectively (Table 1). In these cases, the complex (**1a**) had the highest DNA-binding efficacy among all binding results. The DNA cleavage, emission, hydrodynamic, and CV measurements all support the preceding observation.

Hypochromism % *H* = (*ɛ*_*b*_ − *ɛ*_*f*_)/*ɛ*_*f*_ × 100; *ɛ*_*f*_ and *ɛ*_*b*_ are denoted as extinction coefficient of substance alone and extinction coefficient of the completely interacted with deoxyribonucleic acid, WS is represented as Wolfe-shimmer, BH is denoted as Benesi–Hildebrand methods (BH-I and II), SK represented as Sakthi–Krause methods (SK-I and II), ΔG_b_° = –RT, In *K*_*b*_, *K*_*b*_ = Intrinsic DNA-binding constant evaluated from the electronic absorption spectral titration, *R* is a universal gas constant = 1.987 cal·K^−1^·mol^−1^ (or) 8.314 J·K^−1^·mol^−1^, *T* = 298 K, Error limit ± 2.5% (*P* < 0.025).

(1) $$\begin{array}{c} \frac{\left[DNA\right]}{\left({ɛ}_{a}-{ɛ}_{f}\right)}=\frac{\left[DNA\right]}{\left({ɛ}_{b}-{ɛ}_{f}\right)}+\frac{1}{1/K_{b}\left({ɛ}_{b}-{ɛ}_{f}\right)}\text{,} \end{array}$$

(2) $$\begin{array}{c} \frac{\left({ɛ}_{b}-{ɛ}_{f}\right)}{\left({ɛ}_{a}-{ɛ}_{f}\right)}=\frac{1}{K_{b}\left[DNA\right]}+1\text{,} \end{array}$$

(3) $$\begin{array}{c} \frac{1}{\left(A_{X}-A_{0}\right)}=\frac{1}{\left(A_{\max}-A_{0}\right)K_{b}\left[DNA\right]}+\frac{1}{\left(A_{\max}-A_{0}\right)}\text{,} \end{array}$$

where

(4) $$\begin{array}{c} \begin{array}{cc} \frac{\left({ɛ}_{b}-{ɛ}_{f}\right)}{\left({ɛ}_{a}-{ɛ}_{f}\right)}=\frac{\left(A_{\max}-A_{0}\right)}{\left(A_{X}-A_{0}\right)} & \frac{\left(A_{\max}-A_{0}\right)}{\left(A_{X}-A_{0}\right)} \end{array}=\frac{1}{K_{b}\left[DNA\right]}+1\text{,} \end{array}$$

(5) $$\begin{array}{c} \frac{A}{\left(A_{0}-A\right)}=\frac{1}{K_{b}\left[DNA\right]}+1\text{,} \end{array}$$

(6) $$\begin{array}{c} \log\left(\frac{A}{A_{0}-A}\right)=\log\left(\frac{1}{\left[DNA\right]}\right)+\log\left(\frac{1}{{{\;K}_{b}}_{K_{b}}}\right)\log\left(\frac{1}{\left[DNA\right]}\right)=\log\left(\frac{A}{A_{0}-A}\right)+\log K_{b}\text{,} \end{array}$$

(7) $$\begin{array}{c} \Delta G_{b}^{{}^{\circ}}=–RT\;\ln K_{b}\text{,} \end{array}$$

(8) $$\begin{array}{c} \%\;H=\frac{\left({ɛ}_{b}-{ɛ}_{f}\right)}{{ɛ}_{f}}\times 100\text{,} \end{array}$$

where *ɛ*_*a*_ is represented as the apparent absorption coefficient value for the MLCT band at a specific concentration of deoxyribonucleic acid and evaluated from Abs/[complex]. ɛ_*f*_ and *ɛ*_*b*_ are absorption coefficient values for the chemical substance alone and fully interacted with deoxyribonucleic acid, respectively. ∆*A*_max_ = (*A*_max_ − *A*_0_); ∆*A* = (*A*_*x*_ − *A*_0_), where *A*_0_, *A*_*x*_, and *A*_max_ are denoted as the absorbance of chemical substance alone, the middle form, and the completely interacted form with deoxyribonucleic acid, respectively.

#### 3.2.3. Assessment of Thermal Denaturation Characteristics

**Figure 3:**
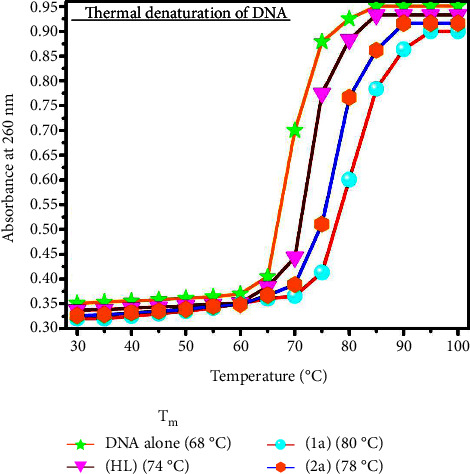
DNA thermal denaturation profile at 260 nm in the absence and presence of compounds in 5 mM Tris-HCl/50 mM NaCl buffer pH = 7.2, [DNA]/[Complex] = 1 (R).

**Figure 4:**
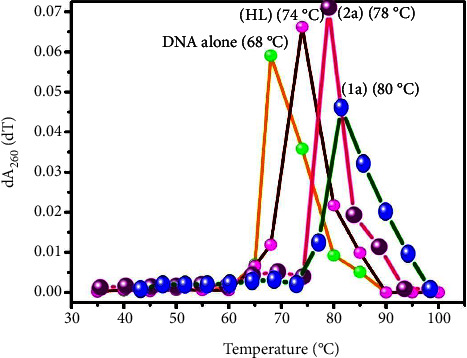
Derivative melting curve for DNA thermal denaturation at 260 nm in the absence and presence of compounds in 5 mM Tris-HCl/50 mM NaCl buffer pH = 7.2, [DNA]/[Complex] = 1 (R).

DNA denaturation contributes to the root cause of several chronic diseases, hereditary disorders, and a reduction in the ability of DNA repair to work properly. The biothermodynamic properties were further supported to determine the ability of stabilization of the double-standard DNA, and it offers details on the structural alterations, the degree of the DNA-compound system, the external binding-mediated neutralization of phosphate charges on DNA, and the stacking interactions, all of which work together to raise the DNA's melting point [65]. Moreover, in this case, it is observed that complex-DNA adducts have a higher melting temperature compared to free DNA. Complex-bound deoxyribonucleic acid is more challenging to melt compared to deoxyribonucleic acid alone because it is involved in powerful intercalation binding to DNA. The Van't Hoff (9) and Gibbs Helmholtz equations (10) were supportive in evaluating the biothermodynamic parameters, which are enclosed in Table 2. The evaluated *T*_*m*_ values of the DNA-compound adduct in all cases were obtained in the following sequences: 80°C (**1a**) > 78°C (**2a**) > 74°C (**HL**) > 68°C (**DNA** alone) ± 2°C and the value of Δ*T*_*m*_°C: 12 (**1a**) > 10 (**2a**) > 6 (**HL**). If Δ*T*_*m*_ > 10°C, the described biothermodynamic properties are also advantageous for the intercalation mode of the mechanism between the test compounds and deoxyribonucleic acid, except for the free ligand. Conversely, the result reveals the electrostatic or groove binding mode when Δ*T*_*m*_ < 10°C [66, 67]. Additionally, the complex-DNA adduct's reported negative binding free energy was lower compared to the sum of the ligand-DNA binding energies, which attributes the complexes (**1a**-**2a**) spontaneously intercalating DNA. DNA thermal denaturation profile and its derivative melting curve for thermal denaturation at 260 nm in the absence and presence of test compounds are shown in Figures 3 and 4. The influencing factors between test compounds and DNA mostly depend on the type of interaction mode. Because of the driving forces, H-bonds, weak Van der Waals forces, and electrostatic modes of binding all occur while the enthalpy is favourable. Hydrophobic forces induce binding while entropy is favourable; on the other hand, the loss of structural degrees of freedom leads to undesirable entropic changes. As per Ross and colleagues, the findings for ΔH° and ΔS° can alternatively be derived in the following favourable sequence. If ΔH° > 0 and ΔS° > 0, this attributes intercalation due to hydrophobic forces of attraction. If ∆H° < 0 and ΔS° < 0, this involves weak Van der Waals forces of attraction and H-bonding interactions. On the other hand, if ΔH° < 0 (or), ΔH°≈0 and ∆S° > 0, this indicates the electrostatic mode of binding possible between DNA and compounds [28, 68]. The measured values for all samples were exposed to the favourable sequence ΔH° < 0 and ΔS° < 0, which is assumed to be due to weak van der Waals forces of attraction and H-bonding between DNA and chemical substances. However, they lose the ability to rotate and translate, interfere with counter ions and hydrophobic forces in compound-DNA adduct, and may result in exothermically active negative signals of ΔS° and ΔH°. Furthermore, it is widely acknowledged that the hydration and generation of the compound-deoxyribonucleic acid adduct system *via* the counter ion liberating mechanism are highly dependent on the hydrophobic forces of attraction. As a result, higher negative results of ΔH° and ΔS° for all substances that interacted with DNA were observed in the experiment [69]. According to the Ross and Subramanian mechanism for protein/DNA-complex interactions, it clearly reveals that the complexation of the metal center with the morpholine fused primary aromatic and 2,2′-bipyridine secondary aromatic planar systems stimulates the silky infiltration of the complex within deoxyribonucleic acid base pairs owing to *π*-*π* stacking interactions. Additionally, in the complex-DNA adduct, a number of noncovalent molecular interactions, including dipole-dipole interaction, weak van der Waals forces of attraction, formation of H-bonding, and electrostatic forces of attraction, may be present while the complex is positively charged and engages in stacking interaction as per Manning and Record's polyelectrolyte hypothesis [70].

CT-DNA melting temperature (*T*_*m*_) = 68°C; Δ*T*_*m*_ is denoted as melting temperature changes between compounds and free DNA; ln [*K*_2_/*K*_1_]=−Δ*H*°/*R*[1/*T*_2_ − 1/*T*_1_], and enthalpy change (ΔH°) = *R*. [*T*_*m*_*T*_*r*_/*T*_*m*_ − *T*_*r*_] ln [*K*_*m*_/*K*_*r*_], *T*_1_ = *T*_*r*_ ⟶ 298 K, *T*_2_ = *T*_*m*_ ⟶ DNA melting temperature of compounds, universal gas constant (*R*) = 1.987 cal·K^−1^·mol^−1^ (or) 8.314 J·K^−1^·mol^−1^; Gibb's free energy (ΔG°) = −R. *T*_*m*_. ln*K*_*m*_; entropy change (ΔS°) = [∆H° − ∆G°/*T*_*m*_];

(9) $$\begin{array}{c} \ln\left[\frac{K_{2}}{K_{1}}\right]=\left[\frac{∆H^{{}^{\circ}}}{R}\right]\left[\frac{T_{2}-T_{1}}{T_{1}\;T_{2}}\right]\text{.} \end{array}$$

Gibb's free energy is as follows:

(10) $$\begin{array}{c} \Delta G^{{}^{\circ}}=–R.T_{m}.{\ln\;K}_{m}\text{.} \end{array}$$

#### 3.2.4. Assessment of DNA-Binding Affinity Using Viscometric Techniques

Hydrodynamic findings can be utilized to assess the alteration of DNA length and afford details on the binding tendency between small molecules and biomolecules. As a result of their sensitivity to DNA contour length changes, which implies that the average distance between each monomer (cl = 0.338 nm/bp for B-form DNA), the DNA binding properties of all test compounds were further validated by this method. According to the Lerman concept, interactions between small molecules and deoxyribonucleic acid *via* covalent and noncovalent bindings are often possible. When a chemical substance interacts with the deoxyribonucleic acid double helix, the contour length of DNA increases *via* intercalation and leads to an increase in the absolute viscosity. Meanwhile, if DNA viscosity is not affected during interactions, which leads to the responsible for the major/minor groove binding due to H-bond/Van der Waals interactions, electrostatic, partial, or nonclassical interaction modes. Therefore, analyzing the binding modes provides additional support for the findings as well as a crucial aspect of the conventional intercalation concept. In this case, it was observed that the absolute viscosity increased consistently along with the concentration of each substance at the fixed DNA concentration. The DNA must extend to facilitate the binding of ligands, which causes a considerable rise in DNA viscosity while small molecules intercalate into the DNA helix [71]. According to equation (12), an increase in relative viscosity denotes an intercalation-induced lengthening of the deoxyribonucleic acid base pair. While adhering to the principle of excluding the nearest neighbors, intercalation entails inserting a planar molecule into a DNA base pair without rupturing the hydrogen bonds that hold the base pairs together. This causes a diminution in the DNA helical twist and an extension of the DNA. Moreover, a molecule can be presumed to bind to deoxyribonucleic acid by intercalation between base pairs if it causes lengthening and unwinding of the deoxyribonucleic acid base pairs [72]. In this case, the relative viscosity of DNA gradually increases, while the concentration of deoxyribonucleic acid increases. Moreover, the affinity interactions and their slope values were observed from the relative specific viscosity (*η*⁄*η*_0_)^1/3^ plotted straight line contrasting [Compound]/[DNA], and absolute specific viscosity of deoxyribonucleic acid in the absence or presence of samples was evaluated using equation (12) (Table 3). In the experiment, it was clearly noted that the slope values for all samples also increased due to the rising binding affinity. The evaluated slope findings were in the subsequent sequence: (**EB**) 1.215 > (**1a**) 0.915 > (**2a**) 0.630 > (**HL**) 0.490 (Figure S21 and Table 3). However, complex (**1a**) exhibited superior binding affinity among the others and was substantially smaller than **EB**. Due to the existence of the 2,2′-bipyridine and morpholine-fused aromatic planar systems, compounds can interact with deoxyribonucleic acid robustly *via* intercalation. The outcomes were excellent in accordance with the observed outcomes of absorption spectral properties.

(11) $$\begin{array}{c} {\left(\frac{\eta }{\eta_{0}}\right)}^{1/3}=\frac{\left\{\left(t_{complex}-t_{0}\right)/t_{0}\right\}}{\left\{\left(t_{DNA}-t_{0}\right)/t_{0}\right\}}\text{,} \end{array}$$

where *η* and *η*_0_ are represented as specific viscosity of DNA in the presence complex and specific viscosity of DNA alone, and *t*_0_, *t*_DNA_, and *t*_complex_ are represented as the average flow time of the Tris-HCl buffer solution, the average flow time of DNA alone solution, and the average flow time of DNA interacted with the samples, respectively. Error limit is ±2.5% (*P* < 0.025).

#### 3.2.5. Assessment of DNA-/BSA-Binding Characteristics Using Emission Titration

**Figure 5:**
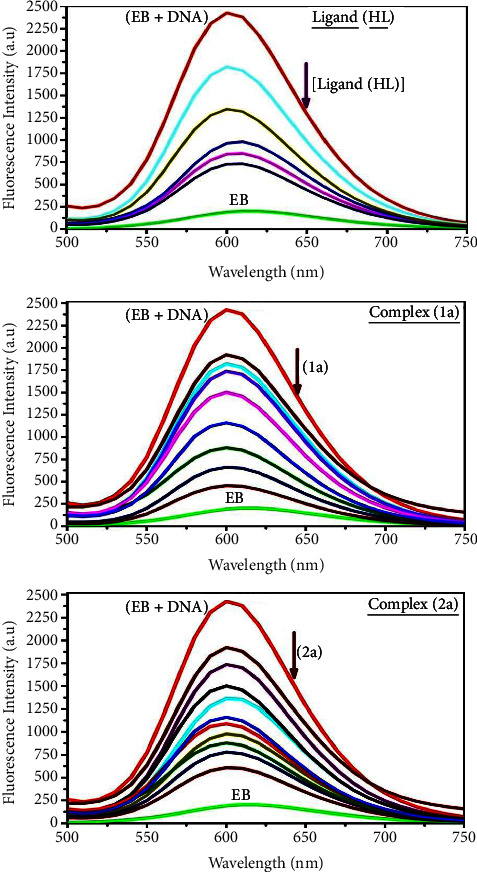
Fluorescence quenching curve of ethidium bromide bound DNA in the presence of ligand (**HL**) and complexes (**1a**-**2a**).

Emission titration is a more sensitive approach to examining the binding tendency between chemical substances and biomolecules. The emission measurements are extensively supported to scrutinize the interacting modes between compounds and deoxyribonucleic acid. In the presence of deoxyribonucleic acid (240 *μ*M) in a Tris-HCl buffer solution (pH: 7.2) at room temperature, none of the test compounds emitted fluorescence. The titrations were also executed with the EB molecule, which is also a comparatively low fluorescence emission in Tris-HCl buffer solution (pH: 7.2) in the free state. Owing to the effective intercalation of EB with CT-DNA, the EB fluorophore displays extremely bright fluorescence at about 610 nm [73]. In the investigation, the addition of sample concentrations (0–240 *μ*M) to the solution of DNA-EB results in a notable reduction in the emission intensity at 610 nm (Figure 5 and Table 4). As a result of intense intercalation, the complexes displace the EB in the DNA-EB adduct, causing the emission intensity to drop. The photoelectron shift from the DNA's guanine base to the excited states may be the cause of the frequency of quenching in the emission of chemicals by DNA. Therefore, EB can be utilized as a fluorescent probing agent in the competitive binding experiment.

*K *_*SV*_ is denoted as Stern–Volmer binding constant; *K*_ass_ is represented as association binding constant; *K*_app_ is represented as apparent binding constant, *K*_app_=*K*_EB_[EB]/[compound]=500/[compound], *K*_EB_ = 10^7^ M^−1^ at the concentration of 50 *μ*M EB; Gibb's free energy change ΔG_b_°=–RT ln*K*_ass_; *K*_*q*_ is represented as bimolecular quenching rate constant/Stern–Volmer dynamic quenching rate constant (K_*q*_=K_*SV*_/*τ*_0_), average life time of the biomolecular quenching in the absence of a quencher (*τ*_0_) = 10^−8^ S; Gibb's free energy change ΔG_b_°=–RT ln*K*_ass_ (where *R* = 8.3144 KJ·mol^−1^, *T* = 298 K); *K*_LB_ is represented as Lineweaver–Burk (LWB) binding constant; *K*_SA_ is represented as Scatchard association binding constant; *K*_app_ is denoted as apparent binding constant; *n* is the number of binding sites; *P* is a ratio of fluorescence quantum efficiency of DNA bound and free complex (*P*=ɸ_*b*_/ɸ_*f*_), which is obtained as intercept from plot *F*/*F*_0_* vs*. 1/[DNA], Error limit is  ± 2.5% (*P* < 0.025).

(12) $$\begin{array}{c} F_{corr}=F_{obs}\times e^{{{\left[\left(A\right)\right.}_{ex}\times \left(d_{ex}\right)+\left(A\right)}_{em}\times \left(\left(d_{em}\right)\right]/2}=F_{obs}\times e^{\left({A_{ex}+A}_{em}\right)/2}\text{,} \end{array}$$

where *F*_corr_ and *F*_obs_ are represented as the IFE-corrected fluorescence and observed (uncorrected) emission intensities, respectively. *d*_ex_ and *d*_em_ are denoted as the cuvette path lengths in the excitation and emission directions, respectively. *A*_ex_ and *A*_em_ are represented as the change in absorbance at the excitation and fluorescence wavelengths, respectively.

(13) $$\begin{array}{c} \frac{F_{0\;}}{F}=1+K_{SV}\left[Q\right]=1+K_{q}\;\tau_{0}\text{,} \end{array}$$

where [*Q*] is represented as the sample concentration, the emission intensities *F*_0_ and *F* of DNA/BSA in the absence and presence of the quencher (sample), respectively.

(14) $$\begin{array}{c} \frac{F_{0\;}}{F}-1=\frac{F_{0}-F}{F}=K_{SV}\left[Q\right]\log\left(\frac{F_{0}-F}{F}\right)=\log K_{ass}+n\;\log\left[Q\right]\text{,} \end{array}$$

(15) $$\begin{array}{c} K_{EB}\left[EB\right]=K_{app}\left[compound\right]\text{,} \end{array}$$

(16) $$\begin{array}{c} \left(\frac{1}{F_{0}-F}\right)=\frac{1}{{F_{0\;}K}_{LB}\left[Q\right]}+\frac{1}{F_{0\;}}\text{,} \end{array}$$

(17) $$\begin{array}{c} \left(\frac{\gamma }{C_{F\;}}\right)=K_{SC}\left(n-\gamma \right)\text{,} \end{array}$$

where *γ* = [(*F*_0_ − *F*)/*F*_0_], and *C*_*F*_ is denoted as the concentration of sample alone.

Additionally, the intensity of bovine serum albumin was monitored at 350 nm (*λ*_ex_ = 278 nm) during fluorescence titration. When increasing the sample concentrations, the BSA intensity diminishes dramatically owing to static quenching in the ground state. It is noted that the fluorophores of BSA are not clearly exposed to a shift in polarity [74–76]. The fluorescence spectra of bovine serum albumin with a variety of concentrations of all samples were estimated, and they are displayed in Figure S26. Additionally, the Stern–Volmer equations (12)-(13) were employed to analyze the data (Figure S27 and Table 4). Additionally, the observed kq values for DNA and BSA binding were found in the range of 1.1636–5.2474 × 10^12^ and 2.6390–8.6910 × 10^12^ mol^−1^·s^−1^, respectively. They are also much exceeded compared to the collision quenching constant value (2.0 × 10^10^ mol^−1^·s^−1^). Therefore, it is assumed that the static quenching process was brought on by adduct construction between the compounds and bovine serum albumin rather than a dynamic collision. However, fluorescence spectroscopy is generally plagued by the inner filter effect (IFE), which disturbs the spectral analysis in particular. The energizing ray is attenuated due to the highly concentrated solution sample. As a result, strong fluorescence is only seen on surfaces facing the excitation beam. The fluorescence intensity is reduced as a result of an inner filter effect generated by some chemicals' absorption of the excitation or emission wavelength in the UV province. The observed values of the absorption wavelength of ligand (**HL**) and mixed ligand complexes (**1a**-**2a**) in the range of 335–337 nm, the bovine serum albumin excitation wavelength of 278 nm, and emission wavelength of 350 nm were used to assess the effect of IFE in this approach. Equation (12) is employed to solve IFE in this instance as well (Table 4) [77, 78].

The fluorescence emission intensities of ethidium bromide interacted with deoxyribonucleic acid at 610 nm, and those of bovine serum albumin at 350 nm exhibited a distinctly reducing movement with increasing concentrations of the test compounds after resolving the inner filter effect (IFE). Additionally, no emission spectrum shifting was seen following the BSA-complex adduct, indicating that ground state BSA-compound systems formed owing to a static quenching mechanism. It is also observed that BSA might interact with complexes and that the polarity of BSA's fluorescence did not vary noticeably with complex titration. The linear regression correlation coefficient (*R*^2^) values for all substances further confirmed that there is no found inner filter effect due to these values are greater than 0.95. Moreover, the following Stern–Volmer equations (13)–(15) were employed to determine the *K*_SV_, *K*_*q*_, and *n* values (Table 4). The *K*_SV_ findings were measured from the slope to intercept ratio of the linear regression plot of *F*_0_/*F vs*. [*Q*] by the SV method I (Figure S22). Equation (14) is employed to evaluate the “*n*” and *K*_ass_ values [42]. Similarly, *K*_app_ (apparent binding constant) values for all samples were estimated using equation (15) (Table 4). The values of *K*_ass_ and *n* were determined from the antilogarithm of the intercept and slope findings, respectively, in the linear regression plot of log (*F*_0_ − *F*)/*F vs*. log [*Q*] by SV method II with the help of equation (14) (Figure S22 and Table 4). *ε* findings for all substances were observed from the negative slope in the linear regression plot of emission intensity *vs*. [compound] with the help of the Beer–Lambert law equation (*A* = *ε*cl) (Figure S25), and equation (15) is supported to evaluate *K*_app_ value using *K*_EB_ = 10^7^ M^−1^ at 50 *μ*M concentration and measured sample concentrations for all cases using the Beer–Lambert law equation. The Lineweaver–Burk (16) and Scatchard analysis (17) are utilized to expand the observations and validate the binding affinities [79, 80], and the observations are also compared with the Stern–Volmer method (Table 4). Equation (16) is used to determine the *K*_LB_ from the finding of intercept divided by slope in the linear regression plot of 1/(*F*_0_ − *F*) *vs*. 1/[*Q*] (Figure S22 and Table 4). The linear regression plot of (*γ*/*C*_*F*_) *vs*. *γ* is employed to support equation (17), which was used to determine the values of *K*_SA_ and *n* from the negative sign of the slope and the finding of intercept divided by the slope, respectively (Figure S23), and the overall measured DNA/BSA *K*_*b*_ findings in all cases were in the subsequent sequence: (**1a**) > (**2a**) > (**HL**). The “*n*” findings for deoxyribonucleic acid and bovine serum albumin binding acquired from the Stern–Volmer equation (14) were found in the range of 0.9733–1.1682 and 0.9260–1.0590 for all compounds, respectively. Moreover, the obtained *n* values for DNA binding by the Scatchard equation (17) were in the range of 0.9711–1.0966, and these values are nearly equal to one (Table 4). In these cases, complex (**1a**) has shown a better binding affinity among others. Consequently, it is proposed that the complexes contain both a 2,2′-bipyridine ring planar system and an aromatic ring system linked to morpholine. They can effectively interact with deoxyribonucleic acid *via* intercalation. Additionally, the values of the fluorescence quantum efficiency (*P*) ratio for the DNA-compound and BSA-compound adducts were 0.0900–0.2000 and 0.252–0.564, respectively. The findings were measured from the intercept in the linear regression plot of *F*/*F*_0_* vs*. 1/[DNA] and 1/[BSA], respectively (Figures S24 and S28 and Table 4). The findings and those from the viscosity, electrochemical titration, and UV-vis spectral properties were in good accordance with the outcomes.

#### 3.2.6. Förster's Theory-Based FRET Computation

**Figure 6:**
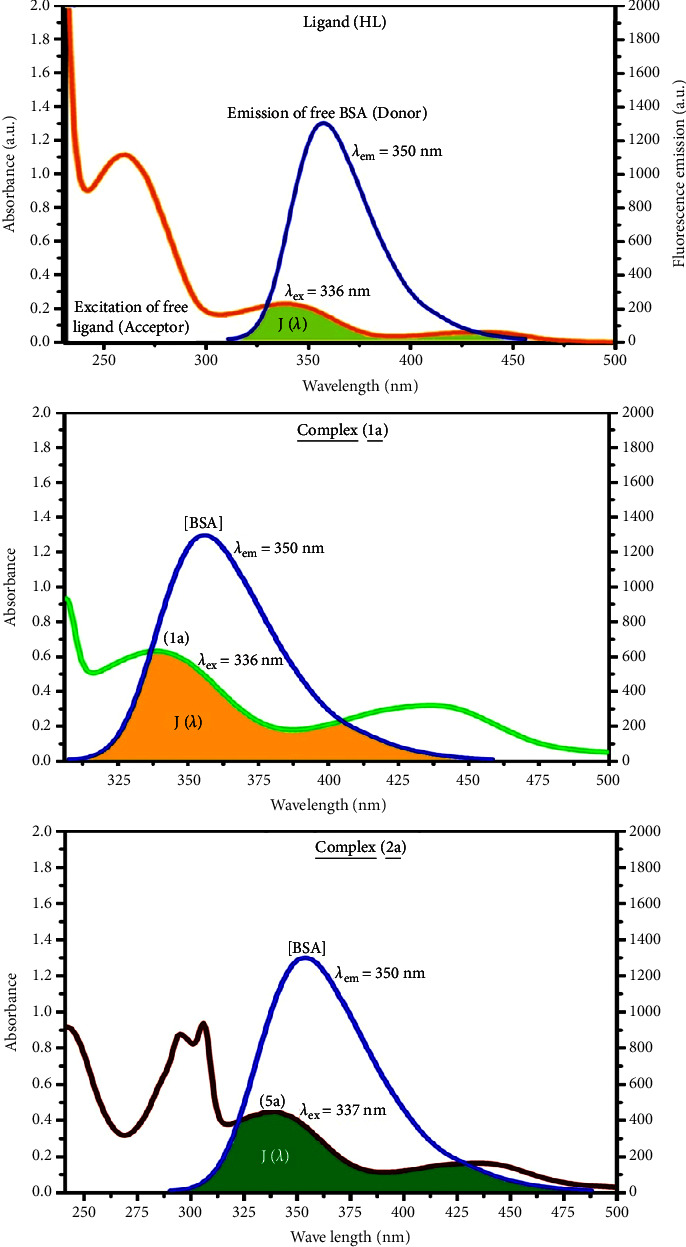
The overlap of UV-vis spectra of ligand (**HL**) and mixed ligand complexes (**1a**-**2a**) (**acceptor**) at 336–337 nm with fluorescence emission spectrum of BSA (**donor**) at 350 nm.

FRET can be executed to distinguish the relative orientation and closeness of fluorophores [81]. The process happens when there is a large overlapping of the acceptor's (compound) absorption spectrum with the donor's (BSA) emission spectrum. Fluorescence is quenched owing to energy being transmitted from the excited state of bovine serum albumin to substances (**HL**)/(**1a** − **2a**). As a result of the FRET analysis, their observed “*r*” findings were found in the range of 2.1851–2.7127 nm (Table 5 and Figure 6). It also shows that there is a high probability that energy will be transferred from bovine serum albumin to the compounds. The following conditions have a major impact on the FRET's effectiveness: (i) the distance (*r*) should be within the prescribed range from 2 to 8 nm for energy transfer, (ii) there is a large overlapping of the emission spectrum of biomolecules (donors) with the electronic absorption spectrum of acceptors (substances), and (iii) the bovine serum albumin and substance transition dipoles are oriented correctly. Bovine serum albumin transmits excitation energy to a compound during FRET without emitting a photon from the previous molecule system. It is a mechanistic pathway between several electronic excited states of molecules that depends on distance. Equation (18) can be employed to estimate the efficiency of energy transfer (*E*) in accordance with the FRET approach and the Förster radius (*R*_0_) for the donor-acceptor system, which is evaluated from equation (19) (Table 5). The relative orientation factor of the dipoles (*K*^2^) is associated with the geometry of the BSA and the complex of the dipoles, the value for random orientation (*K*_2_ = 2/3) like in a fluid solution. In short, the *K*^2^ values were found in the range from 0 to 4, and energy can be transferred from the BSA to the compound when electrons are transferred between the two molecules. For parallel transition dipoles that are aligned, *K*^2^ is equal to 4, which denotes the maximal energy transfer, and when the orientation of the dipoles is perpendicular to one another, *K*^2^ is equal to 0, which denotes very weak energy transfer. When the relative orientation of the dipoles is at random, *K*^2^ is attained to be equal to 2/3. *n* is denoted as the average refracted index of the medium, Φ is represented as the fluorescence quantum yield of the BSA, and equation (20) is helpful to measure the normalized spectral overlap integral (*J*) for the overlapping emission spectrum of the BSA with the electronic absorption spectrum of the compound (Table 5). The following variables for the complex-BSA interaction are determined using equations (18–23), *n* = 1.36, Φ = 0.15, *E* = 0.3462–0.5769, *J* = 0.5425–0.8215 × 10^−14^ cm^3^·L·mol^–1^, *R*_0_ = 2.2770–2.4400 nm, *r* = 2.1851–2.7127 nm, *k*_ET_ = 5.2957–13.6340 J/s, and *B* = 4642.53–5339.79 mol^−1^·cm^−1^ (Table 5). The observed findings of *R*_0_ and *r* between BSA, Trp213, and the interacted compound were substantially smaller than 8 nm, and their relationships are found in the following sequence: 0.5*R*_0_ (1.1384–1.2200) < *r* (2.1851–2.7127) < 1.5*R*_0_ (3.4155–3.6600). This implied that there was a sturdy possibility that the test compound and BSA exchanged nonradiative dipole-dipole energy, which agreed with a static quenching mechanism. This result proved that the binding adhered to Förster's energy transfer theory's conditions. Φ is denoted as the quantum yield, which is ascribed as the dimensionally invariant ratio of emission and absorption photons by a fluorophore, and it serves as a tool for estimating fluorescence emission's effectiveness in correlation to all other channels of relaxation. In addition, *τ* is denoted as the lifetime of fluorescence emission of the biomolecule and is described as the inverse of the entire degradation rate *τ* = 1/(*k*_*r*_ + *k*_nr_). The radiative lifetime of the fluorophore is represented as *τ*_0_ = 1/*k*_*r*_. The values of *τ* and Φ are associated with equation (21) (Table 5). Quenching occurs while a BSA's ground or excited states come into contact with a compound in the solution. There is also a diminishing in the fluorescence emission intensity. They are divided into two main categories of dynamic and static quenching. While an excited state BSA binds to the substance during a dynamic or collisional quenching mechanism, the BSA is radiationlessly deactivated to the ground state. Therefore, the concentration of the quenching compounds affects the dynamic quenching. The *τ* and Φ values for BSA diminished with raising the compound concentration. Conversely, static quenching minimises emission without changing the excited state *τ* or Φ, and quenching can be divided into two main categories based on the excited-state lifetime of the fluorophore. Additionally, the term *k*_*q*_ [*Q*] is included in the denominator in equation (21), and the Φ value for the bovine serum albumin-compound adducts system is measured by equation (21). FRET requires an interaction between the emission and the absorption transition dipole moments of the bovine serum albumin and test compound, respectively, due to the nonradiative transfer of excitation energy from a fluorophore to a chromophore [82]. *k*_ET_ is denoted as the rate of energy transfer, which depends on not only the spectrum overlapping of the emission of the BSA and the absorbance of the compound but also the Φ value of BSA, *K*^2^, and *r* etc. The *k*_ET_ values for all substances were estimated by equation (22) [83]. Additionally, the brightness of BSA depends on the capability of a test compound to absorb light and the Φ value, which is calculated by the expression (23) (Table 5). Chemical compounds with high absorbance have higher values for *ε* and Φ, which also promotes effective emission.

FRET ⟶ Fluorescence resonance energy transfer,

(18) $$\begin{array}{c} E=\left(1-\frac{F}{F_{0}}\right)=\left[\frac{{\;R}_{0}^{6}}{\left({\;R}_{0}^{6}+r^{6}\right)}\right]\text{.} \end{array}$$

When transmit efficiency is 50%, the observed critical distance is *R*_0_, which is denoted as the Förster radius characterizing the donor/acceptor pair and is evaluated from the equation (19).

(19) $$\begin{array}{c} R_{0}^{6}=8.79\times 10^{-25}K^{2\;}n^{-4}\Phi J\text{,} \end{array}$$

(20) $$\begin{array}{c} J=\left[\frac{\int_{0}^{\infty }F\left(\lambda \right)\varepsilon \left(\lambda \right){\;\lambda }^{4}d\lambda }{\int_{0}^{\infty }F\left(\lambda \right)d\lambda }\right]\text{,} \end{array}$$

where *J* is denoted as normalized spectral overlap integral between the emission spectrum of donor (BSA) and the absorption spectrum of acceptor (complex), *R*_0_ is critical distance at which the efficiency of resonance energy transfer (50%) $R_{0}=\sqrt[{6}]{0.2569\times 10^{-25}J}$, average refracted index of medium (*n*) = 1.36, fluorescence quantum yield of the donor (Φ) = 0.15, orientation factor related to geometry of the donor and acceptor of the dipoles (*K*^2^) = 2/3 for the complex-BSA interaction, *E* is represented as efficiency of energy transfer, *E*=(1 − *F*/*F*_0_), (*f*) and *F*_0_ are the fluorescence intensity of BSA in the presence and absence of complex, r is the donor-acceptor separation relative to their van der Waals radii *L* (nm), $r=\sqrt[{6}]{\left[\left(R_{0}^{6}/E\right){-R}_{0}^{6}\right]}=R_{0}\left(1/E-1\right)$, (*f*) (*λ*) is represented as the corrected or the normalized emission intensity of the BSA in the wavelength range of *λ* − (*λ* + ∆*λ*), *ε* (*λ*) is denoted as the molar absorption coefficient of the compound at *λ*.

(21) $$\begin{array}{c} \Phi =\frac{\tau }{\tau_{0}}=\frac{k_{r}}{k_{r}+k_{nr}}=\frac{k_{r}}{k_{r}+k_{nr}+{\;k}_{q\;}\left[Q\right]}\text{,} \end{array}$$

where the radiative, nonradiative decay and quenching rate constants are denoted as *k*_*r*_, *k*_nr_, and *k*_*q*_, respectively, *τ*_0_ ⟶ radiative lifetime of the fluorophore (biomolecules) (*τ*_0_ = 10^−8^ s), and the concentration of complex (quenching species) is described as [*Q*].

(22) $$\begin{array}{c} k_{ET}=\frac{1}{\tau_{0}}{\left(\frac{R_{0}}{r}\right)}^{6}=KJ{\;e}^{\left(-2r/L\right)}\text{,} \end{array}$$

*K* is detonated as a relative factor of the specific orbital interactions based on the orbital overlap between the bovine serum albumin and substances.

(23) $$\begin{array}{c} B=\Phi \;\varepsilon \text{.} \end{array}$$

*k*_ET_ is denoted as rate of exchange resonance energy transfer, *B* is denoted as average brightness of the complex-BSA system, *B* = [(Φ_1_*ε*_1_ + Φ_2_*ε*_2_)/2], *ε* is molar absorption (or) extinction coefficient of the acceptor at *λ*, *ε* = 43,824 LM^−1^·cm^−1^ for donor (BSA) and *ε* values for acceptors = 27,373.20 (**HL**), 25,346.40 (**1a**), and 18,076.40 (**2a**). *B* value of free BSA = 6,573.60 M^−1^·cm^−1^.

#### 3.2.7. Analysis of DNA-Binding Characteristics Using the CV Method

The CV technique is one of the most important tools for investigating the DNA-complex adduct's binding mechanism. The CV properties of all test samples in the presence and absence of DNA were executed at a scan rate (*v*) 0.1 Vs^−1^ with a potential range of +2 to −2 in a Tris-HCl (5 mM)/NaCl (50 mM) (pH = 7.2) solution. The M^1+^/M^2+^ redox couple is caused by complexes that reveal a single anodic and cathodic peak. The complex's reaction with the glassy carbon electrode surface was shown to be a one-step, one-electron, quasireversible redox process since the redox couple's (*I*_pa_/*I*_pc_) ratio values were close to unity, which is also supported by the change in peak potential separation (*E*_*p*_ > 0.0591 V) [84–86] (Figure S29 and Table 6).

Δ*E*_*P*_ is peak-to-peak separation = (*E*_Pa_ − *E*_Pc_); *E*°(or) *E*_1/2_ is denoted as formal electrode potential = 1/2 (*E*_Pa_ + *E*_Pc_); *E*_*s*_°=(*E*_*b*_° − *E*_*f*_°)*E*_*b*_° and *E*_*f*_° are represented as the formal electrode potential of the M^1+^/M^2+^ couple in the bound and free forms, respectively. *E*_*s*_° = +19 mV (**HL**), +34 mV (**1a**), and +31 mV (**2a**). *I*_pa_ is anodic peak current, *I*_pc_ is cathodic peak current. K^1+^ is binding constant of reduction process, K^2+^ is binding constant of oxidation process, *S* is represented as binding site size of base pairs (bp) with a molecule of complex, Scan rate is 100 mV·s^−1^, binding constant (*K*_*b*_) values observed from the linear plots of log (1/[DNA]) *vs*. log (*I*/*I*_0_ − *I*) for oxidation and reduction, (*I*_0_ − *I*_DNA_)/*I*_DNA_ = *C*_*p*_/*C*_f_* versus* [DNA] and *I*_p _^2 ^*versus* (*I*_po _^2 ^−*I*_p _^2 ^)/[DNA] by methods I, II, and III, respectively.

(24) $$\begin{array}{c} Diffusioncoefficient\left(D_{0}\right)=\sqrt{7.51\times 10^{-5\;}\left(Slope\right)\log\left(\frac{I_{0\;}-I}{I}\right)=\log\;\left[DNA\right]+\log K_{b},} \end{array}$$

where *I*_0_ and *I* are represented as the peak currents of the compound in the absence and presence of DNA.

(25) $$\begin{array}{c} \log\;\left[DNA\right]=\log\left(\frac{I_{0\;}-I}{I}\right)+\log\frac{1}{K_{b}}\;\log\left(\frac{1}{\left[DNA\right]}\right)=\log\left(\frac{I}{I_{0}-I}\right)+\log K_{b}\text{,} \end{array}$$

(26) $$\begin{array}{c} \left(\frac{I_{0\;}-I}{I}\right)=K_{b}\left[DNA\right]\text{,} \end{array}$$

(27) $$\begin{array}{c} \left(\frac{I_{0\;}-I}{I}\right)=\left(\frac{C_{b}}{C_{f}}\right)\text{,} \end{array}$$

where *C*_*f*_ and *C*_*b*_ are denoted as the free substance concentration and DNA-interacted compound, respectively. (29) was obtained by comparing equations (27) and (28).

(28) $$\begin{array}{c} \left(\frac{C_{b}}{C_{f}}\right)=K_{b}\left[DNA\right]\text{,} \end{array}$$

(29) $$\begin{array}{c} \left(\frac{C_{b}}{C_{f}}\right)=\frac{K_{b}\left[freebasrpairs\right]}{S\;}\text{,} \end{array}$$

(30) $$\begin{array}{c} \left(\frac{C_{b}}{C_{f}}\right)=\frac{K_{b}\left[DNA\right]}{2\;S\;}+1\text{.} \end{array}$$

*S* is denoted as binding site size (bp), and *K*_*b*_ are estimated from (31) with the help of *S* = (intercept/4)^1/2^ and (*K*_*b*_ = 2*S* (slope/intercept), respectively. Nernst equations are as follows:

(31) $$\begin{array}{c} E_{s}^{{}^{\circ}}=\left({E_{b}^{{}^{\circ}}-E}_{f}^{{}^{\circ}}\right)=\frac{0.0591\;}{n\;}\log\left(\frac{K_{\left[red\right]}}{K_{\left[oxi\right]}}\right)\text{,} \end{array}$$

(32) $$\begin{array}{c} \left(\frac{K_{\left[red\right]}}{K_{\left[oxi\right]}}\right)=Ant.\;\log\;n\left(\frac{\left({E_{b}^{{}^{\circ}}-E}_{f}^{{}^{\circ}}\right)}{0.0591}\right)\text{.} \end{array}$$

*E *_1/2_ or *E*_*b*_° and *E*_*f*_° are the formal electrode potentials of the M^1+^/M^2+^ couple in their bound and free forms, respectively.

(33) $$\begin{array}{c} I_{p\;}^{2\;}=\frac{1}{K_{b}\left[DNA\right]}\left(I_{p_{0}\;}^{2\;}-I_{p\;}^{2\;}\right)+I_{p_{0}\;}^{2\;}-\left[DNA\right]\text{,} \end{array}$$

where *I*_*po*_ and *I*_*p*_ are denoted as the peak currents of the complexes (1–3) in the absence and presence of DNA.

(34) $$\begin{array}{c} I_{pa}=2.69\times 10^{5\;}n^{3/2\;}\alpha^{1/2\;}AC_{0\;}^{*}D_{0\;}^{1/2\;}v^{1/2\;}\text{,} \end{array}$$

(35) $$\begin{array}{c} I_{pa}=13314.7D_{0\;}^{1/2\;}v^{1/2\;}\text{,} \end{array}$$

where *I*_pa_ is denoted as the anodic peak current in amperes, *n* is represented as the number of electrons participating in the redox (M^1+^/M^2+^) process (*n* = 1), charge transfer coefficient (or) activation coefficient (*α*)≈0.5 for quasireversible systems, which also calculated from Bard–Faulkner relation

(35a) $$\begin{array}{c} \alpha =\left[47.7/E_{Pa}-E_{P/2}\right]\text{.} \end{array}$$

C_0_^*∗*^ ⟶ Bulk concentration of the compound, *A* is denoted as the cross-sectional area of the working electrode (glassy carbon) in cm^2^ (*A*∼0.07 cm^2^), *D*_0_ is denoted as diffusion coefficient (cm^2^ s^−1^) of the M^1+^/M^2+^ couple in the free and bound forms, respectively, and *v* is denoted as the potential scan rate at 0.1 Volt·s^−1^. While the substances often bind to deoxyribonucleic acid through intercalation, the peak potential shifts in a positive direction. When the compounds bind to deoxyribonucleic acid *via* minor or major grooves or electrostatic attractions, the peak potential shifts occur in a negative direction. In this case of ligand (**HL**) and its complexes (**1a**-**2a**), due to the consistent movement in the positive direction caused by the increment of deoxyribonucleic acid, the binding mode has been described as mainly intercalation in the compound-DNA adduct (Figure S29), and it is further ascribed to the presence of 2,2′-bipyridine and morpholine fused aromatic planar systems in mixed ligand complexes, which can create inclusion through intercalation owing to hydrophobic and *π*-*π* stacking interactions in the deoxyribonucleic acid base pairs. It is also verified by the evaluated outcomes of absorption titration, emission titration, viscometric, and biothermodynamic properties. Furthermore, the binding constants, binding site size (*S*), and ratio of binding constants (K^1+^/K^2+^) for M^1+^/M^2+^coupled systems further confirmed the binding affinity *via* intercalation. Additionally, the subsequent equations (24)–(35) are supported to determine the above parameters [87, 88]. Equation (24) is acquired from the modification of the Stern–Volmer equation (14) (Table 6). *K*_*b*_ values for all samples were estimated through the antilogarithm of the intercept in the linear regression plot of log (1/[DNA]) *vs*. log (*I*/*I*_0_ − *I*) by method I (Figure S30 and Table 6). Binding site size (bp) (*S*) and *K*_*b*_ are estimated from method II [89–91] (Figure S31 and Table 6). In addition, the base pair sites in a molecule of the compound are referred to as binding site size (*S*), which is also suggested that there should be one binding site for every two base pairs, and the evaluated *S* findings were found in the range from 0.1230 to 0.4520 bp (Table 6). In general, if *S* value is less than one, this denotes stronger binding through intercalation, and if *S* value is greater than one, this suggests the possibility of the mode of groove binding or electrostatic interactions [92–96]. Also, complex (**1a**) has a higher binding efficiency than others owing to robust DNA-binding affinity through intercalation with a low binding site size. It is therefore stated that a compound or medication exhibits high binding affinity when it occupies a single binding site. Meanwhile, the drug-DNA adducts exhibit low binding affinity when many site sizes are increased for the same [97]. As a result of the variable binding state [M^1+^/M^2+^] and the delayed mass transfer of complexes interacting with deoxyribonucleic acid fragments, the increment of deoxyribonucleic acid to the complex solution enables a change in the redox potential to a higher positive direction and a drop in both anodic and cathodic peak currents. Moreover, the E_s_° values of M^1+^/M^2+^ for all substances were observed to be positive values (Table 6). This suggests that the compounds' strong hydrophobicity makes their interactions with deoxyribonucleic acid through intercalation more favourable. On the other hand, if the value of E_s_° is negative, this indicates that the substance interacts more favourably with DNA through electrostatic interactions, and K^1+^ and K^2+^ are represented as binding constant findings for the binding states of the +1 and +2 chemical substances to deoxyribonucleic acid, respectively. With the aid of equation (25), the linear regression plot of log (1/[DNA]) *versus* log (*I*/*I*_0_ − *I*) (method I) is employed to determine the ratio of binding constant findings (*K*_[red]_/*K*_[oxi]_) for reduction and oxidation processes, which was also estimated using the Nernst equation (32) (Table 6). Generally, the DNA-compound adduct is assigned the groove binding or electrostatic binding interaction when the ratio of [K^1+^/K^2+^] is equal to one. When the ratio value is less or greater than one, it demonstrates that the mode of intercalation binding could occur in the DNA-compound system owing to hydrophobic forces of attraction [98, 99]. The following mechanism led to the latter finding in the compounds-deoxyribonucleic acid system (Table 6):

(36) $$\begin{array}{c} {ML}^{2+}+e^{-}\Leftrightarrow {ML}^{+}E_{f}^{0}\\ K_{2+}↿⇂↿⇂K_{+}\\ {ML}^{2+}-DNA+e^{-}\Leftrightarrow {ML}^{+}-DNA{\;E}_{b}^{0}. \end{array}$$

*K *_*b*_ finding was measured by method III (Figure S32 and Table 6). In these cases, complex (**1a**) shows greater binding effectiveness among others owing to its robust binding affinity with deoxyribonucleic acid through intercalation. As a result, it is proposed that the complexes consist of an aromatic planar linked with a morpholine moiety as well as 2,2′-bipyridine planar systems that may strongly bind to DNA through intercalation, and it is also validated by the value of the diffusion coefficient (*D*_0_) of the compound alone and the DNA-bound test substance with the aid of the subsequent quasireversible Randles–Sevcik equation (34) (Table 6) [100, 101]. On adding DNA to test compounds, the anodic/cathodic peak currents of the M (I)/M (II) reduced due to a diminishing of *D*_0_. It is clearly suggested that the evaluated values of *D*_0_ of deoxyribonucleic acid-bound samples were fewer than the free test samples. The values of *D*_0_ for all samples in the absence and presence of DNA at scan rates of 0.01–0.3 V/s were measured from the linear regression plots of _*f*_Ipa *vs*. *v*^1/2^ and _*b*_Ipa *vs*. *v*^1/2^ using equation (35) [102–104] (Figure S33 and Table 6).

### 3.3. Evaluation of BSA Binding by UV-Visible Spectral Titration

**Figure 7:**
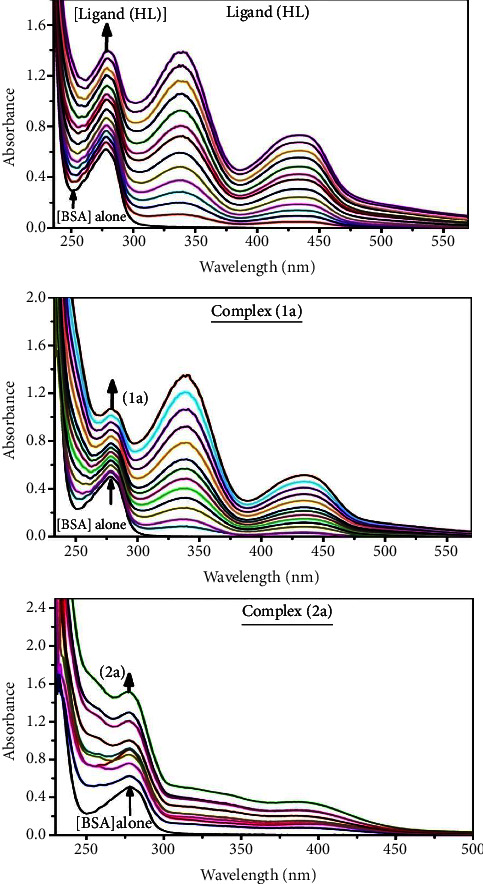
Bovine serum albumin's UV-visible titration spectra at 25°C in a Tris-HCl buffer at a pH of 7.2 in the absence and presence of rising amounts of test substances. Arrow shows the changes in absorbance upon increasing the substance concentration.

The UV-visible spectral titration properties are imperatively supported to scrutinize the structural changes and the nature of quenched biomolecules by the chemical substance. The titration was executed for BSA in the presence and absence of substances in Tris-HCl solution (Figure 7 and Table 7). Two distinctive adsorption peaks can be noticed in the UV-visible spectra of free BSA: one at 210 nm, which is connected to the polypeptide backbone, and the other at 280 nm, which is responsible for Trp, Tyr, and Phe aromatic amino acid residues. BSA's interaction with the test substances is designated by alterations in the electronic absorption spectra. Quenching typically occurs in either a static or dynamic phase. The static quenching mechanism only involves the synthesis of a bovine serum albumin-compound in the ground state, when a dynamic quenching mechanism involves the temporary presence of the excited state due to diffusion, which brings the BSA and the compound into close proximity. In addition, the dynamic quenching mechanism has no outcome on the electronic absorption spectrum; it only affects the excited state [105]. Furthermore, both types have different temperature dependences; for example, in dynamic quenching, the quenching constants are meant to rise with temperature. Conversely, raised temperatures in static quenching favour decreased stability and lower quenching constants [106]. The BSA's absorption intensity was found to be between 278 and 280 nm. When the test sample concentration raises, the absorbance value also raises accompanied by the blue shift (hypsochromic) (2–5 nm), and it suggests that a more polar microenvironment is exposed to the protein's aromatic residues [107]. It also recommended that bovine serum albumin and the test compounds in the ground state interact statically. In this case, the evaluated hyperchromism was found in the range of 47.79 to 65.62%. The results also demonstrate that conformational alterations may happen owing to noncovalent interactions like H-bonds and electrostatic binding between substances and bovine serum albumin. The Benesi–Hildebrand (37) is supported to evaluate the *K*_*b*_ values (Table 7) [108]. The *K*_app_ findings for all substances were estimated from the finding of the intercept divided slope in the linear regression plot of [(*A*_∞_ − A_0_)/(*A*_*x*_ − A_0_)] *vs*. {1/[compound]} M^−1^(Figure S34). The evaluated *K*_*b*_ findings for all test substances were in the subsequent order: (**1a**) > (**2a**) > (**HL**) with ∆*G*_*b*_° values from –22.1246 to –28.0038 kJ·mol^−1^. Complex (**1a**) is also clearly shown to have the greatest spontaneous binding efficacy with BSA among others.

*Hyperchromism*%=(*A*_*∞*_ − *A*_0_)/*A*_*∞*_ × 100; *A*_0_ is denoted as absorbance of BSA alone at 278 nm, *A*_∞_ is represented as absorbance of the fully bound form of BSA with complex or ligand, and *A*_*x*_ is absorbance of BSA in the addition of different concentration of complex or ligand, Gibb's free energy change Δ*G*_*b*_°=–*RT* ln*K*_app_ (where *R* = 8.3144 KJ·mol^−1^ and *T* = 298 K); *K*_app_ is denoted as apparent binding constant evaluated from the UV-vis absorption spectral titration.

(37) $$\begin{array}{c} \frac{\left(A_{\infty }-A_{0}\right)}{\left(A_{X}-A_{0}\right)}=\frac{1}{K_{b}\left[Compound\right]}+1\text{,} \end{array}$$

where ∆*A*_max_ = (*A*_∞_ − *A*_0_), ∆*A* = (*A*_*x*_ − *A*_0_), *A*_0_, *A*_*x*_, and *A*_∞_ are denoted as the absorbance of free BSA, the absorbance of BSA in the increment concentrations of the compound, and the absorbance value of the fully bound form of bovine serum albumin with substance, respectively. Error limit ±2.5%.

### 3.4. DFT and Molecular Modelling Properties

**Figure 8:**
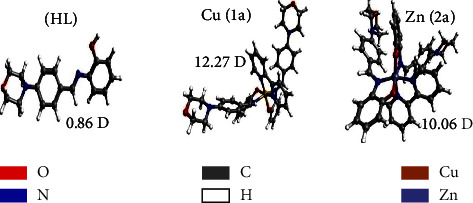
The optimized geometries for the free ligand (**HL**) and its complexes (**1a**-**2a**).

The quantum chemical properties of all substances in the gas phase were examined using DFT calculations. When assessing the electronic structure, stability, and chemical reactivity of substances, quantum chemical characteristics such as HOMO, LUMO, and energy gap (Δ*E*) can be used. The Δ*E* findings of the substances and their biological activity may be connected. The DNA electron cloud largely occupies HOMO in the substance-DNA adduct system, when the LUMO electron cloud is primarily distributed across the intercalative ligands of the metal complex, such as the 2,2′ bipyridine moiety. The overlap of HOMO (DNA) and LUMO (complex) orbitals is enhanced by this type of electron cloud distribution, which results in the intercalation of the complex within DNA [109]. The optimized geometries for all compounds are enclosed in Figure 8. It also indicates the examined dipole moment (DM) next to each compound. Depending on the electronic structure of the central metal, complexes (**1a**-**2a**) are found in either the singlet (**2a**) or doublet ground states (**1a**). In line with the experimental results, all-metal complexes present a distorted octahedral geometry, where the central metal is bound to six coordination sites, including the two phenolic O and iminic N atoms of the ligand, along with the two N atoms of the bipyridine molecule. Dipole moment values suggest that all complexes (**1a**-**2a**) are more polar than that of the free ligand (**HL**). Regarding the complexes, the highest and lowest dipole moments, i.e., 12.27 and 10.06 debye, are assigned to complexes (**1a**) and (**2a**), respectively.

Extensive research has demonstrated that chemical reactivity is mainly dictated by the interaction between the HOMO and LUMO. It is widely admitted that the kinetic stability of a given compound can be correlated with the energetic gap between its FMOs. A rule of thumb is that the larger the gap, the more kinetically stable the system. In the present case, HOMO-LUMO gaps of 3.986, 1.871, and 2.162 eV were predicted for (**HL**) and complexes (**1a**-**2a**), respectively. These values suggest the following order of kinetic stability: Cu(**1a**) < Zn(**2a**) < (**HL**), which further indicates that all synthesized complexes (**1a**-**2a**) have higher reactivity than the ligand and may give rise to better binding profiles with biomolecules.

**Figure 9:**
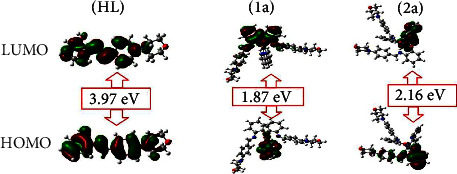
FMO of the free ligand (**HL**) and its complexes (**1a**-**2a**).

**Figure 10:**
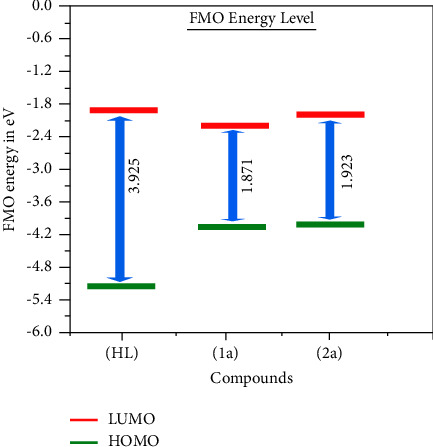
FMO energy level diagram by DFT computation for all compounds.

However, although very handy and widely used to sort molecules in terms of global reactivity trends, the HOMO-LUMO gap does not provide any clue about the reactive regions of the system. This is where local reactivity descriptors come to the rescue. To decipher molecular regions or sites with the highest propensity to withdraw or accept the available density, one can first rely on the electron density distributions in FMOs.  Figure 9 displays the FMOs of all test compounds. As far as the free ligand is concerned, the HOMO covers the whole molecule, while the LUMO does not cover the morpholine ring (except the nitrogen atom). The HOMO of the complex (**1a**) is concentrated on the transition metal and the phenolic rings of the ligand (**HL**), while the LUMO covers the metal and the 2,2′ bipyridine moiety. In the Zn-containing complex (**2a**), FMOs are less delocalized, with the HOMO expanding only over one L unit and the LUMO covering the 2,2′ bipyridine fragment. In particular, the central metal does not contribute to stabilizing these FMOs (Figure 10 and Table 8).

Additional descriptors such as absolute electro negativity (*χ*), absolute hardness (*η*), absolute softness (*σ*), chemical potential *μ*_*i*_, global electrophilicity (*ω*), and additional electronic charge (Δ*N*_max_) are determined from the following equations (38)–(43) [38], and their related findings are summarized in Table 8. Absolute electronegativity (*χ*) indicates a substance is a Lewis acid or a base. While the *χ* finding is high, this is ascribed to the Lewis acid, and while the *χ* finding is low, this is ascribed to the Lewis base [110]. As demonstrated in Table 8, the observed *χ* findings for the complexes (**1a**-**2a**) were put forward that they act as Lewis acids compared to the free ligand. The *η* findings are supported to distinguish the hard and soft molecules. While the *η* finding is high, this is attributed to the hard nature of the molecule. On the other hand, while the *η* finding is low, this is attributed to the soft nature of the molecule. It is also pronounced that the soft nature of substances is more polarisable compared to hard ones [111]. The higher observed findings of *η* for the ligand demonstrate that it is a chemically hard substance compared to the complexes (**1a**-**2a**) (Table 8). Global electrophilicity (*ω*) is denoted as the capability of a substance to absorb electrons from the system. The substances with higher findings of *ω* indicate that they can form several interactions with biomolecules. However, the complexes (**1a**-**2a**) have a larger value of *ω* (5.329–5.624) than their related free ligand (2.535), which supports that the complexes involve strong binding with biomolecules due to numerous interaction modes [112]. It is also concluded that the observed findings of the quantum chemical parameters indicate the chemical reactivity of the complexes (**1a**-**2a**) (Table 8).

Electron volt (eV), Δ*E* (eV) ⟶ energy gap between HOMO and LUMO, HOMO ⟶ highest occupied molecular orbital which is directly related to ionization potential (*I*_*P*_ = −*E*_HOMO_) without negative sign. LUMO ⟶ lowest unoccupied molecular oOrbital, which is directly related to electron affinity (EA = −*E*_LUMO_), and Δ*E* ⟶ the energy gap (*E*_LUMO_ − *E*_HOMO_) (or) Δ*E* = (*I*_*P*_ − EA),

(38) $$\begin{array}{c} \chi =–\left(\frac{E_{HOMO}+{\;E}_{LUMO}\;}{2}\right)\text{,} \end{array}$$

(39) $$\begin{array}{c} \eta =\left(\frac{E_{LUMO}–{\;E}_{HOMO}\;}{2}\right)\text{,} \end{array}$$

(40) $$\begin{array}{c} \sigma =\left(\frac{1\;}{\eta }\right)\text{,} \end{array}$$

(41) $$\begin{array}{c} \omega =\left(\frac{{µ}_{i}^{2}\;}{2\eta }\right)\text{,} \end{array}$$

(42) $$\begin{array}{c} {\Delta N}_{\max}=–\left(\frac{{µ}_{i}\;}{\eta }\right)\text{,} \end{array}$$

where *χ* ⟶ absolute electronegativity, *η* ⟶ absolute (global) hardness, *σ* ⟶ absolute (global) softness, *μ*_*i*_ ⟶ chemical potential, *ω* ⟶ global electrophilicity index, and Δ*N*_max_ ⟶ additional electronic charge. *μ* ⟶ dipole moment (*μ* = *Q* × *r*) is the measure of net molecular polarity, which describes the charge separation in a molecule. It is the product of the charge *Q*, at the end of the molecular dipole, and the distance *r*, between the charges. These parameters are effective in predicting global reactivity trends based on Koopman's theorem.

**Figure 11:**
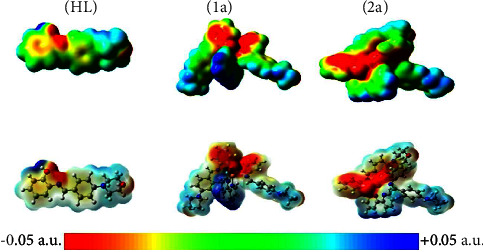
Molecular electrostatic potential (MEP) maps of the free ligand (**HL**) and associated complexes (**1a**-**2a**). Plots generated at the 0.002 isosurface value.

From the visual analysis of FMOs, it comes out that the phenolic rings of (**HL**) and bipyridine units are the most reactive nonmetallic fragments of the complexes (**1a**-**2a**). As such, they are anticipated to participate most actively in a range of intermolecular interactions. Another well-established local reactivity descriptor is the molecular electrostatic potential (MEP). The MEP describes the interaction between the charge distribution of a molecule and a hypothetical positive charge [113]. One of its biggest merits is the capability to effectively recognize the most reactive sites in “hard-hard” interactions, as pioneered by Pearson through the hard-soft acid-base principle [114].  Figure 11 displays MEP maps of (**HL**) and complexes (**1a**-**2a**) calculated at the 0.002 isosurfaces. In this figure, the red and blue colors indicate regions that are expected to be engaged in nucleophilic and electrophilic attacks. Two nucleophilic sites appear on the MEP of the free ligand. The first region surrounds both the phenolic O and imine N atoms and mirrors the presence of the ESP global minimum of −46.6 kcal/mol. The second spot is found in the vicinity of the morpholinic O atom and is identified with a local minimum estimated to be roughly −29.5 kcal/mol. This points out that the first site is more highly reactive than the second and agrees with the fact that the phenolic site is the one that binds to the central metal during the formation of the metal complexes. Conversely, the MEP maps of complexes (**1a**-**2a**) present a negative electrostatic potential all over the phenolate units, whereas the 2,2′ bipyridine fragment carries a positive region enclosing all H atoms fixed opposite to the N atoms. This observation corroborates, at least partly, with the previous analysis of FMOs, highlighting that the phenolate and 2,2′ bipyridine fragments are the preferred binding sites for nucleophilic and electrophilic assaults, respectively. In sum, both the free ligand (**HL**) and complexes (**1a**-**2a**) appear as amphoteric species, able to act as Lewis acids and Lewis bases. Similar systems can be found throughout the literature [115].

**Figure 12:**
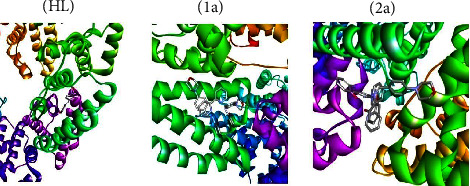
Best docking poses of guest molecules inside the active site of the BSA protein.

The 3D models of the BSA, CT-DNA, and 3CLPro host biomolecules are depicted in Figure S35. The guest molecules, i.e., the free ligand (**HL**) and complexes (**1a**-**2a**), were first docked within the active site of the BSA to assess their binding affinity and figure out the main interactions that ensure the stability of the resulting guest-host complex.  Figure 12 shows the highest docking positions. Calculated binding energies between test compounds and BSA fell in the range from −7.2 to −10.2 kcal·mol^−1^ and suggested the spontaneous formation of the guest-host complex. The binding affinity values were found in the following sequence: −10.2 (**1a**) > −9.5 (**2a**) > −7.2 (**HL**). These binding energies point out that the complexes (**1a**-**2a**) demonstrate superior binding affinity to DNA contrasted to the ligand (**HL**). However, the stability of guest-host complexes, which constitutes the first criterion in the selection of lead compounds in drug discovery pipelines, does not only depend on their inherent reactivity but also on their size, bond length of the metal-ligand system, charge, electron density, polarity, metal ions' influence on the dipole moment, and intermolecular H-bonds, as this determines how easily they will be accommodated inside the active site. Moreover, several noncovalent interactions were found to maintain the guest molecules' interiors at the active site of the BSA (Figure S36). The most noteworthy ones are H-bonding interactions, *π*-*π* stacking, and hydrophobic interactions. For instance, the ligand (**HL**) establishes two conventional H-bonds with BSA, where it behaves as the proton acceptor. In the first interaction, a long of 2.73 Å, the phenolic O atom binds to Phe506, while the second contact involves the morpholinic O atom and the Asn504 amino acid residue. This finding is in line with the local reactivity of the free ligand (**HL**) as predicted by the MEP. Furthermore, the metal complex (**1a**) is also engaged in conventional H-bonds with Lys563, while complex (**2a**) did not form such interactions. The docking of our guest molecules inside 3CLPro was also favourable and showed binding energies in the range from −6.7 to −8.2 kcal·mol^−1^. The most stable guest-host complex was ascribed to complex (**1a**), while (**HL**) formed the least stable one. The binding affinity values between the test compounds and 3CLPro were found to be in the following sequence: −8.2 (**1a**) > −7.9 (**2a**) > (**HL**) − 6.7, which provides support for the enhanced reactivity of the metal complexes (**1a**-**2a**) than ligand (**HL**).

**Figure 13:**
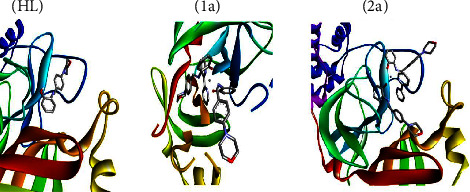
Best docking pose of guest molecules inside the active site of 3CLPro.

**Figure 14:**
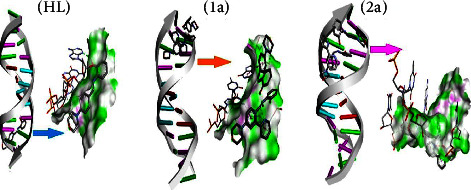
Best binding pose of our guest molecules in the CT-DNA double helix.

Figures 13 and S37 display the best binding poses and the physical interactions that guest molecules establish inside 3CLPro's active site. It put forwards that apart from H-bonds in (HL), *π*-*π* stacking interactions contribute significantly to the stability of guest-host systems. In addition, all-metal complexes (**1a**-**2a**) seem to bind better than the cocrystalized ligand, which frequently performs as an optimistic control [116, 117]. This finding is quite encouraging and should incite further *in vitro* studies to validate the effect of inhibition of our guest molecules on SARS-CoV-2 main protease. Finally, docking calculations on the CT-DNA double helix suggested favourable binding energies for all test compounds were found between −7.7 and −8.6 kcal/mol. In addition, the acquired binding energies for all substances bound with CT-DNA were in the subsequent sequence: −8.6 (**1a**) > −8.2 (**2a**) > −7.7 (**HL**) kcal/mol. As shown in Figure 14, between the two brains of the helix, all substances are effectively sandwiched, where they seem to create both hydrogen bonds (HL) and complexes (**1a**-**2a**) and *π*-*π* stacking (HL) contacts with either of the guanine and cytosine subunits. Therefore, in all cases, these observed binding energies conclude that the metal complex (**1a**) demonstrates higher binding affinity towards DNA, BSA, and 3CLPro proteins, among others.

### 3.5. Assessment of Antioxidant Properties Using UV-Visible Spectral Titration

It is defined as the ability of any substance to put off or reduce the oxidation of the substrate (proteins/lipids/DNA/carbohydrates of living cells) or free radical formation. The biological systems are shielded from the potential adverse effects of excessive oxidation by the oxidizable substrate. As a result, the free radical's energy may be reduced, radical generation suppressed, or the chain propagation of lipid oxidation may be stopped in the initial stages. They also donate hydrogen or electrons to the free radicals, turning them into nontoxic or H_2_O molecules [118–120]. Of late, it has been found that antioxidant studies have attracted special attention among various biological studies due to their vital part in the execution of disorders associated with cancer. The observed percentage of inhibition efficiency for all substances in terms of IC_50_ findings for DPPH, OH, O_2_^−•^, and NO free radical assays was revealed in Figures S38a–S38d and Tables S14–S17.

#### 3.5.1. Assessment of the DPPH Radical Scavenging Property

The colour of an aqueous or methanol solution changes from deep purple to pale yellow when DPPH, a stable chromogen free radical, combines with an antioxidant molecule, which means that DPPH quickly absorbs the hydrogen or electrons from the donor groups. In this case, for the baseline correction, a blank DPPH solution in the absence of a test compound was employed, and 517 nm (*ε* = 8320 M^−1^·cm^−1^) was attained to have a significant absorption maximum. It was found that when test compound concentrations (40–240 *μ*M) increase, the DPPH radical inhibition increases as well. The DPPH radicals are reduced by an antioxidant compound (AH), in which the reduction of electronic absorbance for each compound was vigilantly noted at 517 nm [121]. The capacity to block radicals improves as the sample concentration increases. The assessed percentage of maximum inhibition for all substances was found at 240 *μ*M in the subsequent order: (ascorbic acid) (85.65) > (**1a**) 68.64 > (**2a**) 59.55 > (**HL**) 52.45. The assessed IC_50_ findings of standard ascorbic acid and complex (**1a**) were analyzed at 80 *μ*M and 160 *μ*M, respectively (Figure S38a and Table S14). Moreover, the % of scavenging or maximum inhibition for all substances is estimated with the aid of equation (44) (Table S14).

(43) $$\begin{array}{c} Scavanging\left(\%\right)=\frac{\left[A_{0}-A_{S}\right]}{A_{0}}\times 100 \end{array}$$

#### 3.5.2. Evaluation of Hydroxyl Radical Inhibition

Hydrogen peroxide receives electrons from antioxidant molecules and then neutralizes them into a water molecule. ^●^OH inhibition capability was determined from the % of inhibition for all test substances at 230 nm. The maximum % of inhibition for all samples at 240 *μ*M was observed in the subsequent order: (**1a**) 61.10 > (**2a**) 56.14 > (**HL**) 50.68. The IC_50_ findings for standard ascorbic acid and complex (**1a**) were observed at 160 *μ*M and 200 *μ*M, respectively. Nevertheless, complex (**1a**) demonstrated the best antioxidant potency among others (Figure S38b and Table S15).

#### 3.5.3. Superoxide Scavenging Assay

A vital enzyme catalyst in the body's defence against free radicals, superoxide dismutase (SOD) quickly and efficiently reduces toxicity and cellular damage by exchanging superoxide with water (or) harmless molecules. The % of inhibition for all substances was analyzed at 590 nm. The outcomes were obtained in the subsequent order: (ascorbic acid) 84.85 > (**1a**) 67.17 > (**2a**) 58.18 > (**HL**) 50.42. However, complex (**1a**) revealed the best antioxidant potency among others, and standard ascorbic acid's assessed IC_50_ values were found to be 120 *μ*M and complex (**1a**)'s values to be 200 *μ*M, respectively (Figure S38c and Table S16).

#### 3.5.4. Assessment of Nitric Oxide Inhibition

The diffusible nitric oxide free radical is a crucial chemical mediator, which assists in overcoming diverse chronological human diseases. The NO free radical scavenging potential for all test samples was also carried out at 546 nm. It monitored the alterations in electronic absorption intensity of the nitric oxide radical inhibition outcome concerning the sample concentrations. When the test sample concentration rises, the nitric oxide free inhibition effectiveness also increases. The measured % of nitric oxide radical scavenging capability for all samples at 240 *μ*M was obtained in the subsequent order (ascorbic acid) 72.73 > (**1a**) 65.47 > (**2a**) 58.43 > (**HL**) 51.62. However, complex (**1a**) showed superior antioxidant efficacy among others, and standard ascorbic acid's IC_50_ findings for ascorbic acid and complex (**1a**) were observed to be 160 M and 200 M, respectively (Figure S38d and Table S17).

### 3.6. Evaluation of Antimicrobial Properties

**Figure 15:**
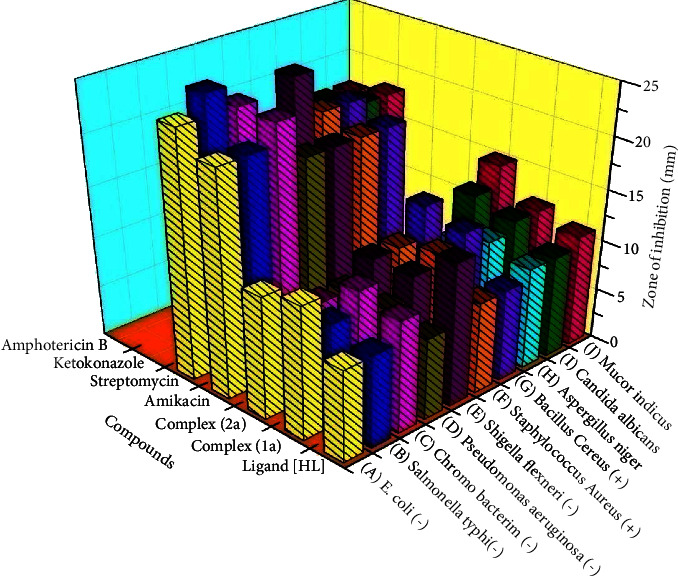
Agar disc diffusion technique histogram comparing the antibacterial effects of all substances.

The current research has a curious focus on the *in vitro* antimicrobial properties of biological systems because these studies are crucial for developing effective antibacterial and antifungal medications. The obtained clear inhibition zone (mm) values towards various bacterial and fungal species for the test samples were revealed in Figure 15, and the evaluated findings were summarized in Table 9. The outcomes of the microbial activities revealed that the metal chelates demonstrated greater efficacy compared to ligand (**HL**) against the chosen bacterial and fungal pathogens owing to the improved lipophilicity of the substances under similar circumstances, and they accelerate the cell wall breaking down during the biosynthesis of the microorganisms' enzymes as well as damage the normal cell processes due to increasing the permeability of cells into lipid membranes [122]. The obtained results suggest that complexes (**1a**-**2b**) demonstrate significantly greater antimicrobial properties than the free ligand (**HL**) against the chosen microorganism, and they are contrasted with standard drugs like *amikacin* and *streptomycin* for bacteria, and *ketokonazole* and *amphotericin B* for fungal species. It can also be spelled out based on the chelation theory proposed by Overtone and Tweedy [123]. Chelation theory points out that the partial exchanging of the positive charge of the metal center with donor moieties, and overlap of the ligand orbitals will reduce the greater degree of the metal ion's polarity, which ultimately rises the delocalization of *π* and *d* electrons over the full chelated ring system. By raising the size of the metal ion due to the retarding of polarization, chelation may also enhance the complexes' lipophilic characteristics, which further stimulate the lipid membrane permeability and break down the bacteria' enzymes responsible for cell wall formation, therefore slowing down the regular cell processes. By preventing the production of cell walls/proteins/DNA, including by obstructing folate metabolism and the cytoplasmic membrane, antimicrobial drugs frequently either fully eliminate microbes or only prevent their cell growth. Additionally, the samples' mode of action may be employed in disrupting the cell's respiration process by forming an H-bond during the morpholine-fused iminic group coordinated with the active metal center of its components, inhibiting proliferation of the cell. It is also revealed that the enhanced antibacterial activity could be attributed to changes in pharmacological kinetics, conductivity, steric, electronic, solubility, and metal-ligand bond length. The difference in the antimicrobial efficacy of some of the compounds towards various microorganisms influences on the impermeability of the cells of the germs or the diversity of ribosomes of the microbes [124]. The % of the inhibition of all substances is estimated from (45) (Table 9).

(*A*, *B*, *C*, *D*, and *E* are represented as gram negative bacteria species *Escherichia coli, Salmonella enteric serovar typhi, Salmonella enteric serovar typhi, Pseudomonas aeruginosa, and Shigella flexneri, respectively*. *F* & *G* are denoted as gram positive bacteria species *Staphylococcus aureus and Bacillus cereus. H*, *I,* and *J* are represented as fungal strains *Aspergillus niger, Candida albicans, and Mucor indicus. Standard drugs for bacterial strains: Amikacin* & *Streptomycin Standard drugs for fungal strains: Ketoconazole and Amphotericin B*. [*Control* (*DMSO*) = 6 mm].

(44) $$\begin{array}{c} Inhibition\;\%=\left[\frac{\left(T–C\right)}{T}\right]\times 100\text{,} \end{array}$$

where *T* and *C* are represented as the diameter of microbial growth of the sample plates and the control plate (6 mm), respectively. Error limits ±2.5–5.0% (*P* ≤ 0.05).

### 3.7. Evaluation of Anticancer Properties

**Figure 16:**
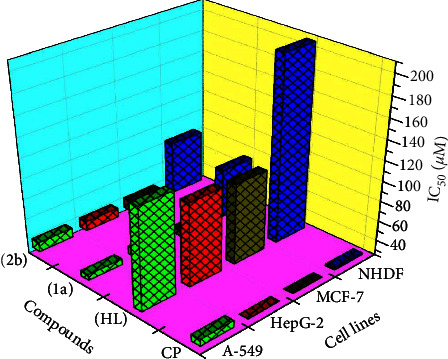
The evaluation of the anticancer properties of ligand (**HL**) and its complexes (**1a**-**2a**) against cancer cell lines and normal cell lines in comparison to the standard medication cisplatin (**CP**). Error limits ±2.5–5.0% (*P* ≤ 0.05).

Cellular viability or metabolic properties can be measured using the MTT assay, which is a powerful and credible method for anticancer properties. The anticancer efficacy of all test compounds was investigated by the MTT assay against A549, HepG2, MCF-7, and NHDF cell lines [125]. As per the colorimetric approach, the IC_50_ findings of all test substances were evaluated as the % of cell viability/growth inhibition [126]. Even though the complexes show superior activity than ligand (HL) against some cancer cell lines, the NHDF cell line is only mildly perturbed by cisplatin. Nevertheless, complex (**1a**) has the highest cytotoxic potential among others [127]. The acquired findings were in the subsequent sequence as follows: (cisplatin) > (**1a**) > (**2a**) > (**HL**) (Figure 16 and Table 10). The cytotoxic effectiveness is dependent upon the DNA-binding modalities, the structure-activity relationship, as well as drug concentrations and incubation period exposure [128]. In addition, it is suggested that these complexes consist of morpholine fused primary aromatic and secondary 2,2′-bipyridine planar systems connected with a metal center, which facilitate their simple insert within the base pairs of DNA. As per Tweedy's chelation theory, charge equilibration happens as a result of coordination between the ligands and metal ions, which minimises the polarity of the metal ions and causes the capability of the test complexes to pass *via* the cell membrane lipid layer. Thus, it put off the synthesis of cell wall/protein/nucleic acids. The measured % of the inhibition results of growth for these compounds is summarized in Table 10. Additionally, the results of DNA-binding tests using these complexes, including gel electrophoresis, UV-visible spectral titration, hydrodynamic, emission, and CV findings, were in good agreement with the findings of cytotoxicity [129]. The expressions (46) and (47) were supported to measure the % of growth inhibition and cell viability. The complex (**1a**) has been proven to have greater biological efficiency, among others, which also based on the Lewis acid character, solubility, conductivity, bond length of metal-ligand, charge, electron density, dipole moment, intermolecular hydrogen bonding, and proton transfer equilibrium, etc. These significant elements might also contribute to the increased biological activity. The Cu^+^ ion is unique among the transition metals owing to its size, *d*^10^ electronic configuration, softness, and flexible characteristics of the distorted coordination geometry. The effect of the Cu^+^ is a reduced form of the complexes' conformation, symmetry, and functionality that results in increased biological efficacy. Depending on the ligand donor selected, Cu^+^ can also be reduced as an intervening molecule. Despite both acting as *d*^10^ ions, Cu^+^ is softer and more flexible than Zn^2+^ ions. Hence, the copper complex may promote DNA damage while inhibiting its repair, producing a double-edged effect. According to numerous research findings, *via* cell apoptosis or enzyme inhibition, the great anticancer efficacy of copper complexes has been proven.

Average IC_50_ values from at least three independent experiments for drug concentration *μ*g/mL of 50% cell death following 72 hours exposure. A549, HepG2 MCF-7, and NHDF are represented as human lung cancer cell line, liver cancer cell line, breast cancer cell line, and normal human dermal fibroblasts cell line.

(45) $$\begin{array}{c} \%\;Cytotoxiciy=\left[1-\left(\frac{\;Mean\;absorbance\;of\;Sample\;at\;595\;nm}{Mean\;absorbance\;of\;Controlat\;595\;nm\;}\right)\right]\times 100\text{,} \end{array}$$

(46) $$\begin{array}{c} Cellviability\left(\%\right)=\left[100-Cytotoxiciy\left(\%\right)\right]\text{.} \end{array}$$

Error limits ±2.5–5.0% (*P* ≤ 0.05).

## 4. Conclusion

All compounds are treated with diverse analytical, spectral, and X-ray diffraction analyses. The examined results of the complexes (**1a**-**2a**) proposed an octahedral geometry. The gel electrophoresis results showed that complex (**1a**) revealed excellent metallo-nuclease efficacy in a H_2_O_2_ environment. The observed DNA-binding properties of all compounds by spectro-electro-hydrodynamic and fluorometric titrations disclose that complexes (**1a**-**2a**) could bind to deoxyribonucleic acid *via* intercalation. The observed BSA-binding constants of all samples recommend that the complexes could interact with BSA in static mode, which is further supported by FRET detection. Complex (**1a)** also possessed the best DNA-/BSA-binding affinities compared to others. The electronic configuration data of these substances were attained from DFT computations and their molecular docking studies on the interacting affinity of these substances with DNA/BSA/SARS-CoV-2. The findings demonstrated that the metal complexes bind spontaneously inside the active sites of these biomolecules. Additionally, the enhanced reactivity of complexes with respect to the free ligand is well accounted for in the context of the FMO theory. The theoretical measurements for all substances were reported to be in excellent accord with the experimental findings. The antimicrobial properties exposed that the metal complexes have highly significant inhibition potency than the ligand (**HL**). The scavenging properties put forward by complex (**1a**) stood out as having a greater potential to scavenge radicals than other substances. The observed *in vitro* anticancer characteristics' findings for all substances and cisplatin (**CP**) revealed that complex (**1a**) has revealed the best cytotoxic efficiency among others, and the faintly influenced normal cell was found to be less compared to cisplatin. In the future, complex (**1a**) might function as a brand-new class of anticancer agent.

## Figures and Tables

**Scheme 1:**
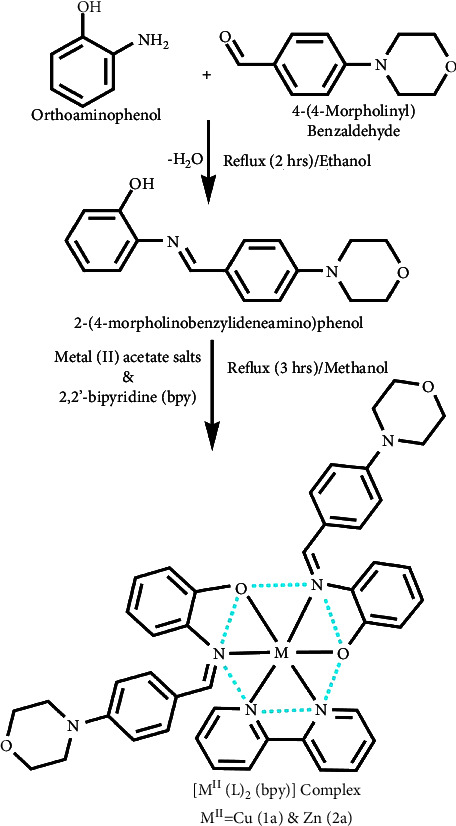
The suggested geometry of complexes (**1a**-**2a**) **[M**^**II**^**(L)**_**2**_**(bpy)]**.

**Table 1:** UV-visible spectral DNA-binding parameters for all substances.

Compounds	*λ *_max_ (nm)	Δ*λ* nm (% H)	*K *_*b*_ × 10^4^ M^−1^			∆*G*_*b*_° (kJ·mol^−1^)		
	Free (bound)		WS-I	BH-I	SK-I	WS-I	BH-I	SK-I
			(WS-II)	(BH-II)	(SK-II)	(WS-II)	(BH-II)	(SK-II)
(**HL**)	336 (340)	04 (37.13)	1.5169 (1.5480)	0.6000 (1.9515)	0.8775 (1.7216)	−23.85 (−23.90)	−21.55 (−24.48)	−22.50 (−24.18)
(**1a**)	336 (343)	07 (52.18)	1.9000 (2.1886)	3.6777 (2.4500)	1.7093 (2.6303)	−24.114 (−24.76)	−26.04 (−25.04)	−24.15 (−25.22)
(**2a**)	337 (342)	05 (43.15)	1.8287 (1.7209)	2.2115 (2.2605)	1.4286 (2.0118)	−24.315 (−24.16)	−24.79 (−24.84)	−23.70 (−24.55)

**Table 2:** UV-vis absorption spectra with biothermodynamic binding properties for all substances with CT-DNA.

Compounds	*T *_*m*_ °C (K)	Δ*T*_*m*_ °C	Binding constant	Binding constant	ΔH°	ΔS°	ΔG°
			*K *_*r*_ at 298 K (M^–1^)	*K *_*m*_ at *T*_*m*_ K (M^–1^)	(kcal mol^–1^)	(cal mol^–1^)	(kcal mol^–1^)
(**HL**)	74 (347)	6	1.5169 × 10^4^	1.4625 × 10^3^	–9.8083	–13.7851	–5.0249
(**1a**)	80 (353)	12	1.9000 × 10^4^	2.3722 × 10^3^	–7.9071	–6.9577	–5.4511
(**2a**)	78 (351)	10	1.8287 × 10^4^	2.1862 × 10^3^	–8.3292	–8.4501	–5.3632

**Table 3:** Relative specific viscosity *versus* [complex]/[DNA].

Compounds	Binding ratio (*R*) = [complex]/[DNA]						
	0.2	0.4	0.6	0.8	1.0	Slope	*R * ^2^
	Relative specific viscosity (*η/η*_0_)^1/3^						
**EB** (control)	1.01	1.35	1.63	1.82	1.99	1.215	0.92002
(**HL**)	0.61	0.67	0.75	0.85	1.01	0.490	0.97018
(**1a**)	0.88	1.11	1.22	1.38	1.66	0.915	0.90375
(**2a**)	0.74	0.81	0.87	1.03	1.26	0.630	0.96043

**Table 4:** Determination of *K*_*b*_ and *n* values for all substances with DNA/BSA at pH of 7.4 using spectrofluorometer.

Compounds	DNA-/BSA-binding constants									
	SV methods for determining DNA-binding characteristics (SV methods for determining BSA-binding characteristics)^*∗*^						LWB method *K*_LB_ × 10^4^ M^−1^	Scatchard analysis		*K *_app_ × 10^7^ M^−1^
	Method I		Method II							
	*K *_*q*_ × 10^12^ M^−1^s^−1^	*K *_SV_ × 10^4^ M^−1^	*K *_ass_ × 10^4^ M^−1^	*n*	∆*G*_*b*_° (kJM^−1^)	P		*K *_SA_ × 10^4^ M^−1^	*n*	
(**HL**)	1.1636 (2.639)	1.1636 (2.639)	0.9606 (1.062)	0.973 (0.926)	−22.720 (−23.0)	0.0900 (0.464)	0.6985	2.3194	1.096	0.5829
(**1a**)	5.2474 (8.691)	5.2474 (8.691)	5.0564 (8.734)	1.168 (1.059)	−26.835 (−28.2)	0.2000 (0.252)	1.8846	3.3151	1.033	0.9894
(**2a**)	3.2694 (4.805)	3.2694 (4.805)	1.7368 (5.291)	1.055 (1.041)	−24.187 (−26.9)	0.1832 (0.348)	1.5630	3.0190	0.971	0.6569

**Table 5:** FRET parameters for donor (BSA)—acceptor (compound) systems.

Compounds	*J* × 10^−14^ (LM^−1^·cm^3^)	*R *_0_ (nm)	*E*	*r* (nm)	*k *_ET_ (J/s)	*B* (M^−1^·cm^−1^)
(**HL**)	0.8215	2.4400	0.3462	2.7127	5.2957	5339.79
(**1a**)	0.7607	2.4090	0.5769	2.2877	13.6340	5187.78
(**2a**)	0.5425	2.2770	0.5615	2.1851	10.4206	4642.53

**Table 6:** Redox potential patterns for the interaction of DNA with ligand (**HL**) and its complexes (**1a**-**2a**).

Compounds	Δ*E*_*P*_ (V)	*E*° (or) *E*_1/2_ (V)	*K*_[red]_/*K*_[oxi]_	*I*_pa_/*I*_pc_	*D*_0_ × 10^−5^ cm^2^·s^−1^	*K *_*b*_ × 10^4^ M^−1^ (methods)			*S* (bp)
	Free (bound)	Free (bound)	Found (I) (Calcd)	Free (bound)	Free (bound)	I red (oxi)	II	III	
(**HL**)	0.7420 (0.8890)	0.3490 (0.3680)	0.7214 (2.0964)	1.4295 (1.3424)	2.8570 (2.5809)	0.3809 (0.528)	0.2443	0.4837	0.452
(**1a**)	0.4352 (0.2807)	0.7054 (0.7398)	0.8628 (3.76)	1.2653 (0.2031)	4.9799 (4.3266)	2.1854 (2.533)	7.0773	2.1567	0.123
(**2a**)	0.4819 (0.2965)	0.5406 (0.5719)	0.8268 (3.34)	1.8999 (0.5000)	3.9665 (3.6257)	2.0348 (2.461)	2.7693	1.8486	0.270

**Table 7:** UV-visible titration parameters for all substances bound to bovine serum albumin.

Compounds	*λ *_max_ (nm)		Δ*λ* (nm)	Chromism (% H)	Binding constant *K*_app_ × 10^4^ M^−1^ by BH method	∆*G*_*b*_° (kJ·mol^−1^)
	Free	Bound				
(**HL**)	278	276	02	47.79	0.7556	−22.1246
(**1a**)	280	275	05	65.62	8.1059	−28.0038
(**2a**)	280	276	04	55.04	4.4355	−26.5010

**Table 8:** Quantum chemical parameters (eV) as well as FMO energy gap and dipole moment values of free ligand (**HL**) and metal complexes (**1a**-**2a**).

Compounds	HOMO (eV)	LUMO (eV)	H-L gap (eV)	*χ*	*η*	*σ*	*μ *_*i*_	*ω*	Δ*N*_max_	*μ* (debye)
(**HL**)	−5.177	−1.192	3.985	3.155	1.963	0.510	−3.155	2.535	1.607	0.86
(**1a**)	−4.178	−2.308	1.871	3.243	0.935	1.070	−3.243	5.624	3.468	12.27
(**2a**)	−4.163	−2.240	1.923	3.202	0.962	1.040	−3.202	5.329	3.328	10.06

**Table 9:** Investigation of all substances' antimicrobial properties (measured as the diameter of the clear zone inhibition in mm) (inhibition %).

Compounds	Antibacterial activity							Antifungal activity		
	*A*	*B*	*C*	*D*	*E*	*F*	*G*	*H*	*I*	*J*
Ligand (**HL**)	09 (33)	09 (33)	11 (45)	08 (25)	14 (57)	09 (33)	09 (33)	10 (40)	10 (40)	11 (45)
Complex (**1a**)	13 (54)	10 (40)	12 (50)	09 (33)	11 (45)	12 (50)	13 (54)	11 (45)	12 (50)	12 (50)
Complex (**2a**)	12 (50)	09 (33)	09 (33)	09 (33)	11 (45)	11 (45)	14 (57)	09 (33)	13 (54)	15 (60)
*Amikacin*	22 (73)	22 (73)	24 (75)	20 (70)	20 (70)	20 (70)	20 (70)	—	—	—
*Streptomycin*	24 (75)	26 (77)	24 (75)	21 (71)	25 (76)	21 (71)	21 (71)	—	—	—
*Ketoconazole*	—	—	—	—	—	—	—	16 (63)	18 (67)	18 (67)
*Amphotericin B*	—	—	—	—	—	—	—	15 (60)	17 (65)	17 (65)

**Table 10:** The evaluation of the anticancer properties of ligand (**HL**) and its complexes (**1a**-**2a**) against cancer cell lines and normal cell lines.

Compounds	IC_50_ (*μ*M)			
	A549	HepG2	MCF-7	NHDF
Cisplatin	31.9 ± 1.6	22.9 ± 1.1	20.2 ± 1.0	26.9 ± 1.3
(**HL**)	126.4 ± 6.3	108.4 ± 5.4	105.2 ± 5.3	208.6 ± 10.4
(**1a**)	30.8 ± 1.5	33.1 ± 1.7	32.1 ± 1.6	73.6 ± 3.7
(**2a**)	34.3 ± 1.7	34.6 ± 1.7	35.6 ± 1.8	78.4 ± 3.9

## Data Availability

The spectro-electro-hydrodynamic and fluorometric titrations and comparison with theoretical measurements for DNA/BSA/SARS-CoV-2 biomolecules, radical scavenging, and cytotoxic properties are included within the article, and the physicochemical characteristics of the ligand and complexes to support the findings of this study can be referred to at https://doi.org/10.1039/c8ra09218d and https://doi.org/10.1016/j.jinorgbio.2022.111953.

## Supplementary Materials

Supplementary Materials Tables (S1–S17) and Figures (S1–S38) were encapsulated in the electronic supplementary information file.
