# Supplementary material for: Comprehensive Assessment of Biomolecular Interactions of Morpholine-Based Mixed Ligand Cu(II) and Zn(II) Complexes of 2,2′-Bipyridine as Potential Anticancer and SARS-CoV-2 Agents: A Synergistic Experimental and Structure-Based Virtual Screening
Source: Bioinorg Chem Appl. 2022 Dec 12;2022:6987806. doi: 10.1155/2022/6987806 (PMC9763021; doi:10.1155/2022/6987806)
Supplement: Supplementary Materials — Tables (S1–S17) and Figures (S1–S38) were encapsulated in the electronic supplementary information file. [file 6987806.f1.docx]

**Electronic Supplementary Information File**

**Comprehensive assessments of biomolecular interactions of morpholine-based mixed ligand Cu(II) and Zn(II) complexes of 2,2'-bipyridine as potential anticancer and SARS-CoV-2 agents – A synergistic experimental and structure-based virtual screening**

**Karunganathan Sakthikumar ^1^, Bienfait Kabuyaya Isamura^1, 3^ and Rui Werner Maçedo Krause ^1, 2^ ***

*^1^ Department of Chemistry, Center for Chemico- and Biomedicinal Research (CCBR), Faculty of Science, Rhodes University, Grahamstown 6140, Eastern Cape, South Africa*

*^2^Center for Chemico- and Biomedicinal Research (CCBR), Faculty of Science, Rhodes University, Grahamstown 6140,* *Eastern Cape, South Africa*

*^3^ Department of Chemistry, The University of Manchester, Manchester M13 9PL, Great Britain*

***** *Correspondence:* [*r.krause@ru.ac.za*](mailto:r.krause@ru.ac.za)

***** *Correspondence should be addressed to Rui Werner Maçedo Krause:*

| ***Prof. Rui WM Krause,***  *Professor, Organic & Medicinal Chemistry,*  *Department of Chemistry, Faculty of Science, Rhodes University, Grahamstown 6140,*  *Eastern Cape, South Africa.*  *Email:* [*r.krause@ru.ac.za*](mailto:r.krause@ru.ac.za),  *Phone: +27 741622674;* *+27 46 603 7030* |
| --- |

**3a. Results and Discussions**

***3.1. Characterization of molecular Structure***

| **Table S1** Analytical and physical data of ligand (**HL**) and its complexes (**1a**–**2a**). | | | | | | | | |
| --- | --- | --- | --- | --- | --- | --- | --- | --- |
| **Compounds**  **(EF & FW)** | **Colour** | **Yield**  **(%)** | **M.P**  **(^°^C)** | **Found (Calcd) (%)** | | | | **∧_m_** |
|  |  |  |  | **C** | **H** | **N** | **M** |  |
| (**HL**) (C_17_H_18_N_2_O_2_)  (282.33) Ligand | Dark yellow | 87.52 | 148 | 72.55  (72.25) | 06.53  (06.37) | 10.04  (09.91) | ---- | 16.12 |
| (**1a**) (C_44_H_42_N_6_O_4_)Cu  (781.68) [Cu(L)_2_(bpy)] | Bluish  Green | 82.60 | 142 | 67.68  (67.55) | 05.45  (05.37) | 10.70  (10.75) | 08.22  (08.13) | 51.84 |
| (**2a)** (C_44_H_42_N_6_O_4_)Zn  (783.55) [Zn(L)_2_(bpy)] | Reddish  Brown | 78.91 | 181 | 67.48  (67.39) | 05.41  (05.36) | 10.90  (10.72) | 08.43  08.34 | 36.72 |
| EF → Empirical Formula, FW→ Formula Weight, M.P→ Melting Point, ∧_m_ → Molar Conductance | | | | | | | | |

***3.1.1. Preparation of ligand (HL)***

2-(4-morpholinobenzylideneamino)phenol ligand (**HL**) was synthesized by stirring an equal molar quantity (0.01 M) of ortho amino phenol and 4-(4-morpholinyl) benzaldehyde (0.01 M) in ethanol (30 ml) and the dark yellow precipitate was attained after refluxing for three hrs and in a water bath, the solution's volume was decreased to one-third and cooled at 25 °C. In vacuum desiccators over anhydrous CaCl_2_, the gathered pure dark yellow solid ligand was progressively dried at ambient temperature. It was discovered that the isolated ligand's (**HL**) yield was 87.52 percentages (**Scheme 1**).

***3.1.2. Preparation of mixed ligand complexes (1a***–**2*a)***

A solution of free ligand (0.002 M) in methanol (40 mL) was gradually mixed to a solution of metal(II) acetate salts (0.001 M) [Cu^II^(OAc)_2_.H_2_O, and Zn^II^(OAc)_2_.2H_2_O] in 30 mL of distilled and after stirring for 30 minutes, the hot solution mixture was gradually mixed into the methanolic solution of 2,2'-bipyridine (bpy) (0.001 M). The obtained solution was further refluxed for three hours. The obtained precipitate was further purified using the recrystallization method in the presence of methanol-petroleum ether mixture. Moisture and other solvents were removed by anhydrous CaCl_2_ in the vacuum desiccators. Similar techniques were used to synthesize mixed ligand complexes (**1a**–**2a**), and the yield was obtained to be 78.91–82.60 % percentages (**Scheme 1**) and they were summarized in **Table S1**.

***3.1.3. Analytical Methods: Elemental Analysis and Molar Conductance Studies***

The free ligand (**HL**) and its complexes (**1a**–2**a**) were noted to be deeply coloured, which are soluble in CH_3_OH, C_2_H_5_OH, CHCl_3_ and DMSO. The molar conductance (∧_m_) of these complexes (**1a**–2**a**) was observed in the range of 36.72 – 51.84 Ohm^-1^ cm^2^ mol^-1^, which was higher than that of free ligand (16.12 Ohm^-1^cm^2^ mol^-1^). The molar conductance results are attributed that they are non-electrolytes. The analytical information data and physical properties of all test compounds are enclosed in **Table S1**.

***3.1.4. Mass Spectra (ESI/LC-MS)***

Free ligand demonstrates the molecular ion peak at *m/z* 282.3 (M) consequent to [C_17_H_18_N_2_O_2_]^+^ and other fragmented molecular ion peaks are observed at 244.3 (M+1) [C_14_H_15_N_2_O_2_]^+^, 216.2 (M+2) [C_13_H_14_N_2_O]^+^, 189.3 (M+1) [C_11_H_12_N_2_O]^+^, 176.3 (M+2) [C_11_H_12_NO]^+^, 162.2 (M+1) [C_10_H_11_NO]^+^ and 118.1 (M+1) [C_8_H_7_N]^+^ (**Fig. S1**). Complex (**1a**) (**Fig. S2**) molecular ion peak at *m/z* 781.5 (M) consequent to [C_44_H_42_N_6_O_4_Cu]^+^ and other fragmented molecular ion peaks are observed at 674.1 (M) [C_38_H_37_N_5_O_3_Cu]^+^, 452.1 (M) [C_24_H_27_N_3_O_2_Cu]^+^, 324.2 (M+2) [C_15_H_18_N_2_O_2_Cu]^+^ and 283.0 (M+1) [C_17_H_18_N_2_O_2_]^+^. Complex (**2a**) (**Fig. S3**) molecular ion peak at *m/z* 784.2 (M+1) relevant to [C_44_H_42_N_6_O_4_Zn]^+^ and other fragmented molecular ion peaks are found at 677.4 (M+1) [C_38_H_37_N_5_O_3_Zn]^+^, 502.2 (M-1) [C_27_H_26_N_4_O_2_Zn]^+^, 459.0 (M) [C_25_H_22_N_4_OZn]^+^, 324.3 (M+1) [C_15_H_18_N_2_O_2_Zn]^+^ and 283.0 (M+1) [C_17_H_18_N_2_O_2_]^+^. Also, the generation of other peaks are accordance with the fragmented molecular ion of free ligand. The observed similar fragmented molecular ion peaks for other complexes were good agreed with molecular structures.

| **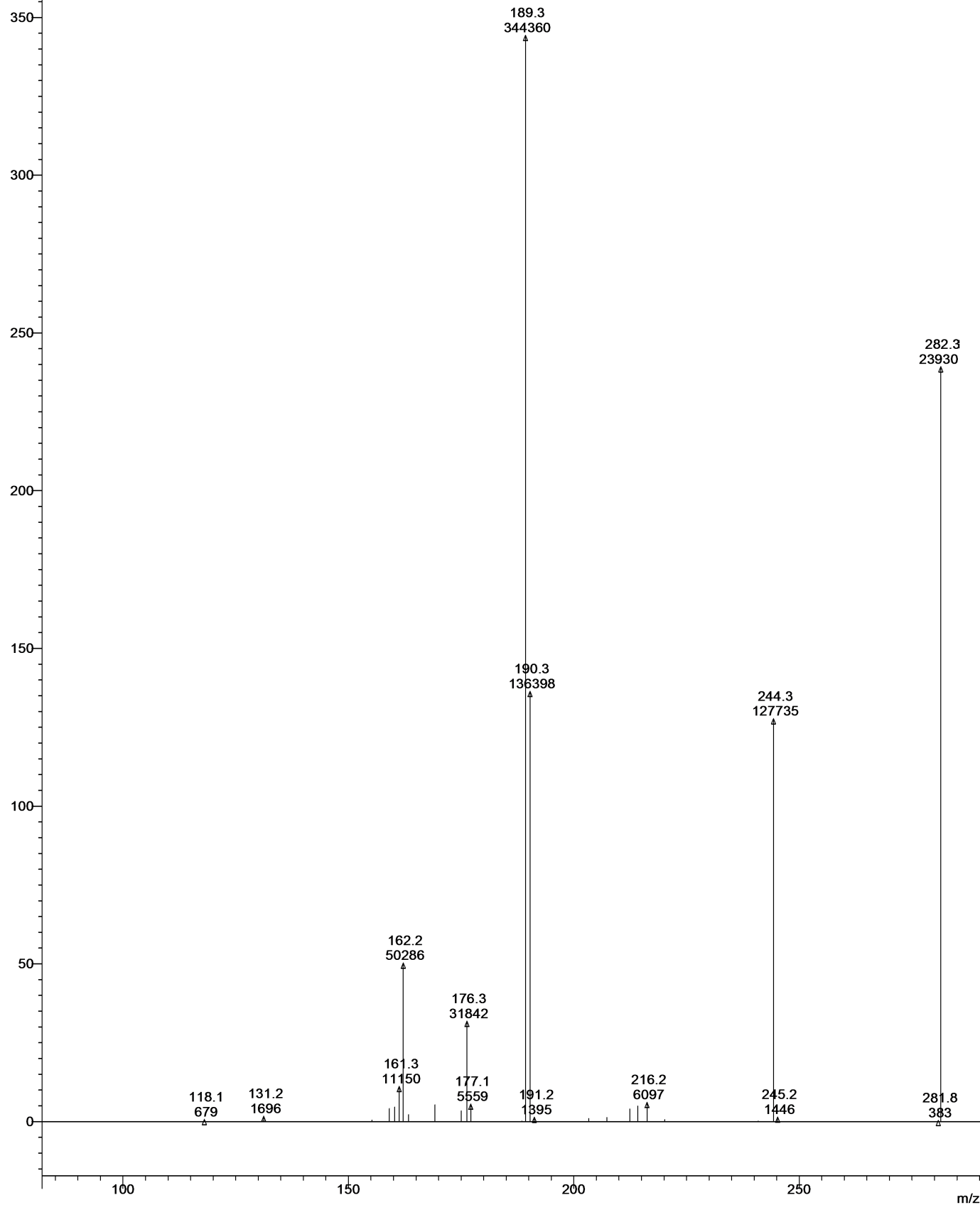**  **Fig. S1** LC-MS mass spectrum of ligand (**HL**). |
| --- |

| 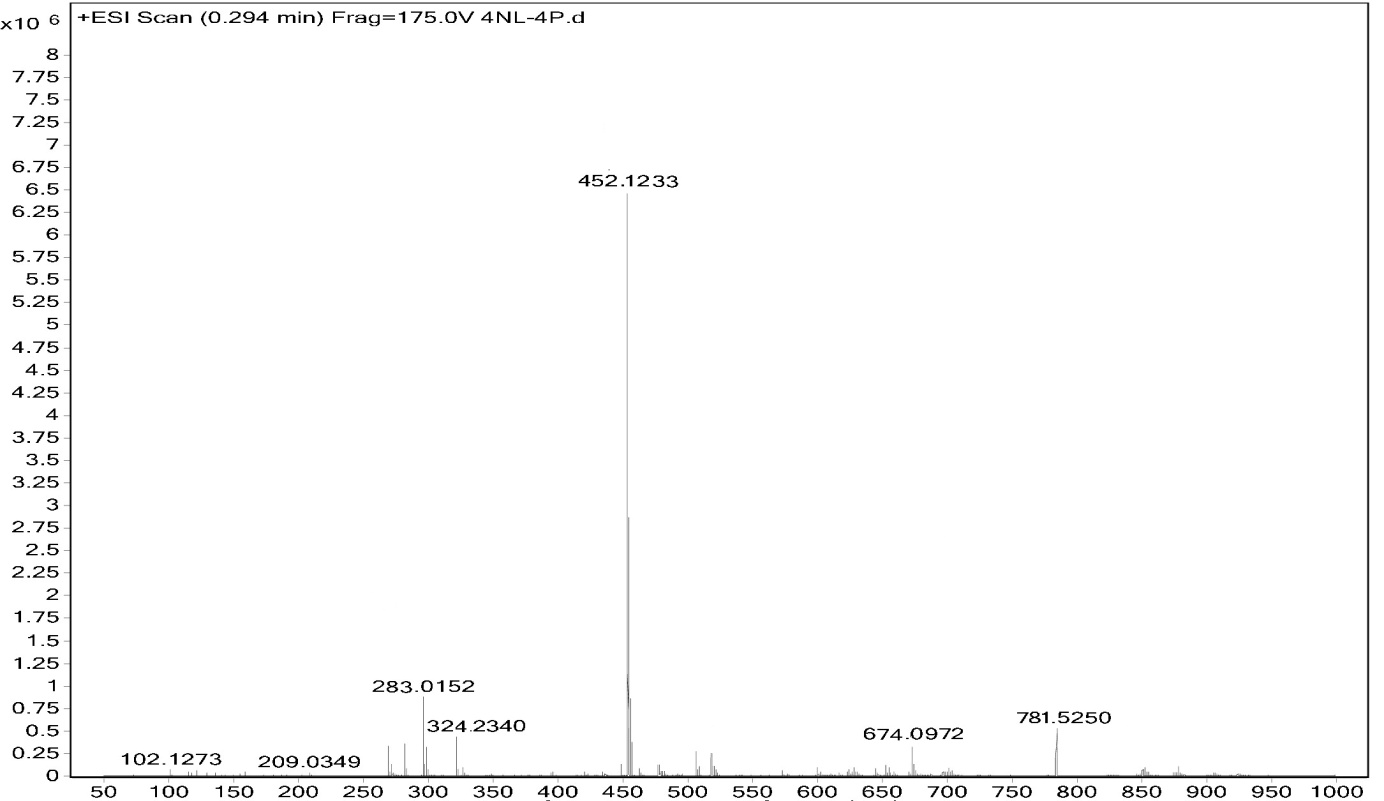  **Fig. S2** ESI mass spectrum of complex (**1a**) |
| --- |

| 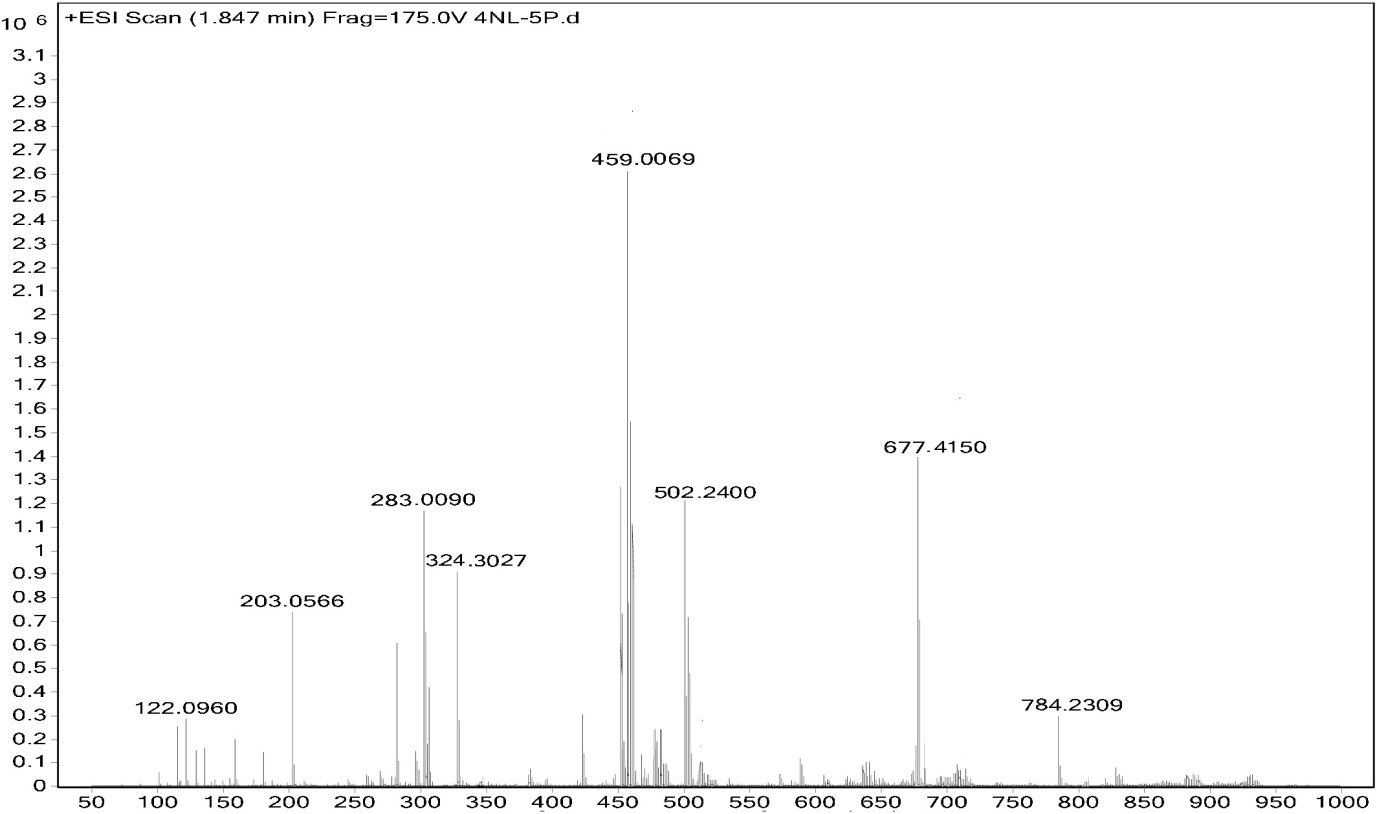  **Fig. S3** ESI mass spectrum of complex (**2a**) |
| --- |

***3.1.5. ^1^H NMR***

The ^1^H NMR spectral properties were executed in presence of CDCl_3_ solvent using Si(CH_3_)_4_ as an internal standard. The ligand (**HL**) and its complex (**2a**) demonstrate the following signals in **Figs. S4 & S5** and the observed results were also listed in **Table S2**. δ findings of ligand (**HL**): aromatic protons (m, 8H) at 6.45 – 7.65 ppm; iminic proton (-HC=N-) (s,1H) at 8.58 ppm; morpholinic-OCH_2_ protons (t, 4H) at 3.85 ppm; morpholinic-N-CH_2_ (t, 4H) at 3.30 ppm; phenolic-OH proton (s, 1H) at 5.10 ppm. Complexes (**2a**): aromatic protons (m, 8H / m, 11H) at 6.85 – 7.42 ppm; iminic proton (-HC=N-) (s, 1H) at 9.01 ppm; bpy-CH=N protons (s, 1H) at 9.62 ppm. The disappeared singlet peak in the range of 13–14 or 5–6 ppm in the complex (**2a**) designated the–OH proton loss owing to complexation. Free 2,2'-bipyridine exhibited four sets of resonances, centered at δ 7.32 ppm (H5, ddd), at 7.83 ppm (H4, ddd), 8.41 ppm (H3, d) and 8.70 ppm (H6, d). In the complex (**2a**), the chemical shifts for the bpy moiety protons are shifted to downfield at 9.62 ppm due to complexation, which further points out that the deshielding effect of the electron atmosphere in the bipyridine ring reduces after complexation of the nitrogen atoms in bpy with the Zn^2+^ ion. Azomethine and 2,2'-bipyridine iminic proton signals were significantly raised into deshielding due to there have involved in complexation. Furthermore, the strong singlet peak for CDCl_3_ solvent was observed at 7.26 ppm in both ligand (**HL**) and complex (**2a**) and other signals are no appreciable changes in the complex (**2a**).

| **Table**  **S2** ^1^H NMR spectral data of the ligand (**HL**) and its mixed ligand complex (**2a**). | | | | | | |
| --- | --- | --- | --- | --- | --- | --- |
| **Compounds**  **(ppm)** | **Ar-protons**  **(m, 8H) /**  **(m, 11H)** | **HC=N-**  **(s,1H)** | **Morp.**  **O-CH_2_-**  **(t, 4H)** | **Morp.**  **N-CH_2_-**  **(t, 4H)** | **Phenolic**  **-OH (s,1H)** | **bpy-CH=N-**  **(d,**  **H6)** |
| (**HL**) | 6.45 - 7.65 | 8.58 | 3.85 | 3.30 | 5.10 | - |
| **(2a)** | 6.85 - 7.42 | 9.01 | 3.83 | 3.26 | - | 9.62 |
| HC=N- → Iminic proton, Morp. O-CH_2_-, N-CH_2_- → Morpholinic protons, bpy-CH=N- → 2,2'-bipyridine protons.. | | | | | | |

| 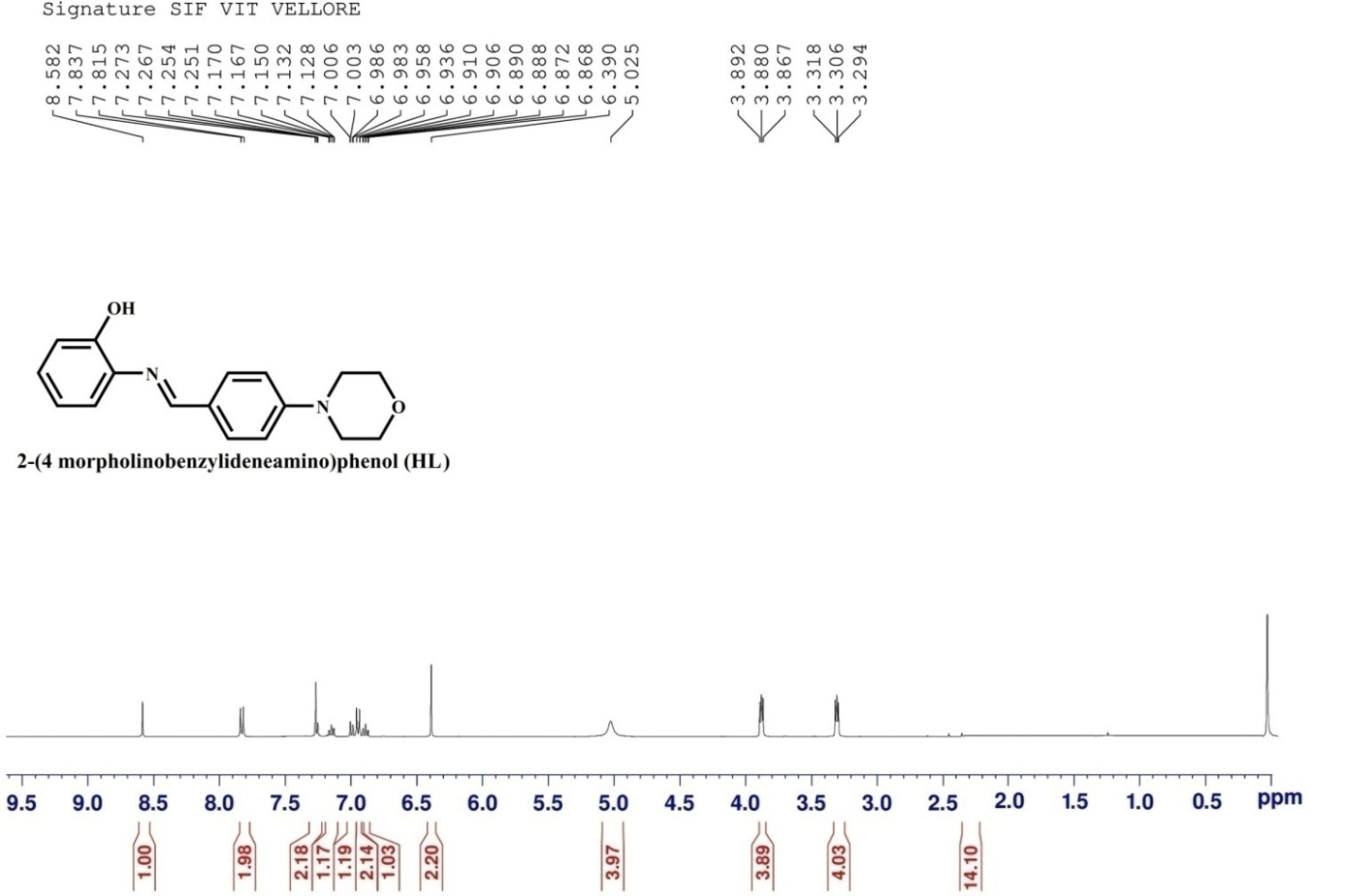  **Fig. S4** ^1^H NMR spectra of ligand (**HL**). |
| --- |

| 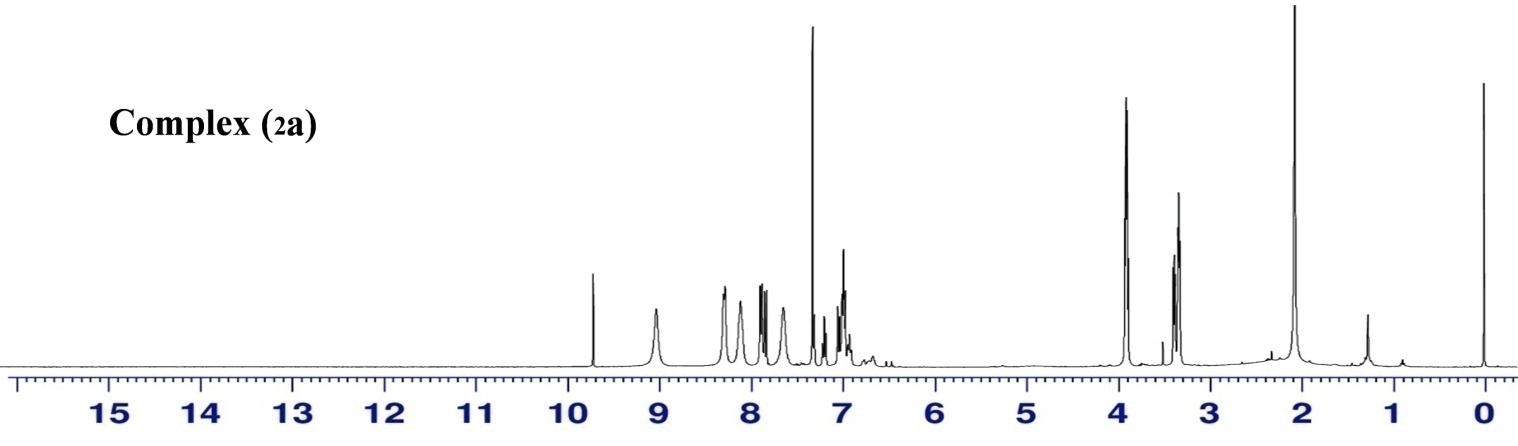  **Fig. S5** ^1^H NMR spectra of mixed ligand complex (**2a**) |
| --- |

***3.1.6. ^13^C NMR spectrum***

The ligand (**HL**) and complex (**2a**) were performed by a Bruker Advance III HD Nanobay 400 MHz spectrometer in the presence of CDCl_3_ at 25 ° C. The subsequent signals of free ligand and its complex (**2a**) were revealed in **Figs. S6** & **S7**. The obained peak at 156.84 ppm was assigned iminic (azomethine) carbons, which further shifted to the downfield region at165.34 ppm. It designates the coordination of iminic nitrogen to the metal centre in the complex (**2a**). The aromatic carbons of the ligand (**HL**) are noted in the range 113.47 – 135.88 ppm. The observed signal in the region 151.95–153.61 ppm indicates the carbon atom adjacent to the phenolic oxygen in the free ligand (**HL**). The signal is further transferred to the downfield region 158.52–160.11 in the complex (**2a**), which reveals that phenolic oxygen coordinated with the metal centre. The peak of morpholinic-CH_2_-O-CH_2_ was found at 66.64 ppm. The chemical shifts for =N-CH_2_-CH_2_-N and =N-CH_2_-CH_2_-N groups were also obtained at 65.19 and 63.35 respectively. The observed peaks at 47.87 and 47.29 ppm are ascribed to morpholinic-CH_2_-N-CH_2_ carbons. Moreover, the free 2,2'-bipyridine signals were found in the following range of 155–160 ppm (ArC^2,2'^), 145–150 ppm (ArC^6,6'^), 135–140 ppm (ArC^4,4'^), 125–130 ppm (ArC^5,5'^) and 115–120 ppm (ArC^3,3'^). Also, ArC^2,2'^, ArC^6,6'^ and ArC^4,4'^ signals are shifted to the downfield region at 162.51, 155.62, 141.35 ppm respectively, which indicates that the nitrogen atoms in bpy are coordinated with the Zn^2+^ center. Additionally, the CDCl_3_ solvent showed prominent triplet peaks in the 76.74–77.38 ppm range. The obtained data further suggest that the locations of carbons in the ligand structure and other signals are no substantial changes in the complex (**2a**).

| 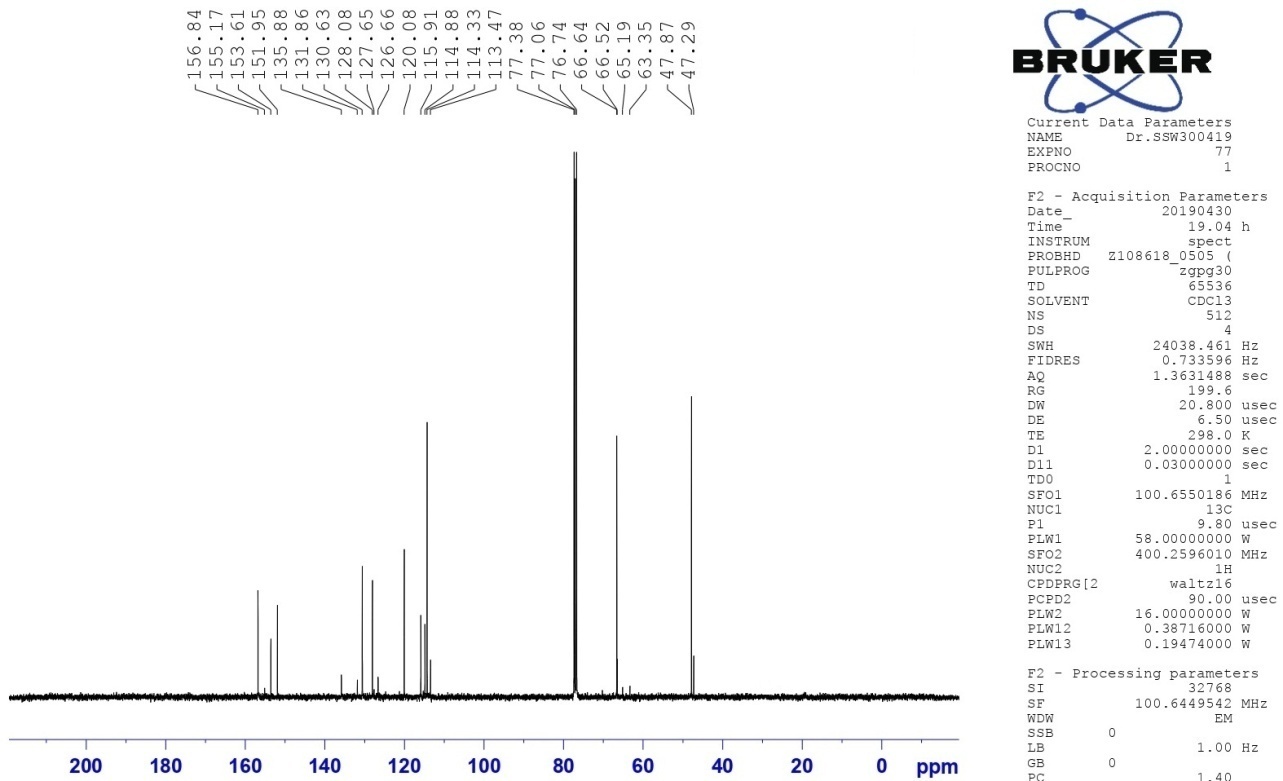  **Fig. S6** ^13^C NMR spectrum of ligand (**HL**). |
| --- |

| 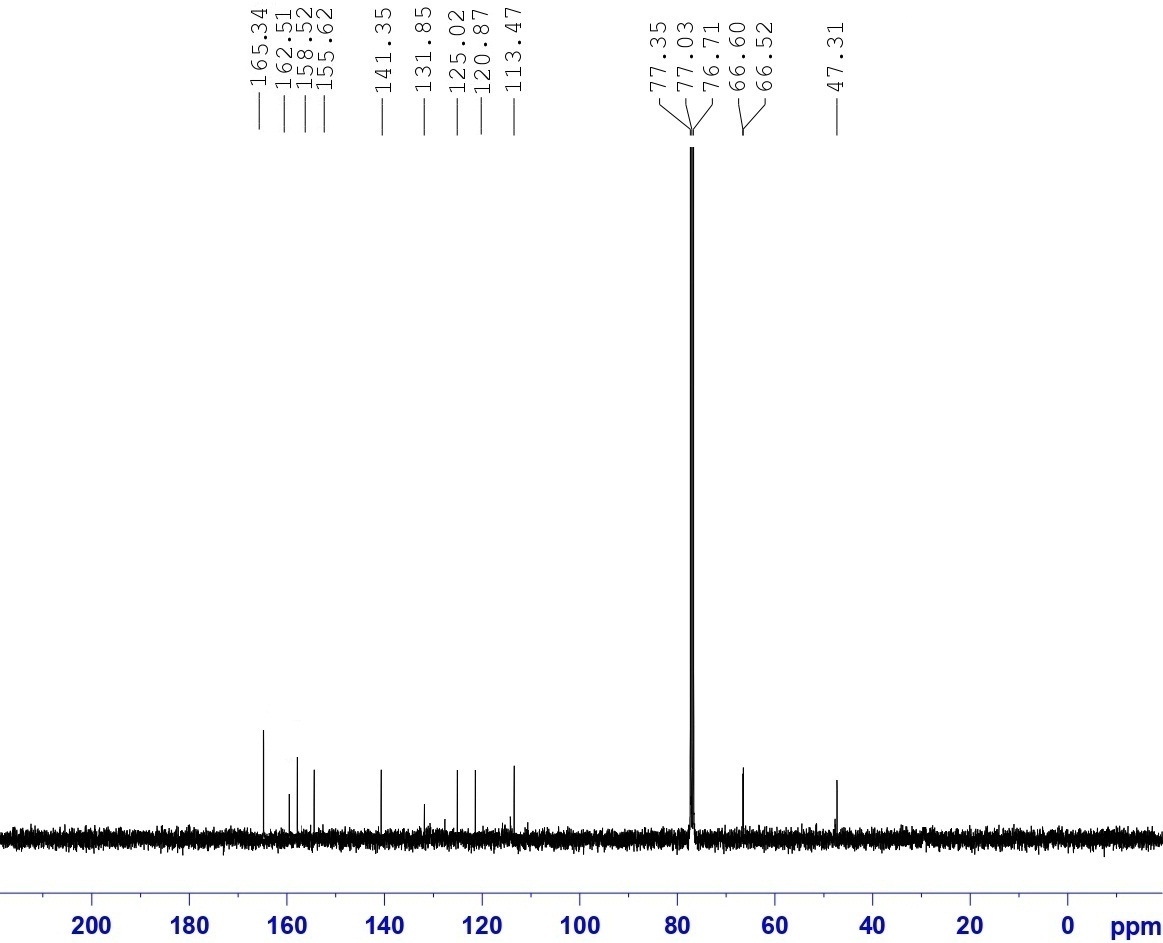  **Fig. S7** ^13^C NMR spectrum of mixed ligand complex (**2a**). |
| --- |

***3.1.7. FT-IR***

The FTIR spectra of the complexes (**1a**–2**a**) investigated the changes in frequency of free ligand due to the complexation and their attained findings were also enclosed in **Table S3.** The free ligand exhibits a strong sharp band of the iminic group (-HC=N-) at 1632 cm^–1^ the band is also shifted to lower frequencies in complexes (**1a**–2**a**) due to strong complexation with the central metal ion. The -OH group was found to have a strong peak at 3403 cm^–1^ in the free ligand, however this peak losed from the spectra of complexes (**1a**–2**a**) owing to the -OH group deprotonating during complexation. It is further evidenced that the observed phenolic C-O group at 1268 cm^–1^ in the free ligand is tranferred to higher frequencies (1304–1316 cm^–1^) in all complexes pointing out validated deprotonation of the phenolic–OH on chelation. In the FT-IR spectra of all complexes, the peaks consequent to the ring stretching frequencies γ(C=N) and γ(C=C) at 1503, 1422 cm^–1^ of free 2,2'-bipyridine were shifted to higher frequencies in the region of 1525–1536 cm^–1^ and 1432–1440 cm^–1^ respectively, which indicates the complexation owing to coordination of the heterocyclic nitrogen atoms to the metal ion. In this case, the observed medium bands in the region of 450 – 465 cm^–1^ and 518 – 524 cm^–1^ are consequent to M–N and M–O vibrations respectively and other absorption bands like morpholinic-C-N-C, morpholinic-C-O-C, aromatic C–H and aliphatic C–H have no appreciable changes in the complexes (**1a**–**2a**) (**Figs. S8, S8a & S8b**).

| **Table S3** FT-IR spectral data (cm^–1^) of the ligand (**HL**) and its complexes (**1a**–2**a**). | | | | | | | | | | | |
| --- | --- | --- | --- | --- | --- | --- | --- | --- | --- | --- | --- |
| **Com**  **pounds** | **HC=N** | **Ph-C-O** | **Morp-**  **C-N-C** | **Morp-**  **C-O-C** | **C-H** | | | **Ph-OH / H-bond** | **Bpy**  **C=N & C=C** | **M-N** | **M-O** |
|  |  |  |  |  | **Ar-**  **C-H** | **Ali-**  **C-H** | **Iminic**  **H-C=N** |  |  |  |  |
| (**HL**) | 1632 | 1268 | 1341 | 1112 (s)  1181(as) | 2974 | 2918 | 2858 | 3403 | --- | --- | --- |
| (**1a**) | 1616 | 1316 | 1364 | 1112 (s)  1173(as) | 2972 | 2926 | 2858 | --- | 1536  1440 | 450 | 524 |
| (**2a**) | 1610 | 1304 | 1349 | 1112 (s)  1181(as) | 2972 | 2926 | 2850 | --- | 1525  1432 | 465 | 518 |
| s → symmetry, as → asymmetry, Phen-C-O → Phenolic C-O, Morp-C-N-C → Morpholinic C-N-C, Morp-C-O-C → Morpholinic C-O-C, Ar-C-H → Aromatic C-H, Ali-C-H → Aliphatic C-H Ph-OH → Phenolic OH, bpy→ 2,2'-bipyridine. | | | | | | | | | | | |

| **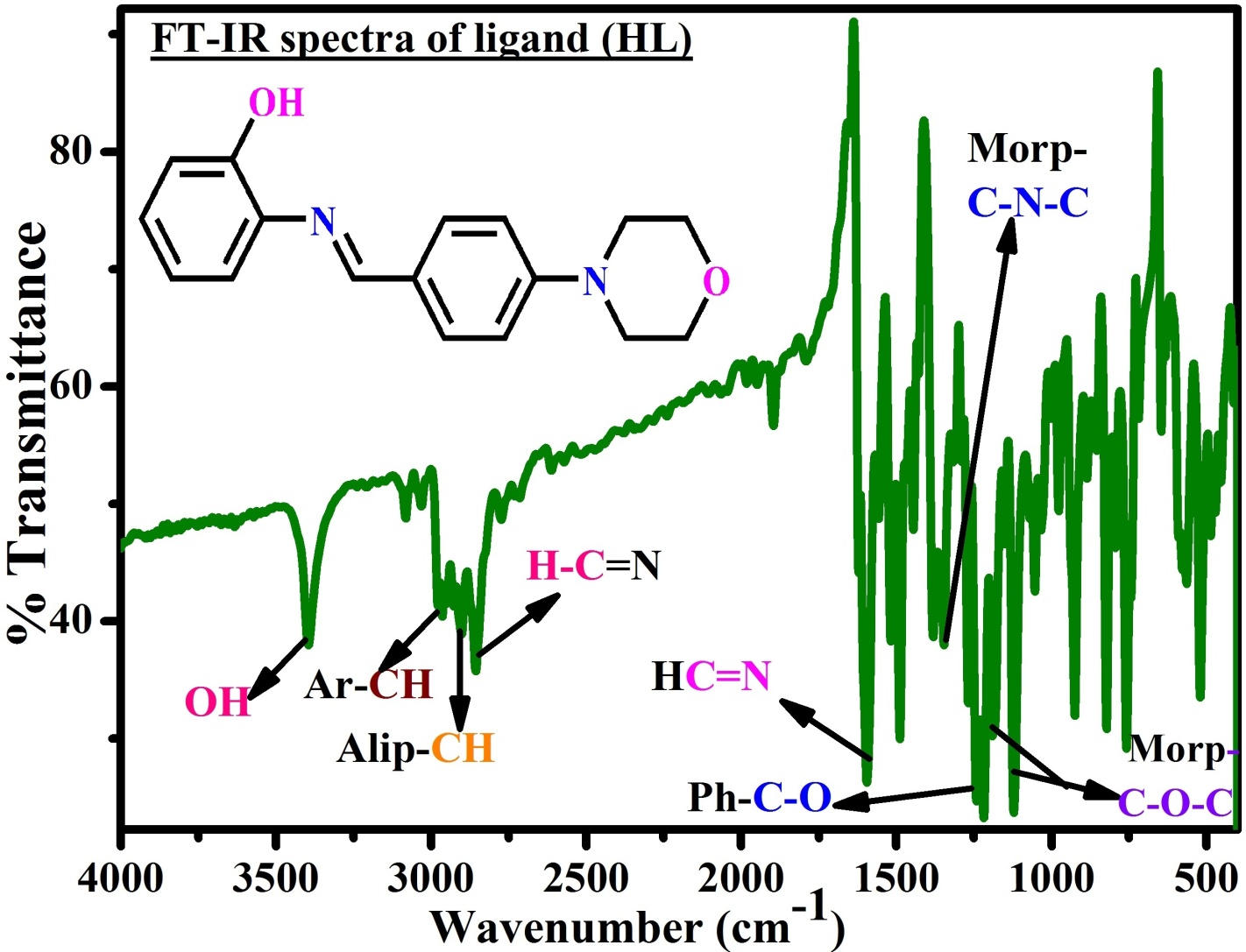**  **Fig. S8** FT-IR spectrum of free ligand (**HL**) |
| --- |

| **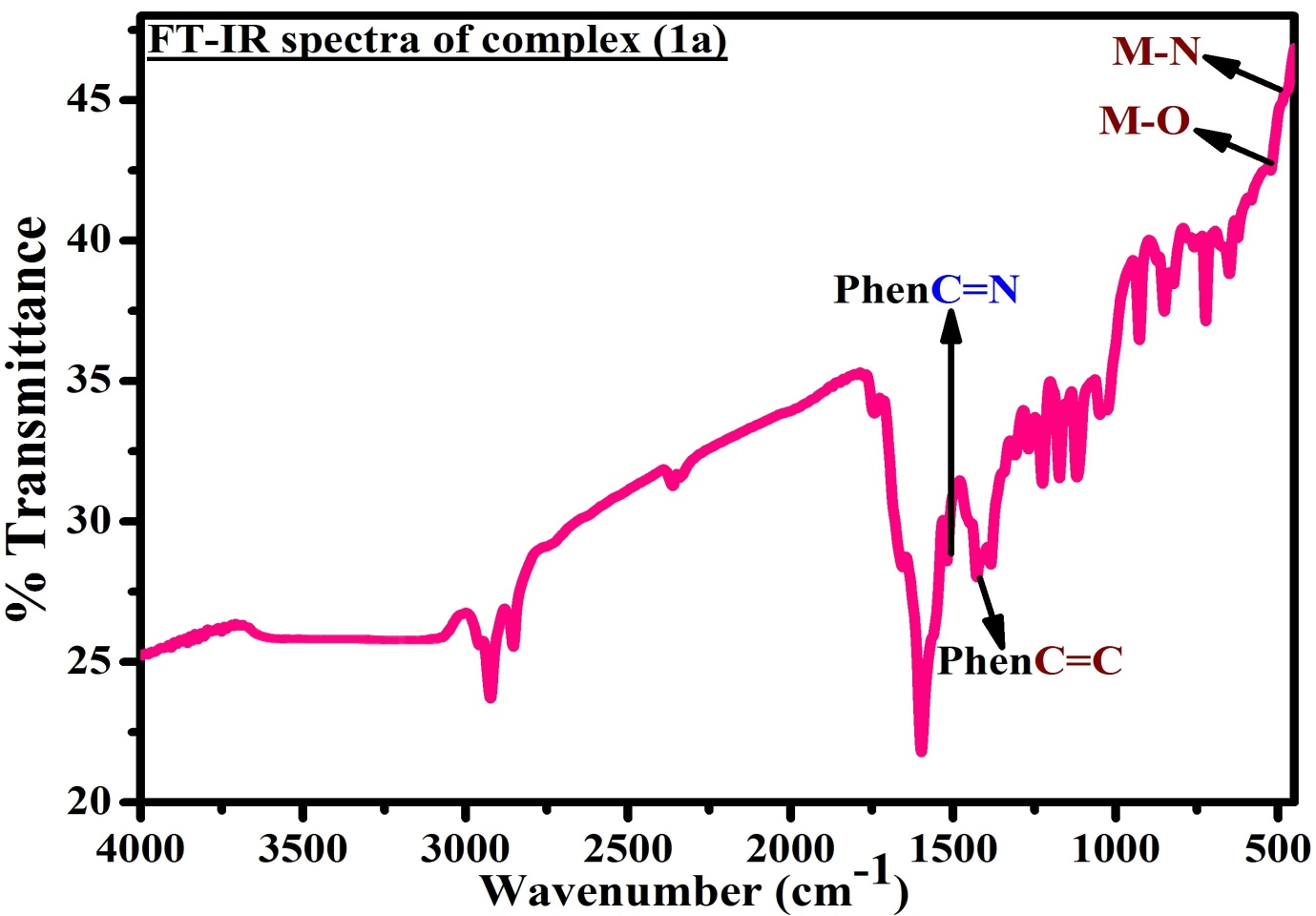**  **Fig. S8a** FT-IR spectrum of complexes (**1a**). |
| --- |

| **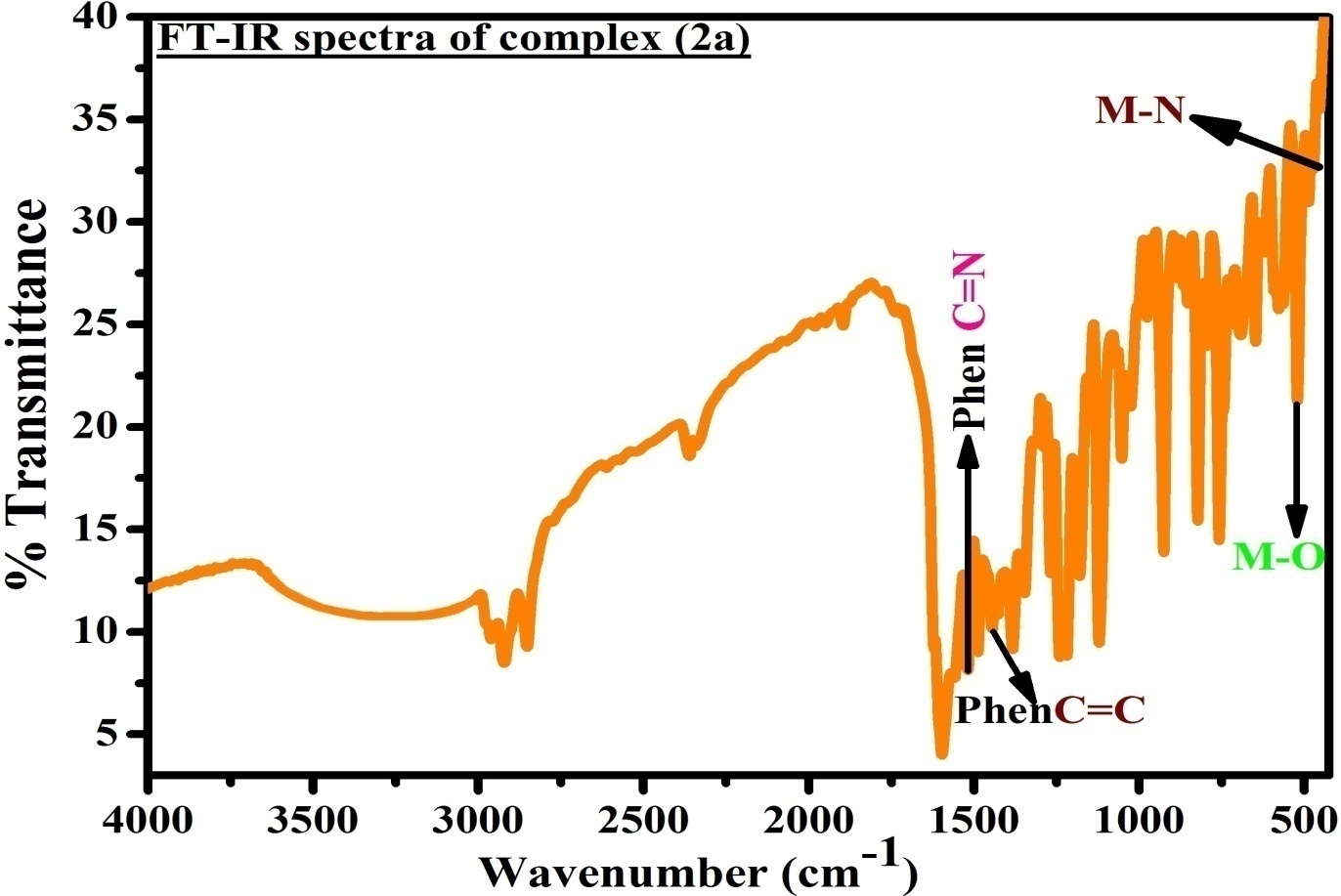**  **Fig. S8b** FT-IR spectrum of complexes (**2a**). |
| --- |

***3.1.8. UV–Visible spectra and Magnetic Susceptibility***

Free ligand and its complex (**1a**) were carried out in methanol by UV–Visible spectrophotometer. The observed results of absorption maxima and magnetic moment are summarized in **Table S4**. The ligand (**HL**) exhibited three absorption bands at 358 nm (27,933 cm^-1^), 247 nm (40,485 cm^-1^) and 215 nm (46,512 cm^-1^), which assigns n→π*, π→π* and π→π* transitions respectively owing to the azomethine chromophore and phenyl rings. The bands are transferred into a higher wavelength, which attributes the formation of complex due to the lone pair electron of an sp^2^-hybridized orbital of the imino nitrogen in the ligand donates to the metal centre. Moreover, mixed ligand complex (**1a**) revealed only one low-intensity broad band d-d transition at 652 nm (15,337 cm^-1^) corresponding to ^2^Eg→^2^T_2_g owing to dynamic Jahn–Teller distortion and the effective magnetic moment (μ_eff_) value is observed at 1.95 B.M, which is faintly greater than the spin-only value 1.73 BM. (**Table S4 & Fig. S9**). In addition, the obtained μ_eff_ value of the complex (**2a**) was zero, which indicates the diamagnetic nature. The obtained results suggest that the complex (**2a**) belong to an octahedral environment around the central metal(II) ion.

| **Table S4** Electronic spectral data and magnetic susceptibility values of the synthesized ligand (**HL**) and its complex (**1a**). | | | | |
| --- | --- | --- | --- | --- |
| **Compounds** | **Band Position**  **λ _max_ nm (γ-cm^-1^)** | **Assignment** | **μ_eff_**  **(B.M)** | **Geometry** |
| (**HL**) | 358 (27,933)  247 (40,485)  215 (46,512) | n→π^*^  π→π^*^  π→π^*^ | -- | -- |
| (**1a**) | 652 (15,337) | ^2^E_g_→^2^T_2g_ | 1.95 | Distorted octahedral |
| INCT→ Intra-ligand charge transfer, μ_eff_ → Effective magnetic moment, B.M→ Bohr magnetons. | | | | |

| 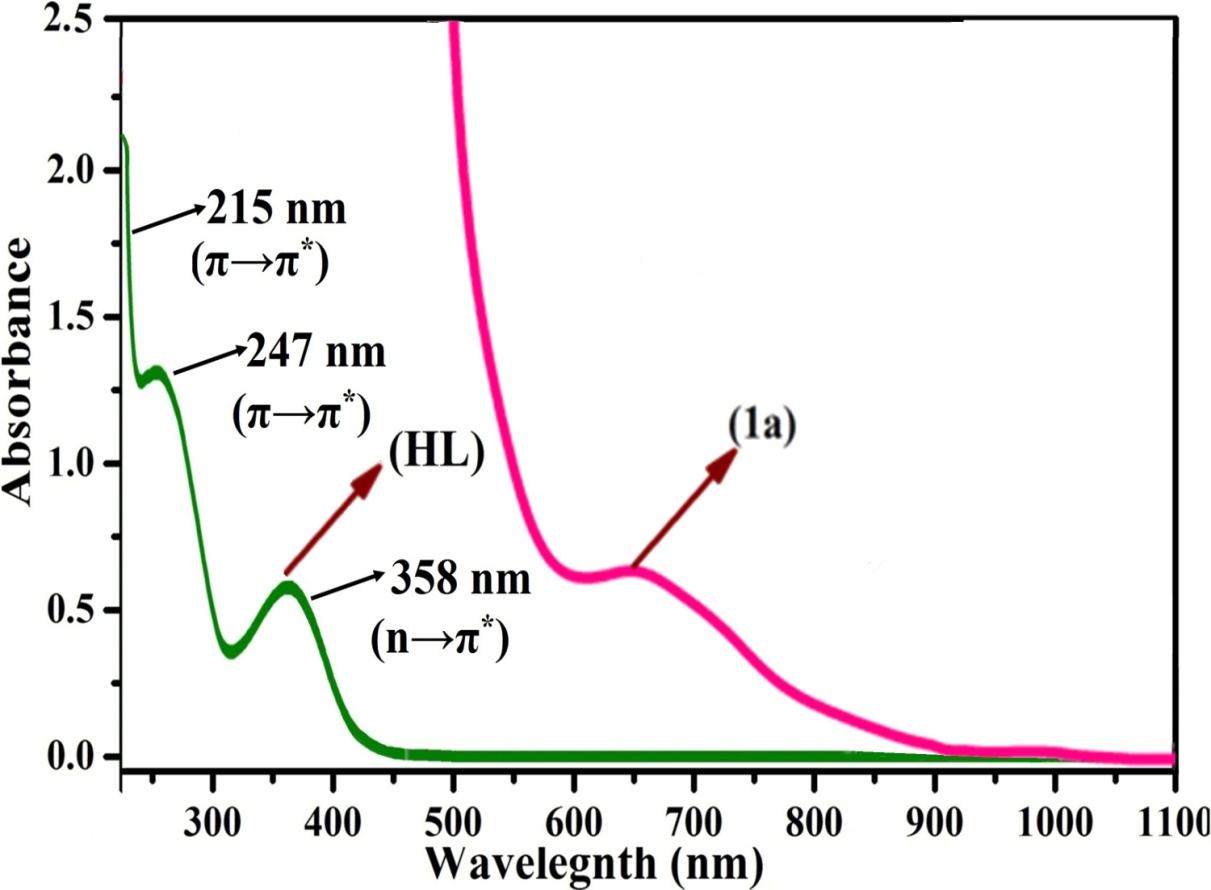  **Fig. S9** Electronic spectra of ligand (**HL**) and complex (**1a**). |
| --- |

***3.1.9. Electron Spin Resonance Spectra (ESR)***

In EPR spectra, copper complex (**1a**) exhibited an anisotropic pattern with well-resolved hyperfine lines in the X-band region at 77 K, which is revealed in **Fig S10** and the obtained results were enclosed in **Table** **S5**. The measured g-findings were noted in the subsequent sequence : g_||_ > g_⊥_ > g_e_ (2.00277) in the distorted square planar and octahedral copper complexes, which attribute that the unpaired electron lies in the $d_{x^{2}-y^{2}}$ orbital (^2^B_1g_) as the ground state and further confirm the covalent character of the M-L bond owing to g_||_ values were less than 2.3. The g_eff_ values of complex (**1a**) were observed at 1.394, which were further supportive of the covalent character of the M-L bond owing to their findings are less than 2.00277. The attained hyperfine constant parameters for complex (**1a**) were found in the following order: A_||_ > A_av_ > A_⊥_ and the co-factor (f_||_) values of the degree of geometrical distortion for these complexes were observed at 263.65 cm^-1^. The obtained results are proposed that complex (**1a**) have distorted octahedral geometry. The calculated G value for copper complexes was greater than 4, which attributed that the absence of interaction between Cu-Cu centres in the solid-state complex (**1a**) and the absence of half field signal at 1600 G consequent to the ΔMs = ± 2 transition rules out a Cu-Cu interaction. The molecular orbital coefficient parameters α^2^ (In-plane σ-bonding), β^2^ (in-plane π-bonding) and γ^2^ (out-plane π-bonding) for copper complex (**1a**) were measured from Kivelson and Neimann formulae (**Table** **S5)**. The observed α^2^, β^2^ and γ^2^ results were in the range of 0.428, 0.813 and 0.552 respectively, which are designated that the copper complex reveals the covalent character and further attributed that σ-bonding and π-bonding are entirely covalent character owing to their value are lower than 1.0. In addition, the obtained hyperfine interaction (K) value was found in the range of 0.274, which was less than 1.0 and it is more supportive to examine the covalent environment. The obtained orbital reduction factors K_║_ and K_⊥_ values for copper complex (**1a**) were observed at 0.348 and 0.236 respectively, which suggest the presence of out-plane π-bonding in metal-ligand π–bonding. The acquired findings of fermi contact hyperfine interaction term K_fermi_ for the copper complex were found at 0.238, which is further supported to assess the polarization generated by the uneven sharing of d-electron density on the inner core s-electron. All the observed findings recommend that copper complex (**1a**) has distorted octahedral geometry and the results are also good agreed with UV–Visible spectral data.

| **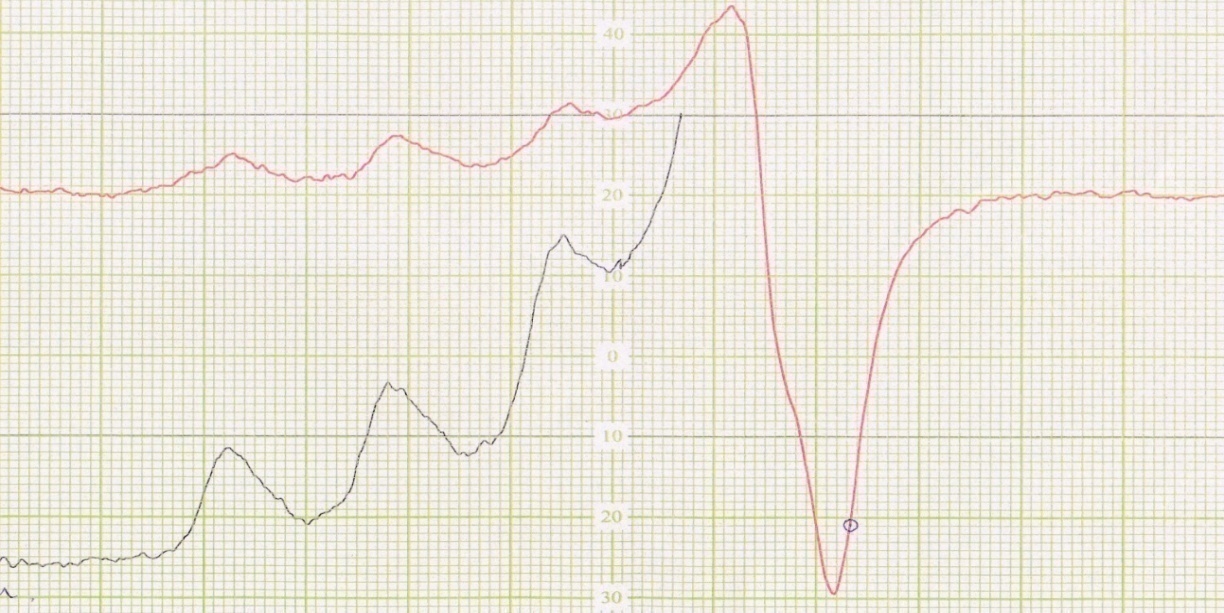**  **Fig. S10** EPR spectra of complex (**1a**) at liquid nitrogen temperature (77 K). |
| --- |

| **Table S5** The spin Hamiltonian parameters of complex (**1a**) at 77K. | | | | | | | | | | |
| --- | --- | --- | --- | --- | --- | --- | --- | --- | --- | --- |
| Complex (**1a**) | **g tensor** | | | |  | **Hyperfine constant × 10**^–^**^4^ (cm**^–^**^1^)** | | | | |
|  | $g_{\parallel}$ | $g_{\perp}$ | $g_{\mathrm{av}}$ | g_eff_ |  | $A_{\parallel}$ | $A_{\perp}$ | $A_{\mathrm{av}}$ | | |
|  | 2.1532 | 2.0283 | 2.0699 | 1.394 |  | 81.66 | 27.50 | 45.5555 | | |
|  | **Bonding Parameters** | | | | | | | | | |
|  | G | f_\|\|_ (cm^–1^) | α^2^ | β^2^ | γ^2^ | $K_{\vert\vert}^{2}$ | $K_{\perp}^{2}$ | K | K_fermi_ | µ_eff_ |
|  | 5.8922 | 263.65 | 0.4282 | 0.813 | 0.552 | 0.348 | 0.236 | 0.274 | 0.238 | 1.96 |
| g_e_ = 2.00277, Microwave frequency (γ’) = 9.114 × 10^9^cycle/sec, Scan range = 2000-3000 G, 1G = 10^–4^ cm^–1^, Electronic absorption$E_{d-d}$ = 15,337 cm^–1^ for complex (**1a**), one-electron spin orbit coupling constant of free Cu(II) ion λ_0_ = -828 cm^–1^, Magnetic susceptibility (µ_eff_) = 1.96 B.M, for complex (**1a**). Molecular orbital coefficient parameters α^2^, β^2^ and γ^2^, Co-factor (*f_\|\|_*) value of degree of geometrical distortion; Ionic environment (K, K_fermi_), Electron spin (S) = 1/2, G = Exchange interaction coupling constant. (**1**). $g_{\perp}=\frac{{(3g}_{\mathrm{av}}- g_{\vert\vert})}{2}$ ; (**2**).$K_{\perp}=\frac{({3A}_{\mathrm{av}}- A_{\vert\vert})}{2}$; (**3**).$g_{\mathrm{av}}=\frac{{(g}_{\vert\vert+} 2 g_{\perp})}{3}$ ; (**4**). $g_{eff}=\frac{(g_{\vert\vert}+ g_{\perp})}{3}$ ; (**5**).$A_{\mathrm{av}}=\frac{(A_{\vert\vert}+ 2A_{\perp})}{3}$ ; (**6**). $G=\frac{{( g}_{\vert\vert}- 2.00277)}{( g_{\perp}- 2.00277)}$ ; (**7**).$\alpha^{2}=\frac{A_{\vert\vert}}{P}+{(g}_{\vert\vert}- 2.00277)+\frac{3}{7}{(g}_{\perp}-2.00277)+0.04$ ; (**8**).${\beta^{2}=(g}_{\vert\vert}- 2.00277)(\frac{E_{d-d}}{-8\lambda_{0}a^{2}})$; (**9**).${\gamma^{2}=(g}_{\perp}-2.00277)(\frac{E_{d-d}}{-2\lambda_{0}a^{2}})$; (**10**).$K_{\vert\vert}^{2}={(g}_{\vert\vert}-2.00277)(\frac{E_{d-d}}{-8\lambda_{0}})$; (**11**). $K_{\perp}^{2}={(g}_{\perp}-2.00277)(\frac{E_{d-d}}{-2\lambda_{0}})$; (**12**).$f_{\vert\vert}$= $g_{\vert\vert}/A_{\vert\vert}$; (**13**). $K=\frac{{(K}_{\vert\vert}^{2} + 2K_{\perp}^{2})}{3}$ ; (**14**). $K_{\mathrm{fermi}}=\frac{{A_{\mathrm{av}}}}{P\beta^{2}}+ \frac{({g_{\mathrm{av}}}- 2.00277)}{\beta^{2}}$ ; (**15**). Free ion dipolar term$P=2 ϒ_{\mathrm{Cu}} \beta_{0}B_{N} (\gamma^{-3})$ = 0.036 cm^-1^; (**16**). $\mu_{\mathrm{eff}}=g_{av} {[S(S+1)]}^{\frac{1}{2}}$ ; ϒ_Cu_ = magnetic moment value for copper, β_0_ = Bohr Magneton, B_N_ = Nuclear Magneton, γ = the distance from the central nucleus to the electron. | | | | | | | | | | |

***3.1.10. Thermogravimetric Analysis (TGA)***

TGA thermograms of the complexes (**1a**–2**a**) have been performed in the temperature range of 40–730 °C (**Figs. S11 & S12**). All obtained date including the percentages of mass loss in experimentally and theoretically for all compounds are summarized in **Table S6**. The thermal stability of metal complexes is examined by controlling heating rates 10 °C per minute under nitrogen environment. Thermogram of the complexes (**1a**–**2a**) is stable up to 180 –200 °C and it attributed that there is no presenting of lattice and coordinated water molecules. Usually, lattice water and coordinated water molecules loses takes place in the range of 60–120 °C and 120–200 °C respectively. There are exposed three steps during the thermal decomposition of complexes. In the first step process at 40–180°C, the observed weight losses of decomposition of [M^II^ (L)_2_ (bpy)] complexes were found in the range of 10.85–11.08 % respectively, which corresponds to the loss of morpholine moieties (C_4_H_8_NO). In the second degradation stages, the weight losses were evaluated in the range of 180–420 °C, which is further confirmed that the elimination of the rest aromatic part of ligand (C_13_H_9_NO) present in the complexes and they are found in the range of 24.85–24.92 % for complexes (**1a**–**2a**). Similarly, the attained weight losses in the third degradation stage at 420–730 °C were pointed out in the range of 55.69–55.85 % elimination of ligand C_17_H_17_N_2_O (L) and co-ligands 2,2'-bipyridine [C_10_H_8_N_2_] in the mixed ligand complexes (**1a**–**2a**). The observed final percentage of rest products of metal oxide (MO) residue at above 730 °C was found in the range of 10.22–10.41 % for mixed ligand complexes (**1a**–**2a**). The thermal degradation steps of complexes (**1a**–**2a**) are shown in the **Fig. S11**. As per the observations, the proposed structures of all complexes have been demonstrated in the experimental section.

| **Table S6** Thermal analysis of complexes (**1a-2a**) by TGA method. | | | | |
| --- | --- | --- | --- | --- |
| **Complexes**  **(M.W)** | **Step** | **Temperature range (°C)** | **% Weight loss**  **[found (calcd)]** | **Assignment** |
| (**1a**) [Cu(L)_2_(bpy)] (C_44_H_42_N_6_O_4_)Cu  (781.68) | I | 40–180 | 11.08 (11.00) | C_4_H_8_NO |
|  | II | 180–420 | 24.85 (24.94) | C_13_H_9_NO |
|  | III | 420–730 | 55.85 (55.92) | C_17_H_17_N_2_O, C_10_H_8_N_2_ |
|  | Residue | >730 | 10.22 (10.17) | CuO |
| (**2a**) [Zn(L)_2_(bpy)] (C_44_H_42_N_6_O_4_)Zn  (783.55) | I | 40–180 | 10.85 (10.98) | C_4_H_8_NO |
|  | II | 180–420 | 24.92 (24.88) | C_13_H_9_NO |
|  | III | 420–730 | 55.69 (55.79) | C_17_H_17_N_2_O, C_10_H_8_N_2_ |
|  | Residue | >730 | 10.41 (10.38) | ZnO |
| M.W → Molecular Weight, TGA →Thermo Gravimetric Analysis. | | | | |

| 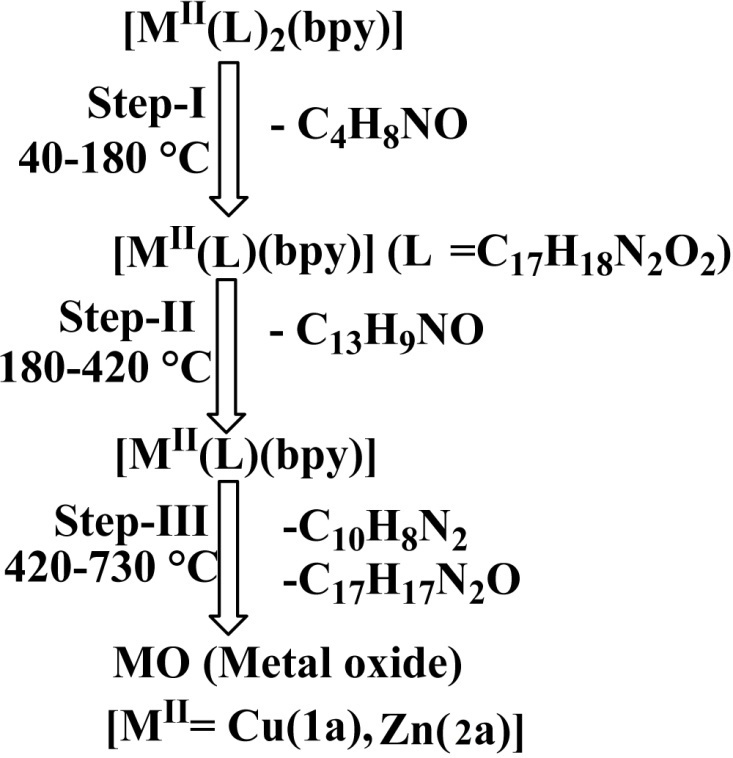  **Fig. S11** The various stages for thermal decomposition of complexes (**1a**–2**a**) at temperature range of 40–730 °C. |
| --- |

| 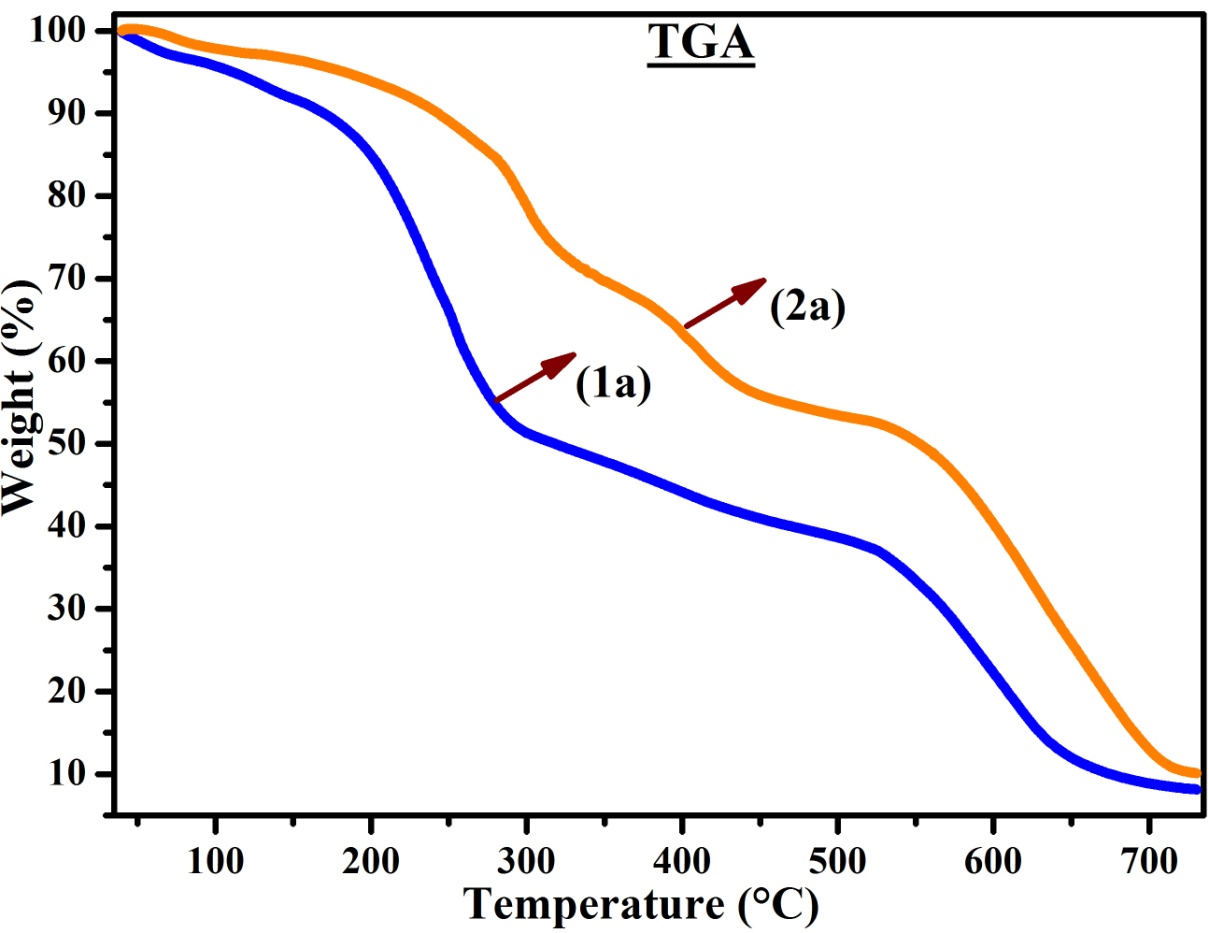  **Fig. S12** TG plots of complexes (**1a**–2**a**) recorded under nitrogen atmosphere between the temperature range 40 and 750 °C at a heating rate of 20° C/min. |
| --- |

***3.1.11. Single-Crystal X-RD Analysis***

The free ligand was obtained as a light yellow coloured single crystalline form in the presence of ethyl acetate medium by slow evaporation of the chloroform and ethanol mixture solution. Single-crystal XRD analysis of Schiff base ligand (**HL**) was performed to attain comprehensive data on molecular conformations in the solid-state. This analysis also proves the molecular structure and atom connectivity as shown in **Figs.** **S13** & **S14**. The information on the XRD data collection and structure refinements are certained in **Table S7** and H-bonds are given in **Table S8**. The crystallographic data were gathered with the assistance of MoKα radiation in the wavelength of 0.71073 Å by Bruker AXS KAPPA APEX-2 diffractometer operated with a graphite monochromator. The molecular conformation of ligand was resolved by direct approaches and refined by full-matrix least-squares calculations using the SHELXL-2014 program. The ligand (**HL**) crystalline form was isomorphous in the monoclinic system and its space group P21/c with molecular formula C_17_H_18_N_2_O_2_ in the unit cell, which is also deposited in our previous report. However, the crystal packing is stabilized *via* intra-molecular hydrogen bonding interactions (O-H--N) and π–π interactions. The H-bonding dimensions are summarized in **Table S8** and the packing arrangement of molecules is demonstrated in **Fig. S14**. Moreover, the morpholine fragments are stabilized in the crystal packing by forming intermolecular hydrogen bonds C(2)-H(2)...O(2) between the morpholine ring oxygen O(2) and the hydrogen atom of phenyl ring [C(2)-H(2)] and the packing may also be completed by hydrophobic van der Waals force attractions between the morpholine bearing ligands. In addition, the intramolecular hydrogen bonds O(1)-H...N between the hydroxyl group and iminic group, may lead to the further stabilization of the molecular structure of the ligand (**HL**) (**Table S8**). In addition, some other crystal data including structure refinement, atomic coordinates, bond lengths [Å] and angles [°], equivalent isotropic displacement and anisotropic displacement parameters for the ligand (**HL**) are also summarized in the section (**Tables S8 – S13**).

| 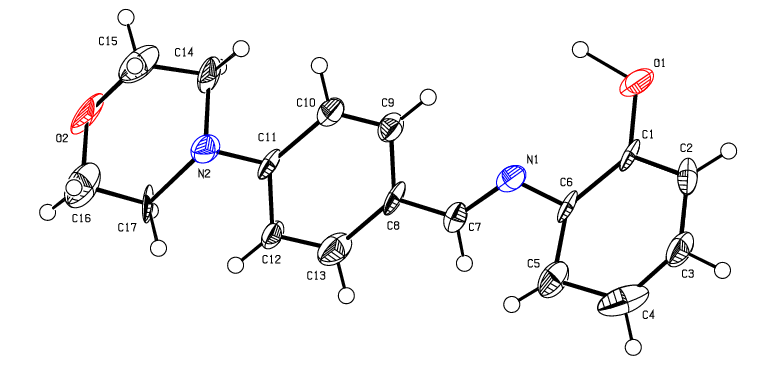  **Fig. S13.** The crystal structure of the ligand (**HL**) in ORTEP view. |
| --- |

| **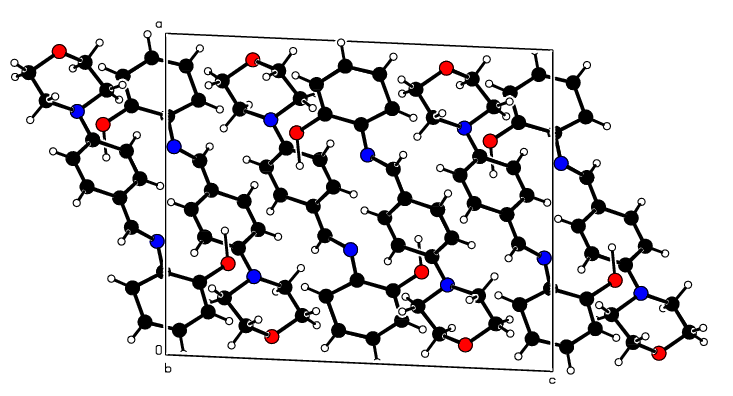**  **Fig.S14.** Unit cell packing diagram of the crystal ligand (**HL**). |
| --- |

| **Table S7** Crystal data and structure refinement for the ligand (**HL**). | |
| --- | --- |
| Empirical formula | C_17_ H_18_ N_2_ O_2_ |
| Formula weight | 282.33 |
| Temperature | 101 (2) K |
| Wavelength | 0.71073 Å |
| Crystal system | Monoclinic |
| Space group | P2_1_/c |
| Unit cell dimensions | a = 14.86 78 (12) Å, α = 90°  b = 7.5710 (6) Å, β = 93.197 (2)°  c = 12.3380 (8) Å, γ = 90° |
| Volume | 1386.65 (18) Å^3^ |
| Z, calculated Density | 4, 1.352 Mg/m^3^ |
| Absorption coefficient | 0.090 mm^-1^ |
| F(000) | 600 |
| Crystal size | 0.312 × 0.311 × 0.078 mm |
| Theta range for data collection | θ = 2.744 to 25.332°. |
| Limiting indices (Index ranges) | h = –17 → 17  k = –09 → 09  l = –14 → 14 |
| Reflections collected | 37050 |
| Independent reflections | 2505 [R(int) = 0.5296] |
| Completeness to ∆ = 25.242° | 98.8 % |
| Refinement method | Full-matrix least-squares on F2 |
| Data / restraints / parameters | 2505 / 0 / 218 |
| Goodness-of-fit on F2 | 1.222 |
| Final R indices [I > 2 σ (I)] | R1 = 0.1442, wR2 = 0.1293 |
| R indices (all data) | R1 = 0.1002, wR2 = 0.0694 |
| Largest diff. peak and hole | 0.987 and –0.546 e.Å^-3^ |

| **Table S8** Hydrogen bonds for the ligand (**HL**) (Å & °). | | | | |
| --- | --- | --- | --- | --- |
| **D-H...A** | **d(D-H)** | **d(H...A)** | **d(D...A)** | **<(DHA)** |
| C(2)-H(2)...O(2)#1 | 0.93 | 2.53 | 3.255(12) | 135.2 |
| Symmetry transformations used to generate equivalent atoms: #1 x-1,-y+3/2,z+1/2 | | | | |

| **Table S9** Atomic coordinates ( ×104) and equivalent isotropic displacement parameters (Å2 × 103) for Schiff base ligand (**HL**). U(eq) is defined as one third of the trace of the orthogonalized Uij tensor for the ligand (**HL**). | | | | |
| --- | --- | --- | --- | --- |
| **Atoms** | **x** | **y** | **z** | **U(eq)** |
| C(1) | 2302(6) | 7693(10) | 5879(6) | 26(2) |
| C(2) | 1389(7) | 7688(11) | 6100(6) | 35(2) |
| C(3) | 781(7) | 7005(11) | 5358(7) | 41(3) |
| C(4) | 989(7) | 6332(12) | 4470(7) | 58(3) |
| C(5) | 2036(6) | 6333(11) | 4147(5) | 43(3) |
| C(6) | 2575(6) | 6999(10) | 4947(5) | 26(2) |
| C(7) | 3919(7) | 6180(11) | 4117(6) | 30(2) |
| C(8) | 4821(6) | 6283(11) | 3817(6) | 31(2) |
| C(9) | 5443(7) | 7397(10) | 4336(6) | 31(2) |
| C(10) | 6282(7) | 7544(11) | 3989(6) | 34(2) |
| C(11) | 6589(6) | 6600(10) | 3118(6) | 27(2) |
| C(12) | 5979(7) | 5463(11) | 2607(6) | 32(2) |
| C(13) | 5122(8) | 5356(12) | 2971(7) | 44(3) |
| C(14) | 8153(7) | 7373(13) | 3479(6) | 41(2) |
| C(15) | 8989(8) | 7814(13) | 2922(8) | 56(3) |
| C(16) | 8596(8) | 6145(14) | 1467(9) | 61(3) |
| C(17) | 7742(7) | 5617(12) | 1916(6) | 35(2) |
| N(1) | 3516(6) | 7145(9) | 4780(5) | 34(2) |
| N(2) | 7452(6) | 6836(9) | 2719(5) | 37(2) |
| O(1) | 2918(5) | 8375(9) | 6614(4) | 51(2) |
| O(2) | 9292(5) | 6503(10) | 2254(5) | 64(2) |

| **Table S10** Bond lengths [Å] and angles [°] for the ligand (**HL**). | |
| --- | --- |
| **Positions of atoms** | **Bond lengths [Å] and**  **bond angles [°]** |
| C(1)-C(2) | 1.197(11) |
| C(1)-O(1) | 1.420(10) |
| C(1)-C(6) | 1.550(12) |
| C(2)-C(3) | 1.423(13) |
| C(2)-H(2) | 0.93 |
| C(3)-C(4) | 1.466(14) |
| C(3)-H(3) | 0.93 |
| C(4)-C(5) | 1.410(5) |
| C(4)-H(4) | 0.93 |
| C(5)-C(6) | 1.446(5) |
| C(5)-H(5) | 0.93 |
| C(6)-N(1) | 1.215(10) |
| C(7)-C(8) | 1.229(11) |
| C(7)-N(1) | 1.360(11) |
| C(7)-H(7) | 0.93 |
| C(8)-C(9) | 1.382(12) |
| C(8)-C(13) | 1.521(13) |
| C(9)-C(10) | 1.193(11) |
| C(9)-H(9) | 0.93 |
| C(10)-C(11) | 1.562(12) |
| C(10)-H(10) | 0.93 |
| C(11)-N(2) | 1.266(10) |
| C(11)-C(12) | 1.377(11) |
| C(12)-C(13) | 1.223(12) |
| C(12)-H(12) | 0.93 |
| C(13)-H(13) | 0.93 |
| C(14)-C(15) | 1.405(13) |
| C(14)-N(2) | 1.467(12) |
| C(14)-H(14A) | 0.97 |
| C(14)-H(14B) | 0.97 |
| C(15)-O(2) | 1.491(12) |
| C(15)-H(15A) | 0.97 |
| C(15)-H(15B) | 0.97 |
| C(16)-C(17) | 1.348(13) |
| C(16)-O(2) | 1.463(13) |
| C(16)-H(16A) | 0.97 |
| C(16)-H(16B) | 0.97 |
| C(17)-N(2) | 1.587(11) |
| C(17)-H(17A) | 0.97 |
| C(17)-H(17B) | 0.97 |
| O(1)-H(1O) | 1.28(14) |
| C(2)-C(1)-O(1) | 105.8(8) |
| C(2)-C(1)-C(6) | 119.8(8) |
| O(1)-C(1)-C(6) | 134.4(7) |
| C(1)-C(2)-C(3) | 105.2(8) |
| C(1)-C(2)-H(2) | 127.4 |
| C(3)-C(2)-H(2) | 127.4 |
| C(2)-C(3)-C(4) | 137.5(8) |
| C(2)-C(3)-H(3) | 111.3 |
| C(4)-C(3)-H(3) | 111.3 |
| C(5)-C(4)-C(3) | 120.9(8) |
| C(5)-C(4)-H(4) | 119.5 |
| C(3)-C(4)-H(4) | 119.5 |
| C(4)-C(5)-C(6) | 96.7(7) |
| C(4)-C(5)-H(5) | 131.6 |
| C(6)-C(5)-H(5) | 131.6 |
| N(1)-C(6)-C(5) | 106.2(7) |
| N(1)-C(6)-C(1) | 113.9(7) |
| C(5)-C(6)-C(1) | 139.6(7) |
| C(8)-C(7)-N(1) | 127.1(9) |
| C(8)-C(7)-H(7) | 116.5 |
| N(1)-C(7)-H(7) | 116.5 |
| C(7)-C(8)-C(9) | 109.4(8) |
| C(7)-C(8)-C(13) | 122.4(9) |
| C(9)-C(8)-C(13) | 128.1(8) |
| C(10)-C(9)-C(8) | 107.1(9) |
| C(10)-C(9)-H(9) | 126.5 |
| C(8)-C(9)-H(9) | 126.5 |
| C(9)-C(10)-C(11) | 124.8(9) |
| C(9)-C(10)-H(10) | 117.6 |
| C(11)-C(10)-H(10) | 117.6 |
| N(2)-C(11)-C(12) | 106.8(8) |
| N(2)-C(11)-C(10) | 124.8(8) |
| C(12)-C(11)-C(10) | 128.3(8) |
| C(13)-C(12)-C(11) | 105.4(9) |
| C(13)-C(12)-H(12) | 127.3 |
| C(11)-C(12)-H(12) | 127.3 |
| C(12)-C(13)-C(8) | 126.3(9) |
| C(12)-C(13)-H(13) | 116.9 |
| C(8)-C(13)-H(13) | 116.9 |
| C(15)-C(14)-N(2) | 92.2(8) |
| C(15)-C(14)-H(14A) | 113.3 |
| N(2)-C(14)-H(14A) | 113.3 |
| C(15)-C(14)-H(14B) | 113.3 |
| N(2)-C(14)-H(14B) | 113.3 |
| H(14A)-C(14)-H(14B) | 110.6 |
| C(14)-C(15)-O(2) | 116.6(8) |
| C(14)-C(15)-H(15A) | 108.1 |
| O(2)-C(15)-H(15A) | 108.1 |
| C(14)-C(15)-H(15B) | 108.1 |
| O(2)-C(15)-H(15B) | 108.1 |
| H(15A)-C(15)-H(15B) | 107.3 |
| C(17)-C(16)-O(2) | 96.1(9) |
| C(17)-C(16)-H(16A) | 112.5 |
| O(2)-C(16)-H(16A) | 112.5 |
| C(17)-C(16)-H(16B) | 112.5 |
| O(2)-C(16)-H(16B) | 112.5 |
| H(16A)-C(16)-H(16B) | 110 |
| C(16)-C(17)-N(2) | 114.0(8) |
| C(16)-C(17)-H(17A) | 108.7 |
| N(2)-C(17)-H(17A) | 108.7 |
| C(16)-C(17)-H(17B) | 108.7 |
| N(2)-C(17)-H(17B) | 108.7 |
| H(17A)-C(17)-H(17B) | 107.6 |
| C(6)-N(1)-C(7) | 119.3(7) |
| C(11)-N(2)-C(14) | 99.3(7) |
| C(11)-N(2)-C(17) | 119.8(7) |
| C(14)-N(2)-C(17) | 127.9(7) |
| C(1)-O(1)-H(1O) | 116(5) |
| C(16)-O(2)-C(15) | 121.0(7) |
| Symmetry transformations used to generate equivalent atoms | |

| **Table S11** Anisotropic displacement parameters (Å^2^ × 10^3^) for the ligand (**HL**). The anisotropic displacement factor exponent takes the form: -2π^2^[ h^2^a*^2^U^11^ + ... + 2 h k a* b* U^12^ ]. | | | | | | |
| --- | --- | --- | --- | --- | --- | --- |
| **Atoms** | **U^11^** | **U^22^** | **U^33^** | **U^23^** | **U^13^** | **U^12^** |
| C(1) | 8(3) | 24(3) | 48(4) | 6(3) | 10(3) | -1(2) |
| C(2) | 32(6) | 30(5) | 44(6) | 1(4) | 23(5) | 1(4) |
| C(3) | 19(5) | 38(6) | 66(7) | -5(5) | 10(5) | 1(4) |
| C(4) | 44(7) | 29(6) | 99(9) | -1(6) | -32(6) | -2(5) |
| C(5) | 19(5) | 48(6) | 62(7) | 3(5) | 1(4) | 9(4) |
| C(6) | 8(3) | 24(3) | 48(4) | 6(3) | 10(3) | -1(2) |
| C(7) | 18(5) | 36(5) | 36(5) | 2(4) | 5(4) | 1(4) |
| C(8) | 7(4) | 34(5) | 53(6) | 5(5) | 8(4) | 3(4) |
| C(9) | 24(5) | 25(5) | 44(6) | -4(4) | 8(4) | -4(4) |
| C(10) | 24(5) | 38(5) | 42(6) | -4(5) | 5(4) | -8(4) |
| C(11) | 8(4) | 25(5) | 47(6) | 5(4) | 5(4) | 0(4) |
| C(12) | 15(5) | 34(5) | 47(6) | -11(5) | 9(4) | -3(4) |
| C(13) | 26(6) | 37(5) | 70(7) | -9(5) | 0(5) | 1(4) |
| C(14) | 14(5) | 53(6) | 56(6) | -4(5) | 11(4) | 8(4) |
| C(15) | 24(6) | 47(7) | 97(9) | -1(6) | -6(6) | 1(5) |
| C(16) | 31(6) | 47(7) | 108(10) | -5(6) | 14(6) | 10(5) |
| C(17) | 21(5) | 38(5) | 49(6) | -4(5) | 28(4) | 5(4) |
| N(1) | 24(4) | 38(5) | 41(5) | -3(4) | -1(3) | -7(3) |
| N(2) | 21(4) | 40(5) | 50(5) | -14(4) | 1(4) | 4(3) |
| O(1) | 23(4) | 58(4) | 71(5) | -22(4) | 0(3) | -13(3) |
| O(2) | 7(3) | 74(5) | 111(6) | -14(5) | 8(4) | 1(3) |

| **Table S12** Hydrogen coordinates ( × 10^4^) and isotropic displacement parameters (Å^2^ × 10^3^) for the ligand (**HL**). | | | | |
| --- | --- | --- | --- | --- |
| **Atoms** | **x** | **y** | **z** | **U(eq)** |
| H(2) | 1133 | 8053 | 6642 | 42 |
| H(3) | 52 | 6977 | 5467 | 49 |
| H(4) | 422 | 5898 | 4114 | 70 |
| H(5) | 2295 | 6012 | 3599 | 52 |
| H(7) | 3463 | 5353 | 3862 | 36 |
| H(9) | 5245 | 7933 | 4858 | 37 |
| H(10) | 6792 | 8274 | 4257 | 41 |
| H(12) | 6178 | 4895 | 2095 | 38 |
| H(13) | 4604 | 4640 | 2704 | 53 |
| H(14A) | 7874 | 8346 | 3800 | 49 |
| H(14B) | 8339 | 6442 | 3886 | 49 |
| H(15A) | 9622 | 8078 | 3297 | 68 |
| H(15B) | 8784 | 8856 | 2604 | 68 |
| H(16A) | 8442 | 7165 | 1112 | 74 |
| H(16B) | 8880 | 5247 | 1095 | 74 |
| H(17A) | 7886 | 4473 | 2148 | 42 |
| H(17B) | 7122 | 5540 | 1503 | 42 |
| H(1O) | 3940(110) | 8420(160) | 6530(80) | 110(40) |

| **Table S13** Torsion angles [°] for for the ligand (**HL**). | |
| --- | --- |
| **Positions of atoms** | **Torsion angles [°]** |
| O(1)-C(1)-C(2)-C(3) | 179.9(6) |
| C(6)-C(1)-C(2)-C(3) | -1.0(11) |
| C(1)-C(2)-C(3)-C(4) | 1.4(15) |
| C(2)-C(3)-C(4)-C(5) | -3.2(16) |
| C(3)-C(4)-C(5)-C(6) | 3.5(11) |
| C(4)-C(5)-C(6)-N(1) | -177.9(7) |
| C(4)-C(5)-C(6)-C(1) | -4.6(13) |
| C(2)-C(1)-C(6)-N(1) | 176.6(9) |
| O(1)-C(1)-C(6)-N(1) | -4.7(13) |
| C(2)-C(1)-C(6)-C(5) | 3.8(15) |
| O(1)-C(1)-C(6)-C(5) | -177.6(9) |
| N(1)-C(7)-C(8)-C(9) | 7.8(13) |
| N(1)-C(7)-C(8)-C(13) | -169.2(8) |
| C(7)-C(8)-C(9)-C(10) | -175.5(9) |
| C(13)-C(8)-C(9)-C(10) | 1.3(13) |
| C(8)-C(9)-C(10)-C(11) | -0.9(13) |
| C(9)-C(10)-C(11)-N(2) | 175.4(10) |
| C(9)-C(10)-C(11)-C(12) | -0.3(15) |
| N(2)-C(11)-C(12)-C(13) | -175.1(8) |
| C(10)-C(11)-C(12)-C(13) | 1.2(13) |
| C(11)-C(12)-C(13)-C(8) | -0.8(13) |
| C(7)-C(8)-C(13)-C(12) | 176.0(10) |
| C(9)-C(8)-C(13)-C(12) | -0.4(15) |
| N(2)-C(14)-C(15)-O(2) | -49.9(10) |
| O(2)-C(16)-C(17)-N(2) | 46.5(10) |
| C(5)-C(6)-N(1)-C(7) | -23.2(11) |
| C(1)-C(6)-N(1)-C(7) | 161.6(7) |
| C(8)-C(7)-N(1)-C(6) | 173.3(9) |
| C(12)-C(11)-N(2)-C(14) | -156.1(7) |
| C(10)-C(11)-N(2)-C(14) | 27.4(10) |
| C(12)-C(11)-N(2)-C(17) | -11.9(10) |
| C(10)-C(11)-N(2)-C(17) | 171.6(7) |
| C(15)-C(14)-N(2)-C(11) | -171.9(7) |
| C(15)-C(14)-N(2)-C(17) | 48.1(11) |
| C(16)-C(17)-N(2)-C(11) | 171.0(9) |
| C(16)-C(17)-N(2)-C(14) | -56.0(13) |
| C(17)-C(16)-O(2)-C(15) | -61.2(11) |
| C(14)-C(15)-O(2)-C(16) | 72.9(12) |
| Symmetry transformations used to generate equivalent atoms | |

**Supplementary Figures**

| 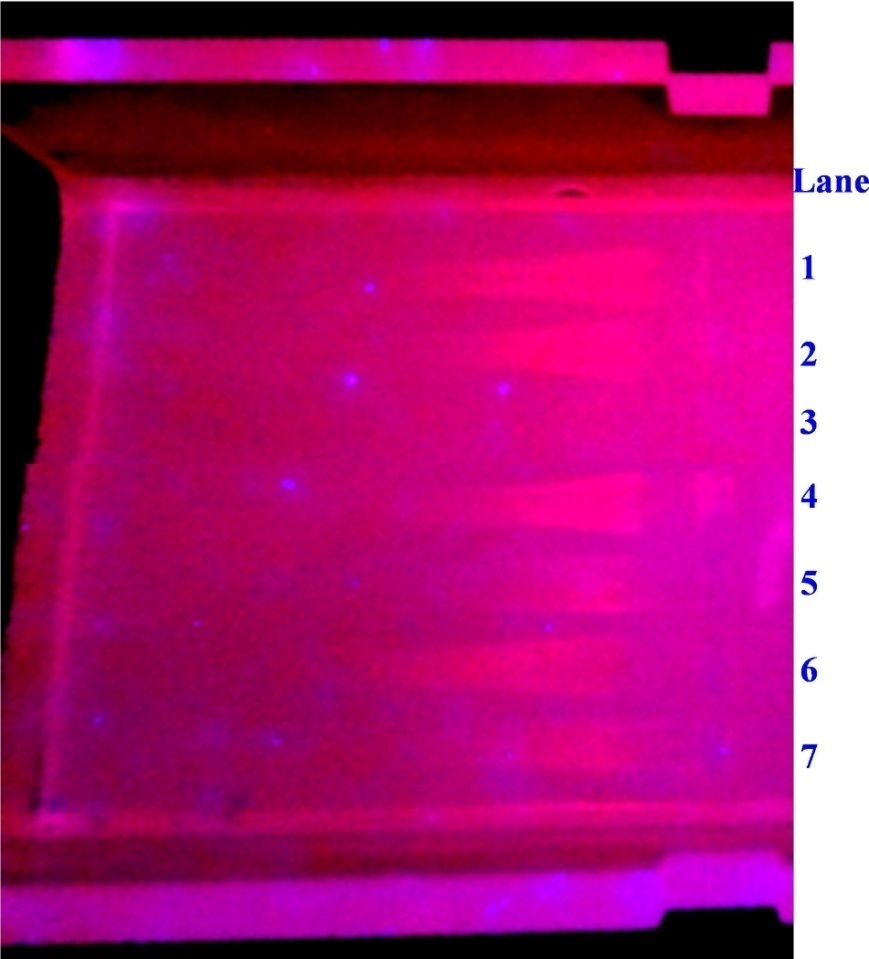  **Fig. S15.** Raw data for electrophoretic gels and blots showing the chemical nuclease activity of CT-DNA by the synthesized ligand (**HL**) and its mixed ligand complexes (**1a**–2**a**) in the presence of hydrogen peroxide. Lane: 1 DNA alone + H_2_O_2_ ; Lane: 2 ligand (**HL)** + DNA + H_2_O_2_ ; Lane: 3 complex (**1a**) + DNA + H_2_O_2_; Lane: 7 complex (**2a**) + DNA + H_2_O_2_ and excluding Lanes: (4–6), which belongs to another scheme. |
| --- |

|   **Fig. 16.** Fenton and Haber-Weiss mechanisms for DNA cleavage in the H_2_O_2_ environment. |
| --- |

| **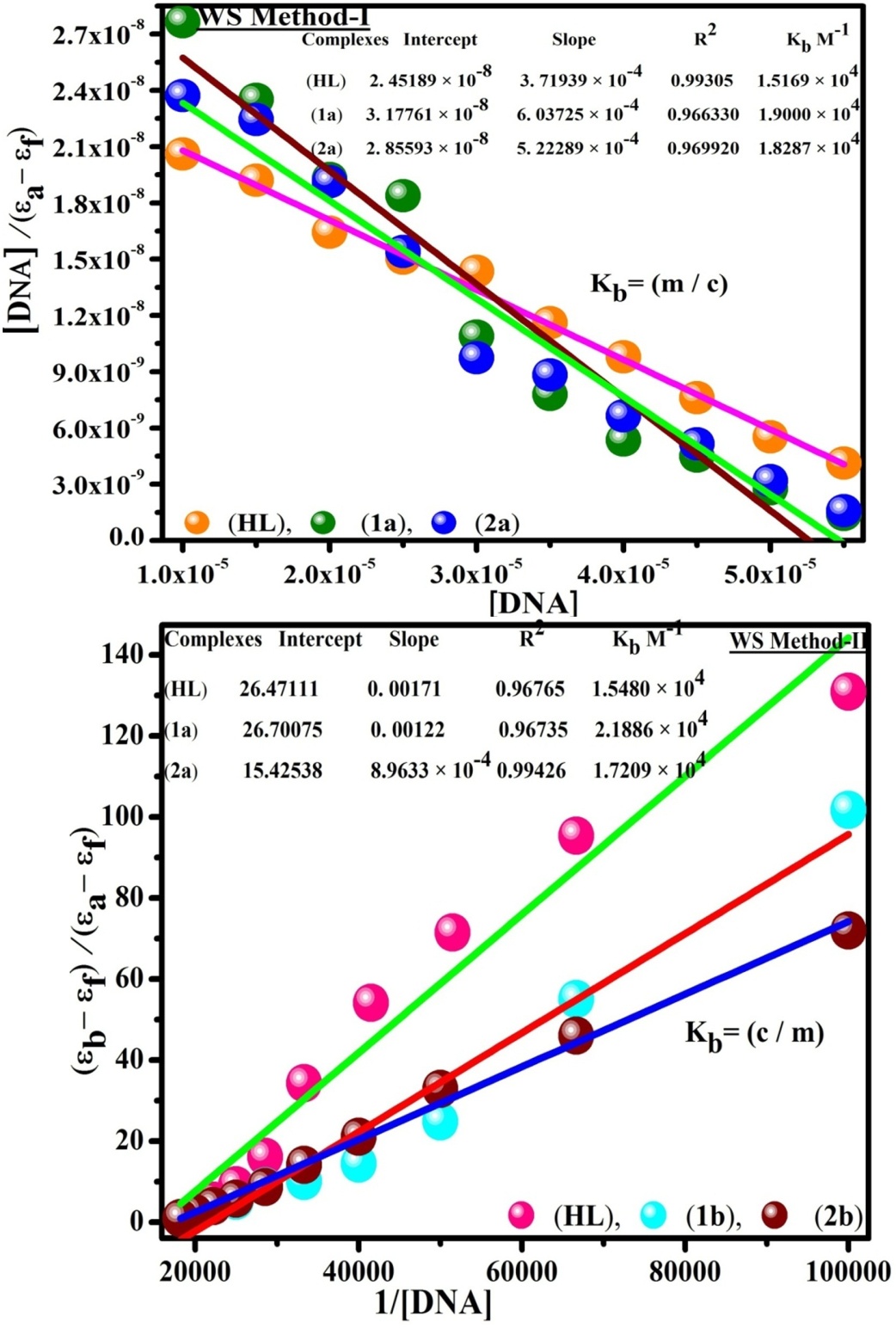**  **Fig.S17.** Linear plots of {[DNA] / ($ɛ$_a_ - $ɛ$_f_)} *vs* [DNA] and {($ɛ$_b_ - $ɛ$_f_)/ ($ɛ$_a_ - $ɛ$_f_)} *vs* [DNA] M^-1^ by Wolfe-Shimmer methods (I & II) for the estimation of the intrinsic DNA binding constants (K_b_) for ligand (**HL**) and mixed ligand complexes (**1a**–2**a**). |
| --- |

| **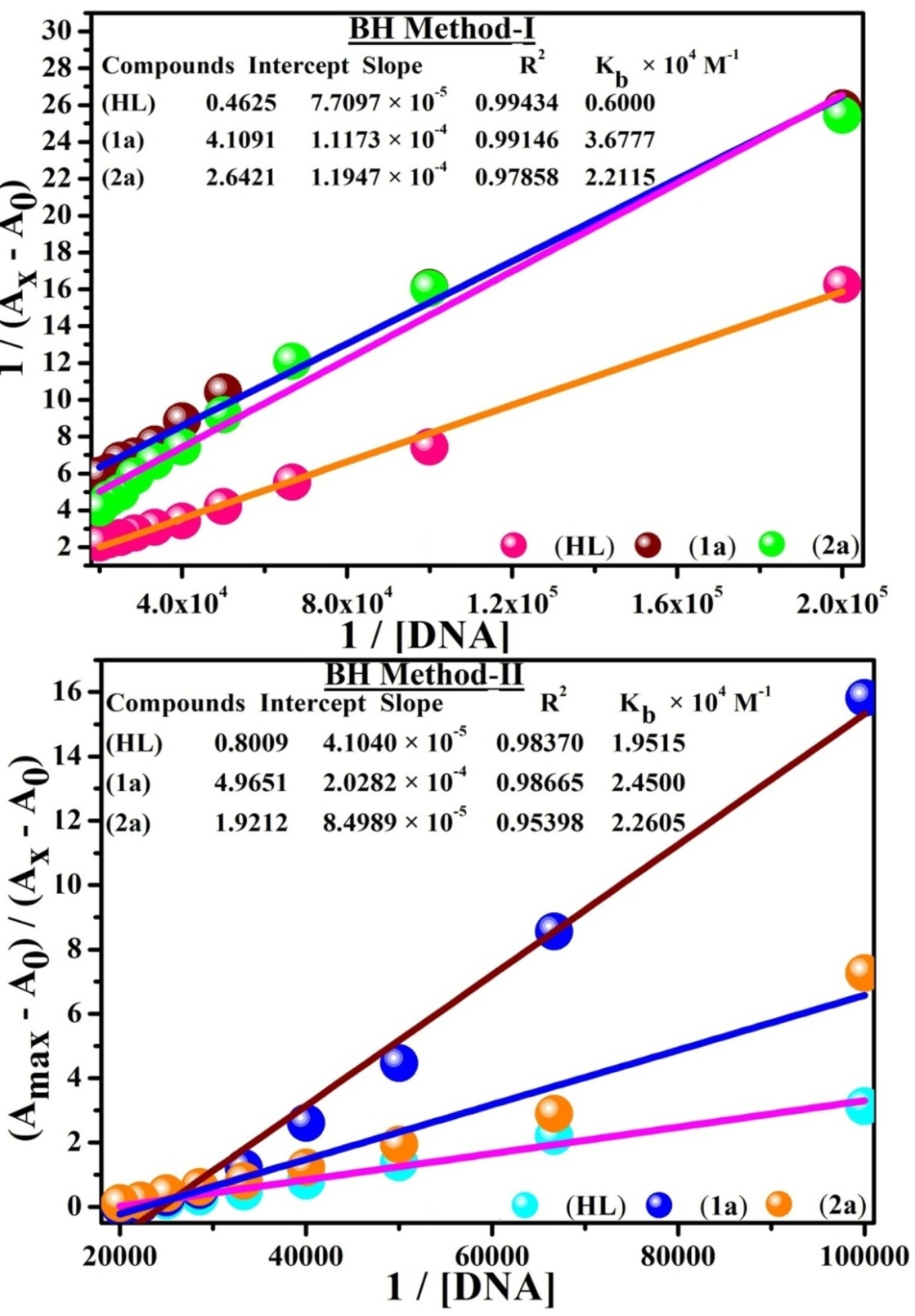**  **Fig.18.** Linear plots of [1 / (A_x_ - A_0_)] *vs* {1 / [DNA]} M^-1^ and [(A_max_ - A_0_) / (A_x_ - A_0_)] *vs* {1 / [DNA]} M^-1^ by Benesi-Hildebrand (I & II) methods for the estimation of the intrinsic DNA binding constants (K_b_) for ligand (**HL**) and complexes (**1a**–2**a**). |
| --- |

| **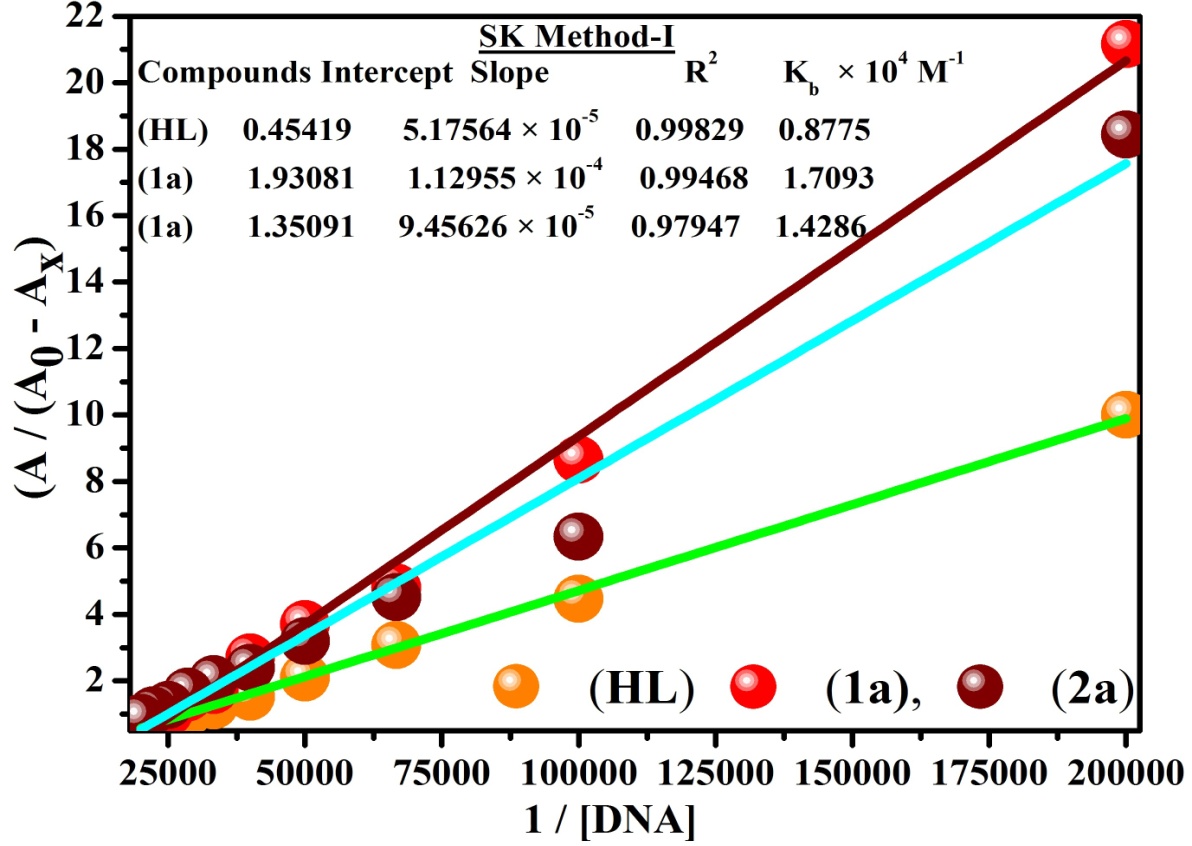**  **Fig.S19.** Linear plot of [(A / (A_0_ - A)] *vs* {1 / [DNA]} M^-1^ by Sakthi-Krause-I method for the estimation of the intrinsic DNA binding constants (K_b_) for ligand (**HL**) and complexes (**1a**–2**a**). |
| --- |

| **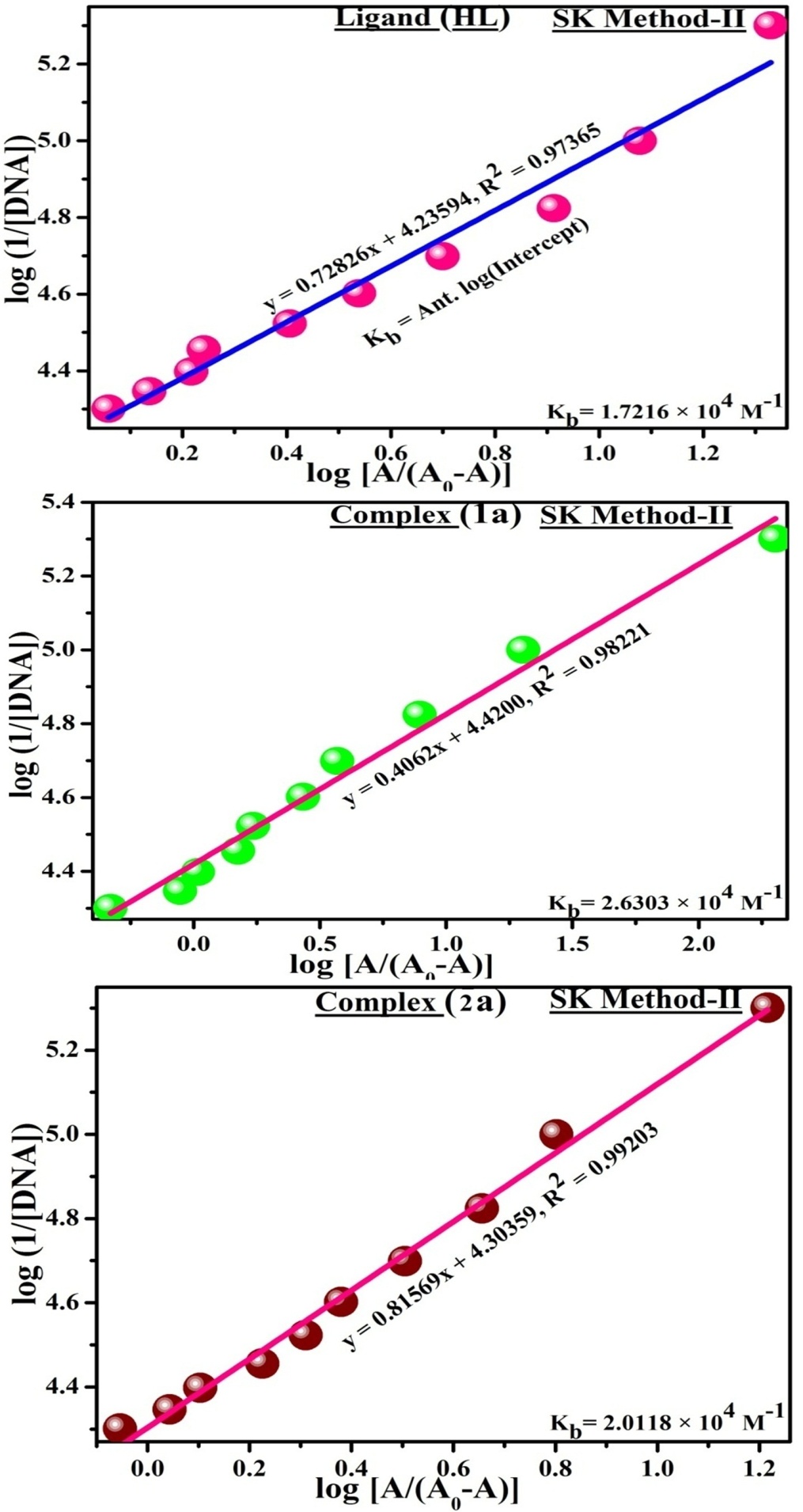**  **Fig.S20.** Linear plot of log{1 / [DNA]} M^-1^ *vs* log [(A / (A_0_ - A)] by Sakthi-Krause-II method for the determination of the intrinsic DNA binding constant (K_b_) for ligand (**HL**) and complexes (**1a**–2**a**). |
| --- |

| **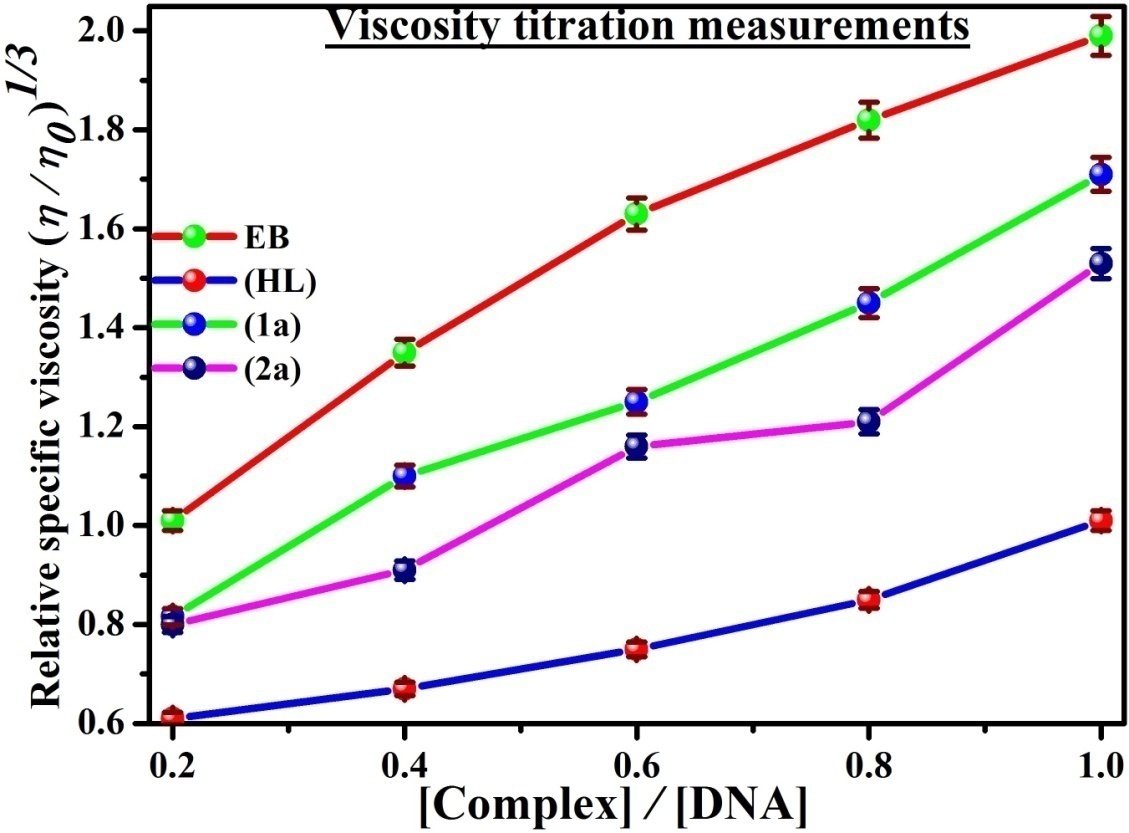**  **Fig.S21.** Relative specific viscosities of CT-DNA in the presence of increasing amounts of ligand (**HL**), mixed ligand complexes (**1a**–2**a**) and Ethidium bromide (**EB**) at 25 °C in 5mM Tris-HCl buffer pH = 7.2 and Error limit ± 2.0 %. |
| --- |

| 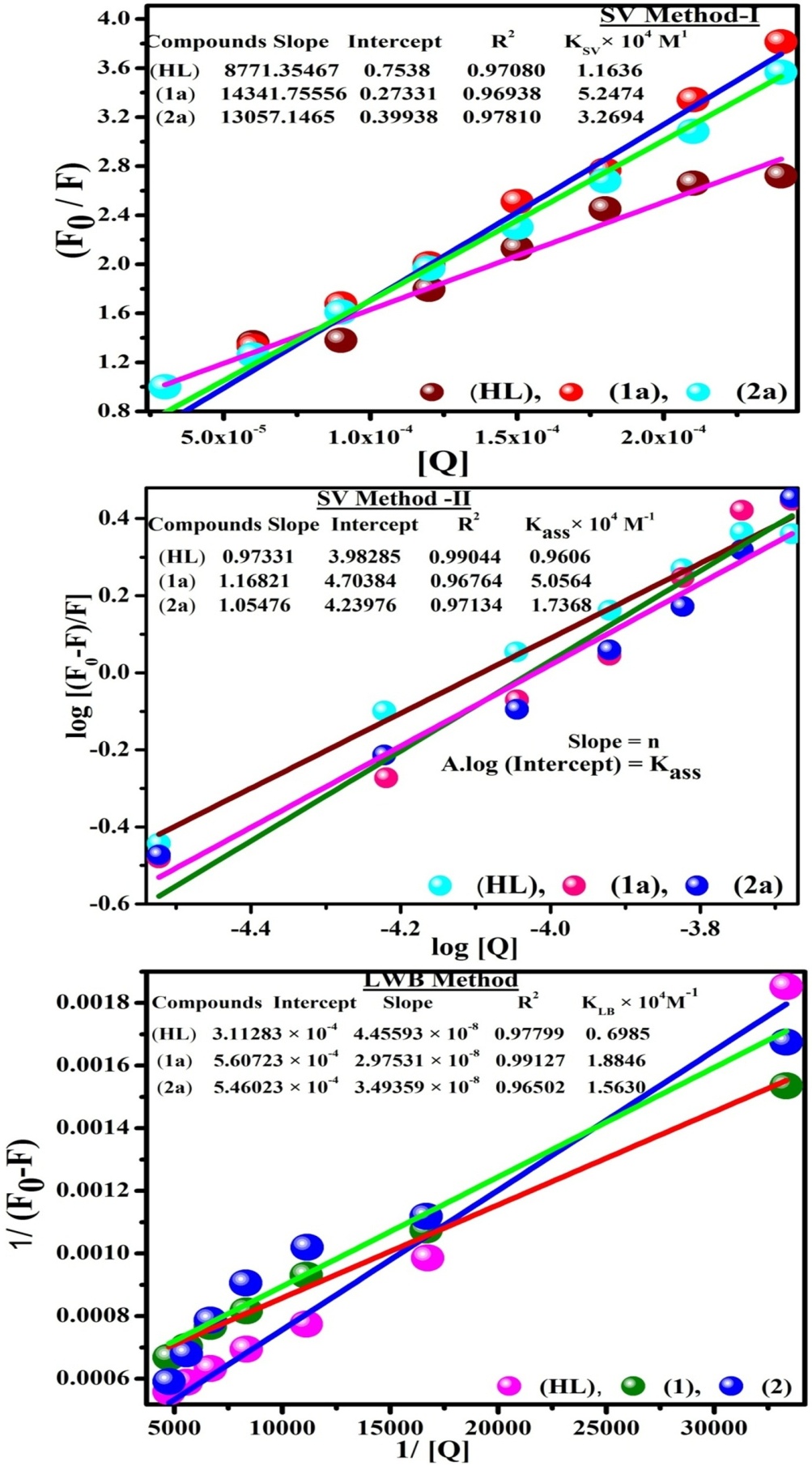  **Fig.S22.** Stern-Volmer linear plots of F_0_ / F *vs* [Q] (Method-I), log [(F_0_-F) / F] *vs* log [Q] (SV Method-II) and Lineweaver-Burk linear plot of 1/ (F_0_–F) *vs* 1/ [Q] for the quenching of fluorescence of ethidium bromide (EB)-DNA complex caused by ligand (**HL**) and mixed ligand complexes (**1a**–2**a**). |
| --- |

| 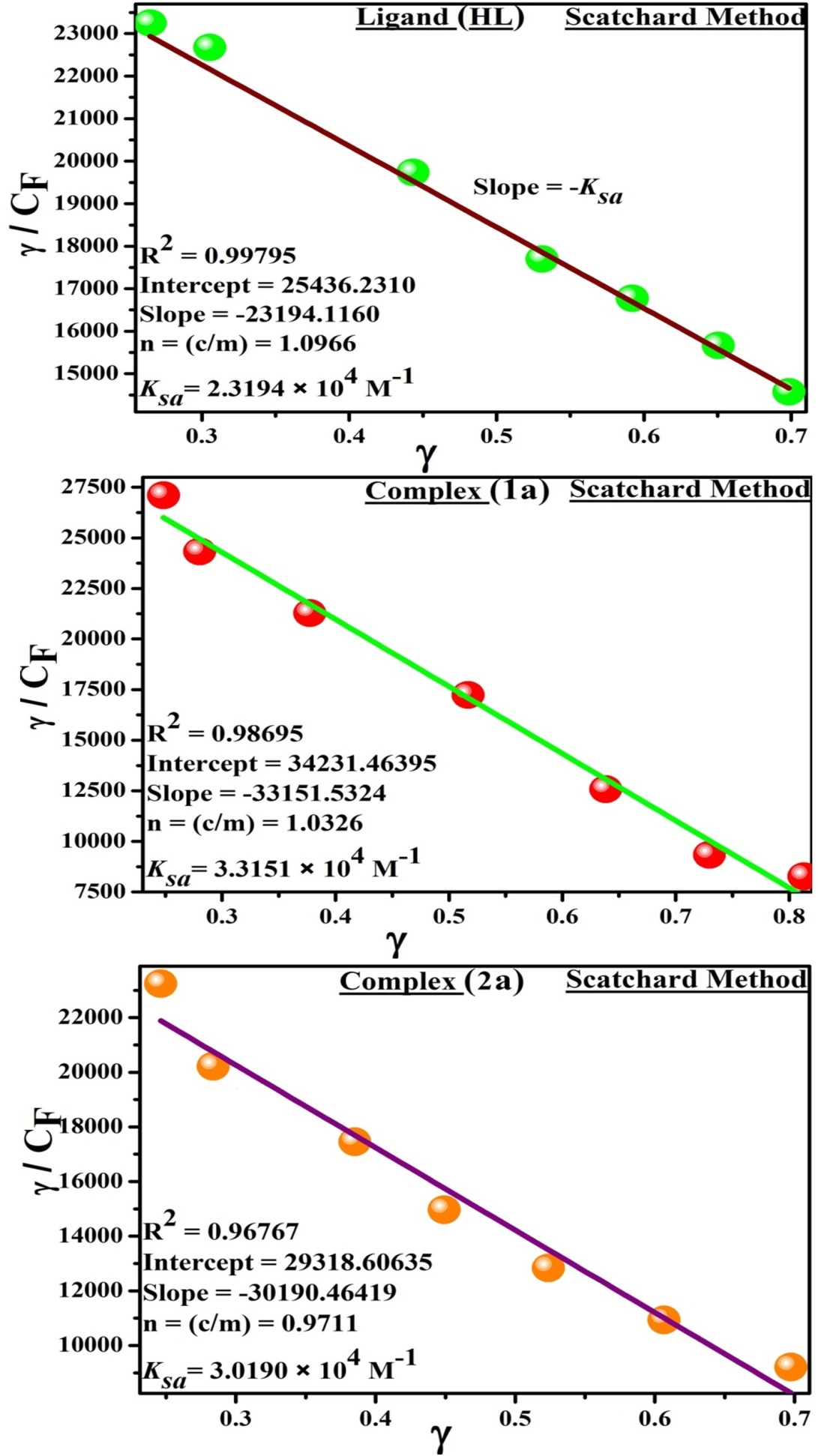  **Fig.S23.** Determining association binding constant and number of binding sites for ligand (**HL**) and complexes (**1a**–2**a**) – DNA by Scatchard linear plot of (γ / C_F_) *vs* γ. |
| --- |

| ***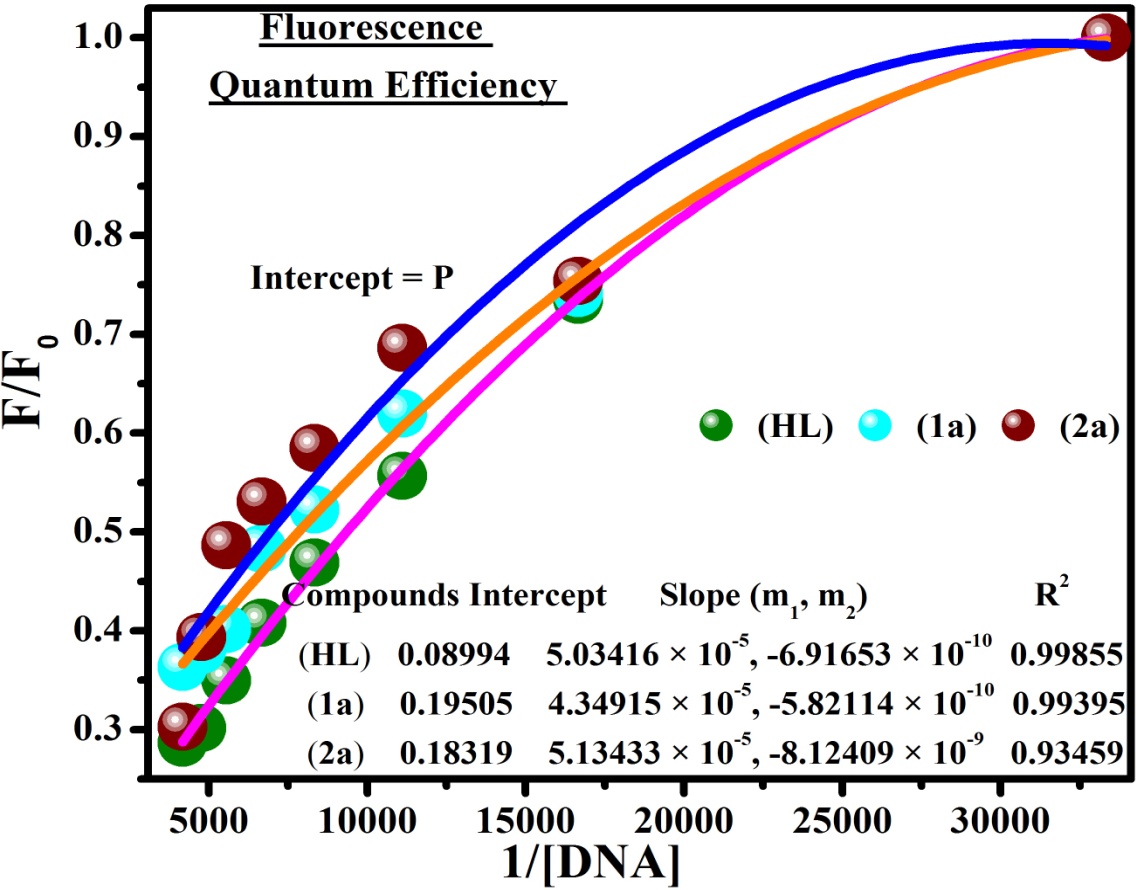***  **Fig.S24.** Determination of ratio of the fluorescence quantum efficiency (P) of DNA bound and free complex (P = ɸ_b_ /ɸ_f_ ) for ligand (**HL**) and mixed ligand complexes (**1a**–2**a**) from Stern–Volmer linear plots F/F0 *vs* 1/[DNA]. |
| --- |

| **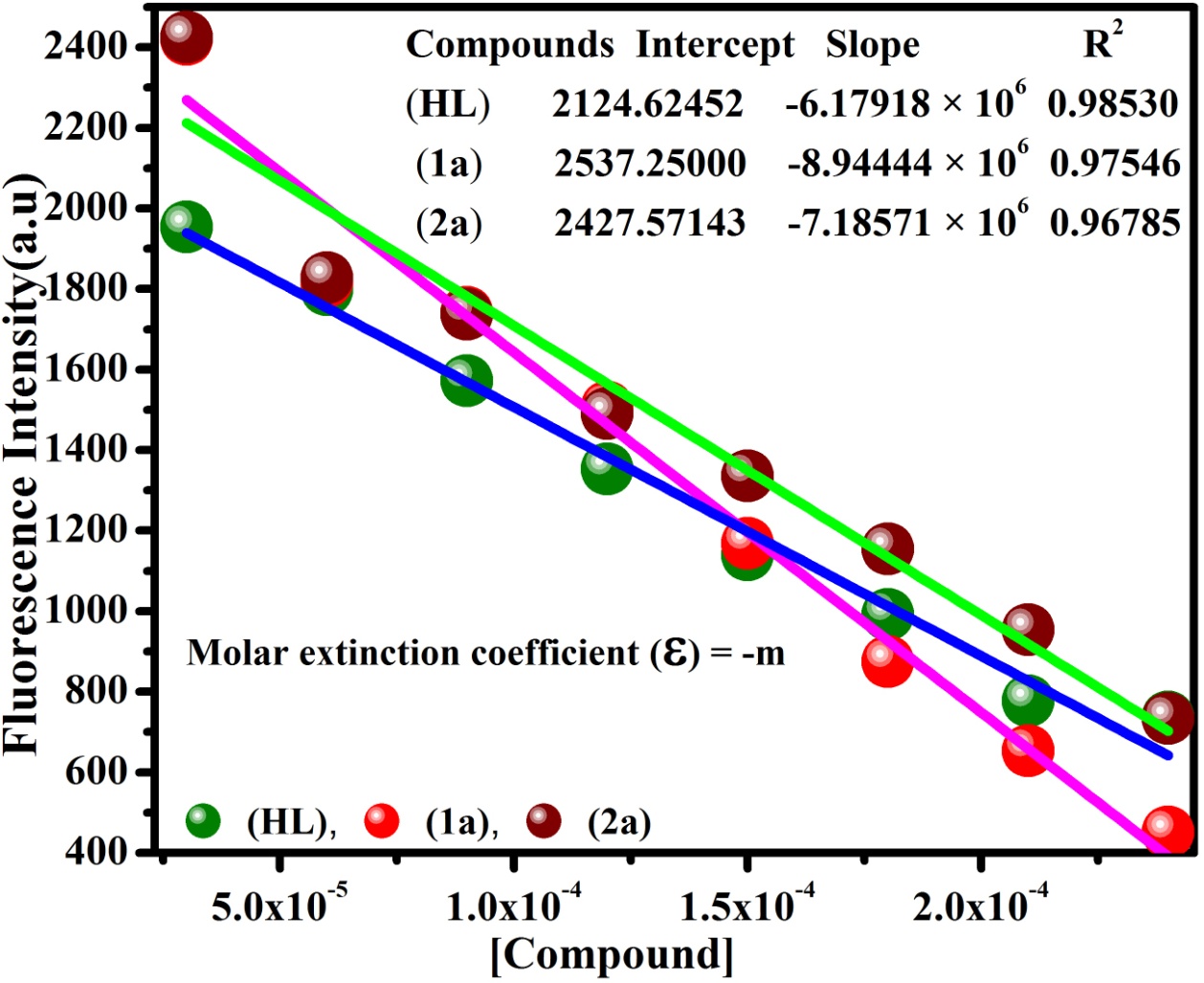**  **Fig.S25.** Determination of apparent binding constant (K*_app_*) from the linear plot of fluorescence intensity *vs* concentration of compounds. $K_{app} =K_{EB}\{\frac{\left[ \mathrm{EB} \right]}{\left[ \mathrm{compound} \right]}\}$; Where, K_EB_ = 10^7^ M^–1^ at the concentration of 50 µM EB. IC_50_ value of complex concentration was measured at a 50 % reduction of the fluorescence intensity of EB. |
| --- |

| 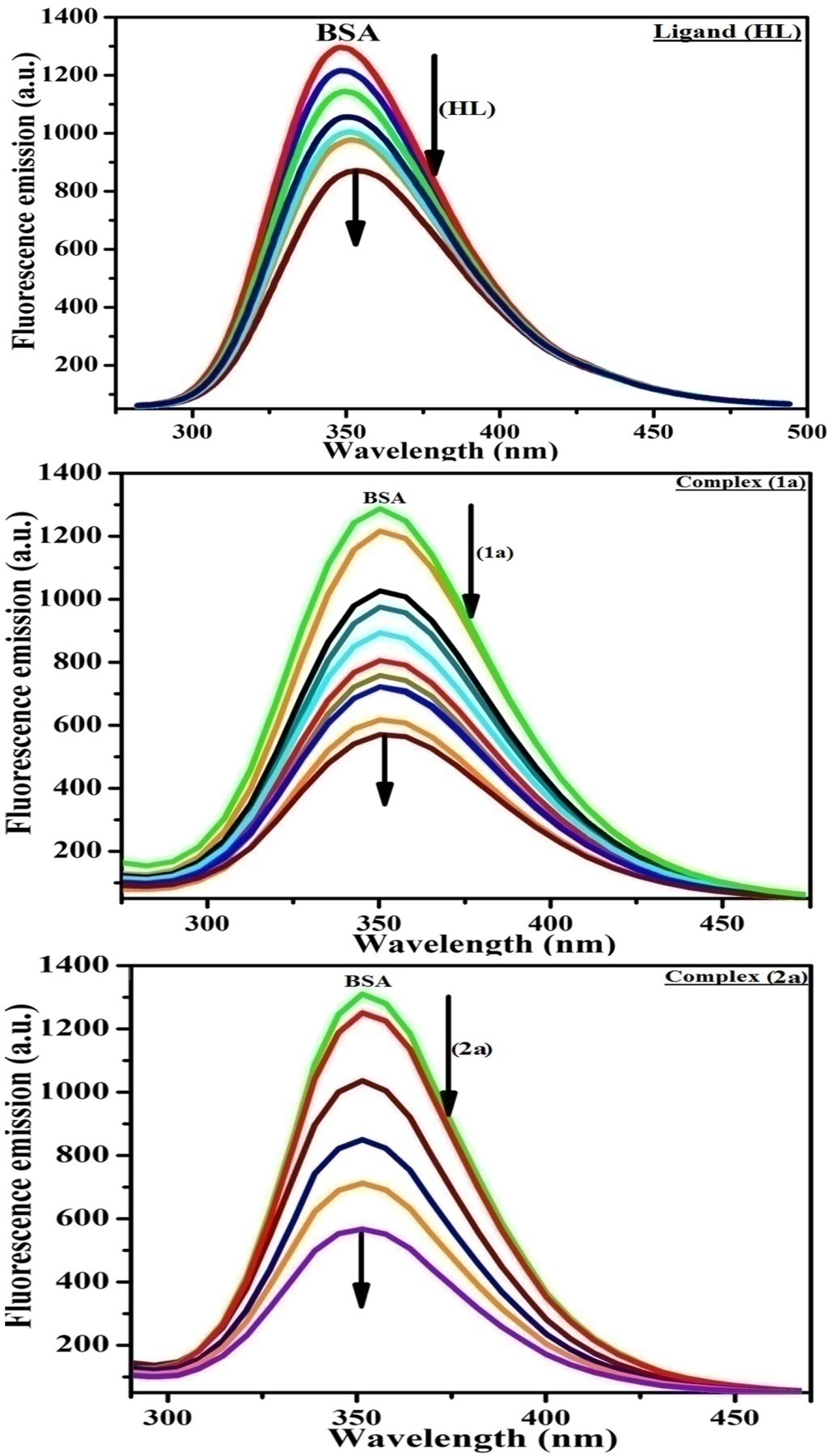  **Fig. S26.** Fluorescence quenching spectra of BSA in presence of various concentrations of ligand (**HL**) and mixed ligand complex (**1a**–2**a**). |
| --- |

| 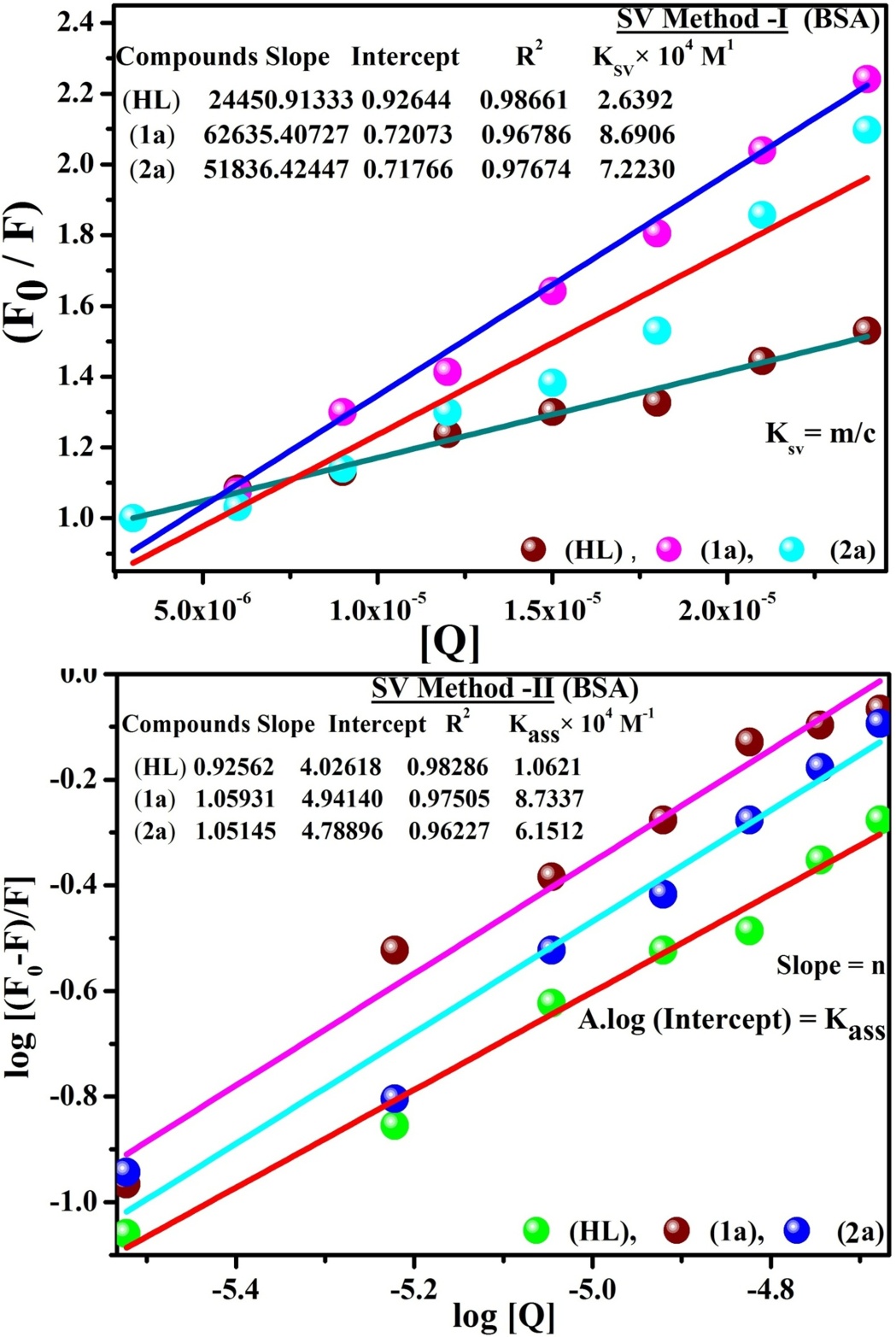  **Fig.S27.** Stern–Volmer linear plots of F_0_ / F *vs* [Q] (Method-I), log [(F_0_–F) / F] *vs* log [Q] (SV Method-II) for the fluorescence quenching of **BSA** by free ligand (**HL**) and mixed ligand complexes (**1a**–2**a**). |
| --- |

| 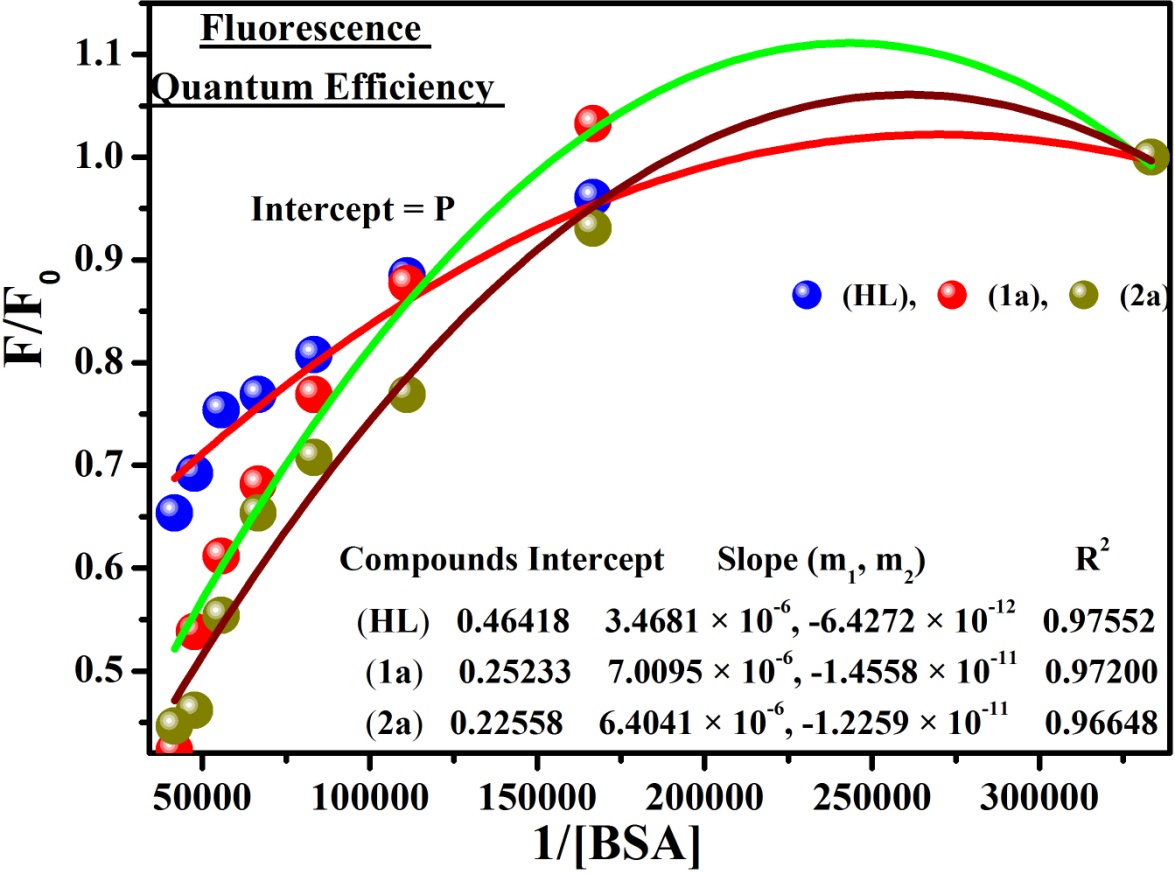  **Fig.S28.** Determination of ratio of the fluorescence quantum efficiency (P) of BSA bound and free complex (P = ɸ_b_ /ɸ_f_ ) for ligand (**HL**) and mixed ligand complexes (**1a**–2**a**) from Stern–Volmer linear plots F/F0 *vs* 1/[BSA]. |
| --- |

| 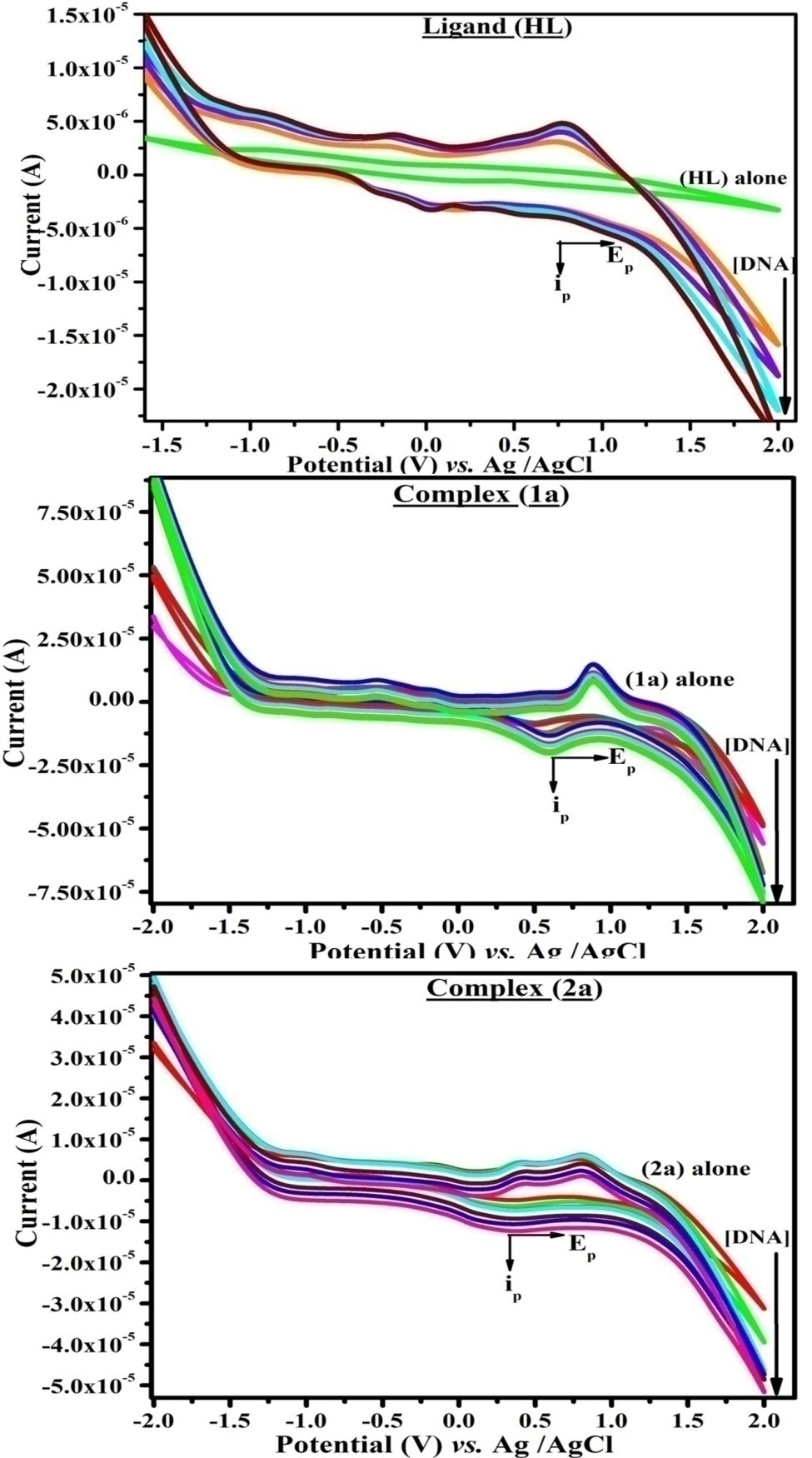  **Fig.S29.** Cyclic voltammograms of ligand (**HL**) and its complexes (**1a**–2**a**) in 0.05 M Tris-HCl buffer (Supporting electrolyte) pH = 7.2 at 25 °C in presence of increasing concentration of DNA (10–50 µM) and arrow indicates the changes in peak current and potentials at scan rate 100 mvs^-1^. |
| --- |

| **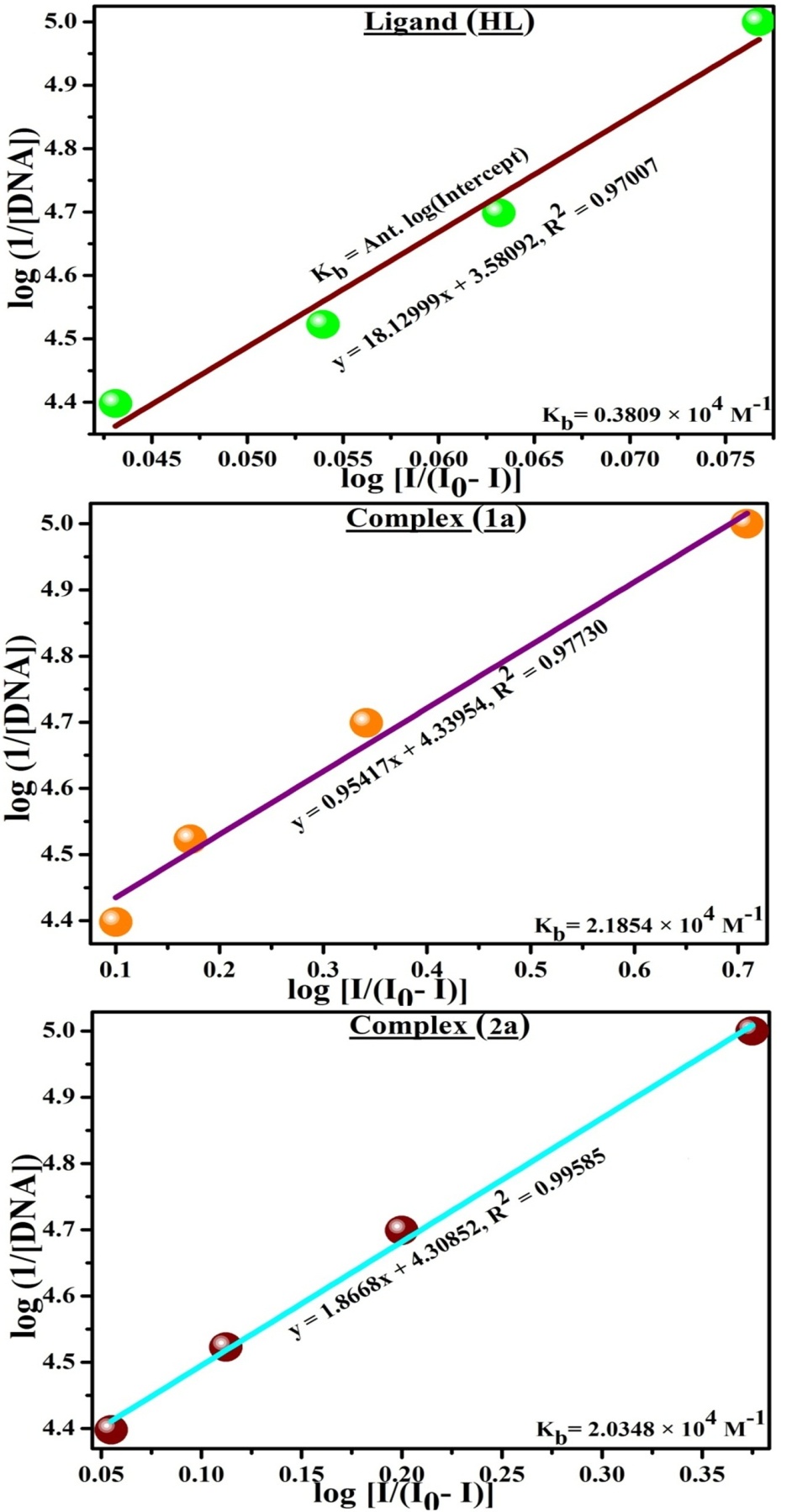**  **Fig.S30.** Plot of log (1/[DNA]) *vs* log (I/(I_0_−I)) to evaluate the binding constants for ligand (**HL**) and mixed ligand complexes (**1a**–2**a**) with DNA by Method-I. |
| --- |

| **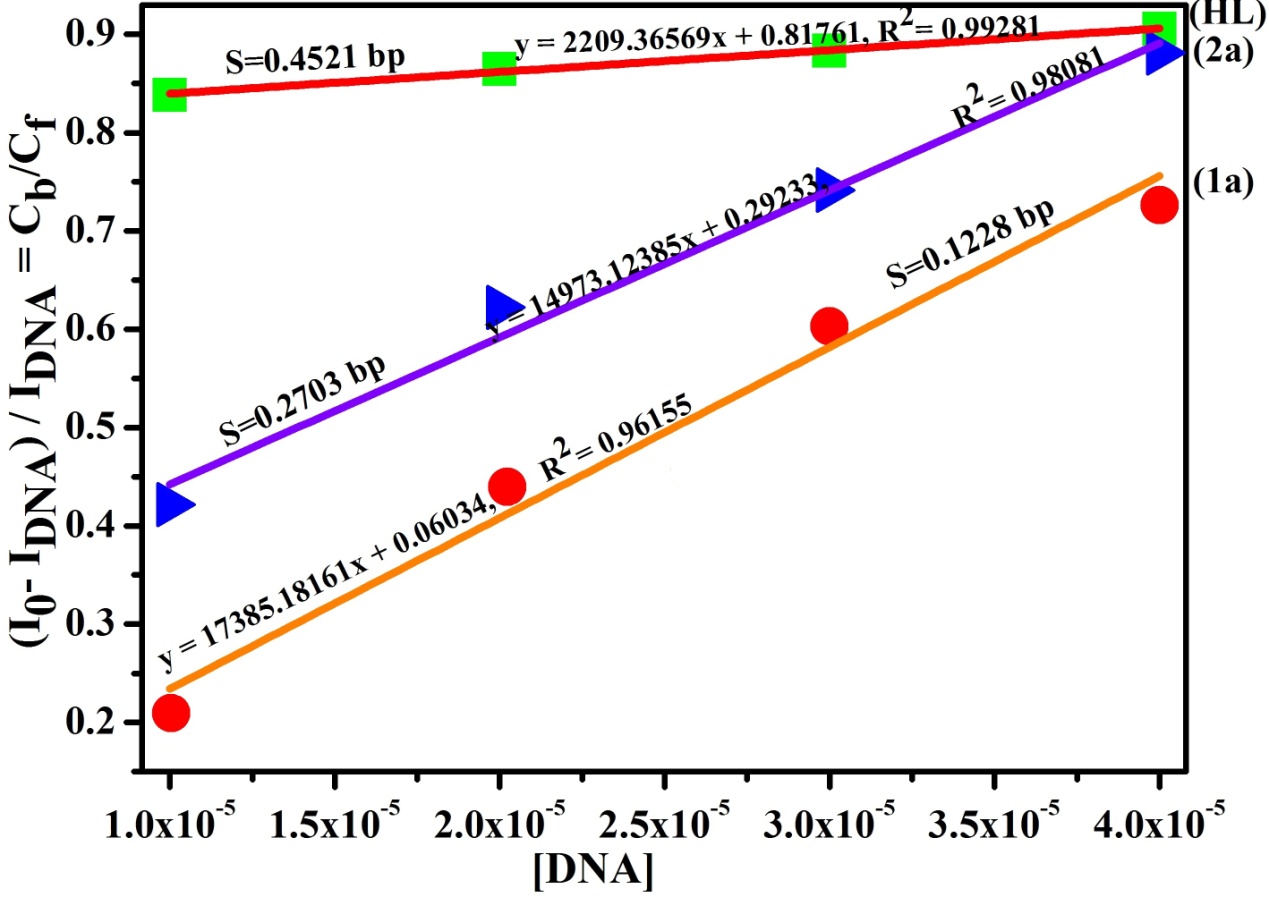**  **Fig.S31.** Plot of C_b_/C_f_ *vs* [DNA] for determination of binding site size (S) and binding constant for ligand (**HL**) and mixed ligand complexes (**1a**–2**a**) with DNA by Method-II. |
| --- |

| **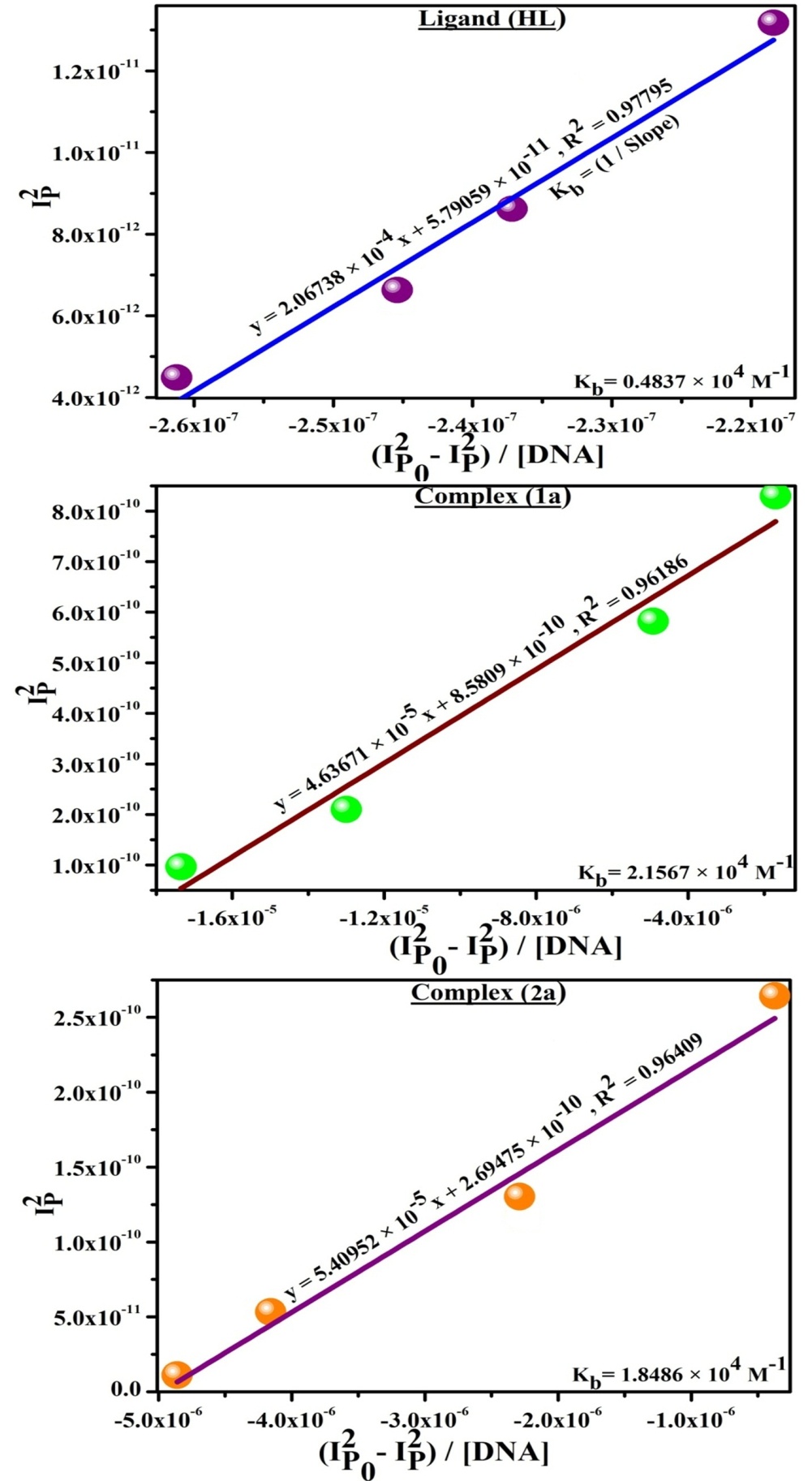**  **Fig.S32.** Plot of Ip^2^ *vs* (I_po_^2^ – I_p_^2^) / [DNA] to evaluate the binding constants for ligand (**HL**) and mixed ligand complexes (**1a**–2**a**) with DNA by Method-III. |
| --- |

| **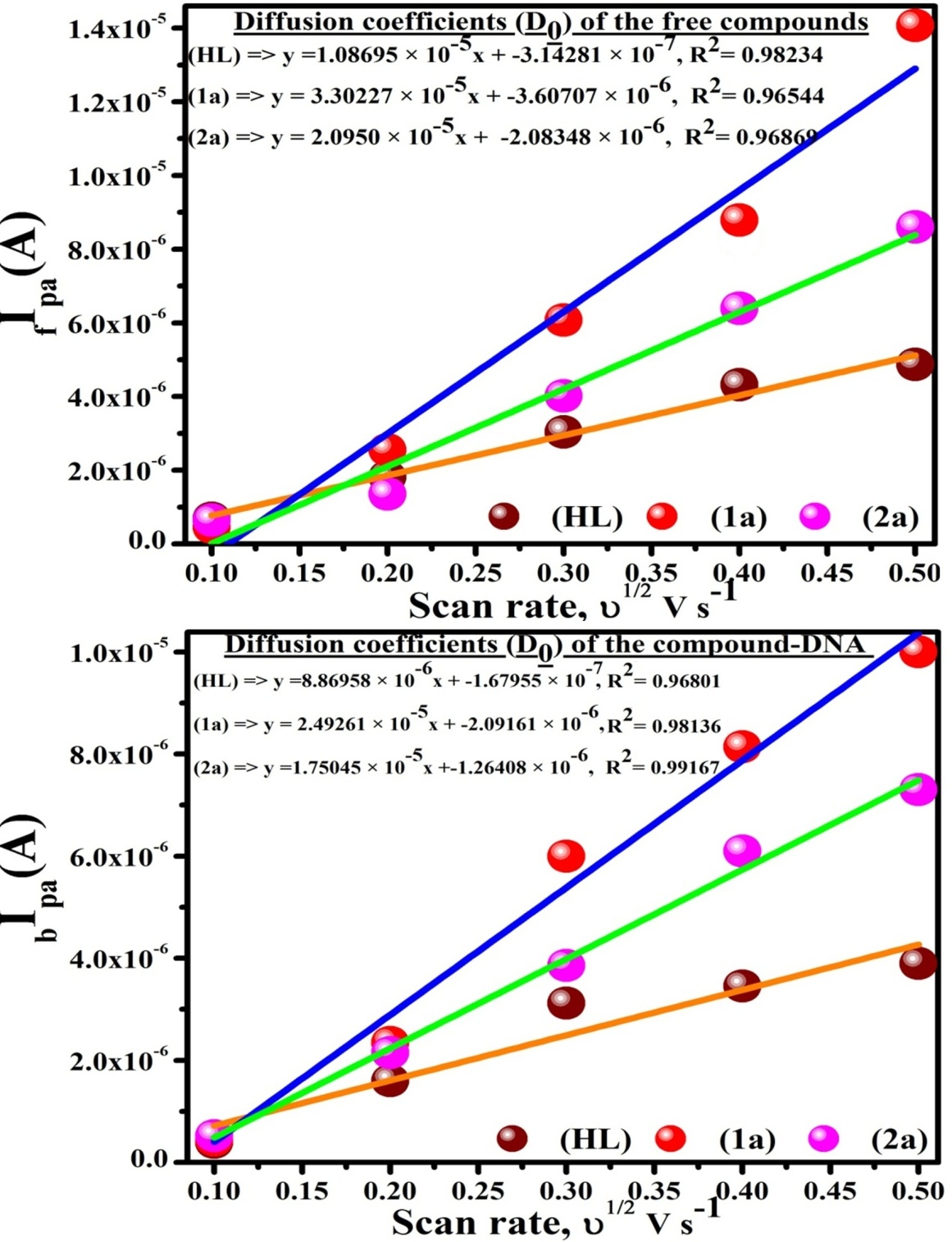**  **Fig.S33.** Plots of _f_Ipa *vs*. *v*^1/2^ and _b_Ipa *vs*. *v*^1/2^ for the determination of diffusion coefficients (D_0_) of the free compounds in the absence and presence of DNA at scan rates 0.01–0.3 V/s. |
| --- |

| **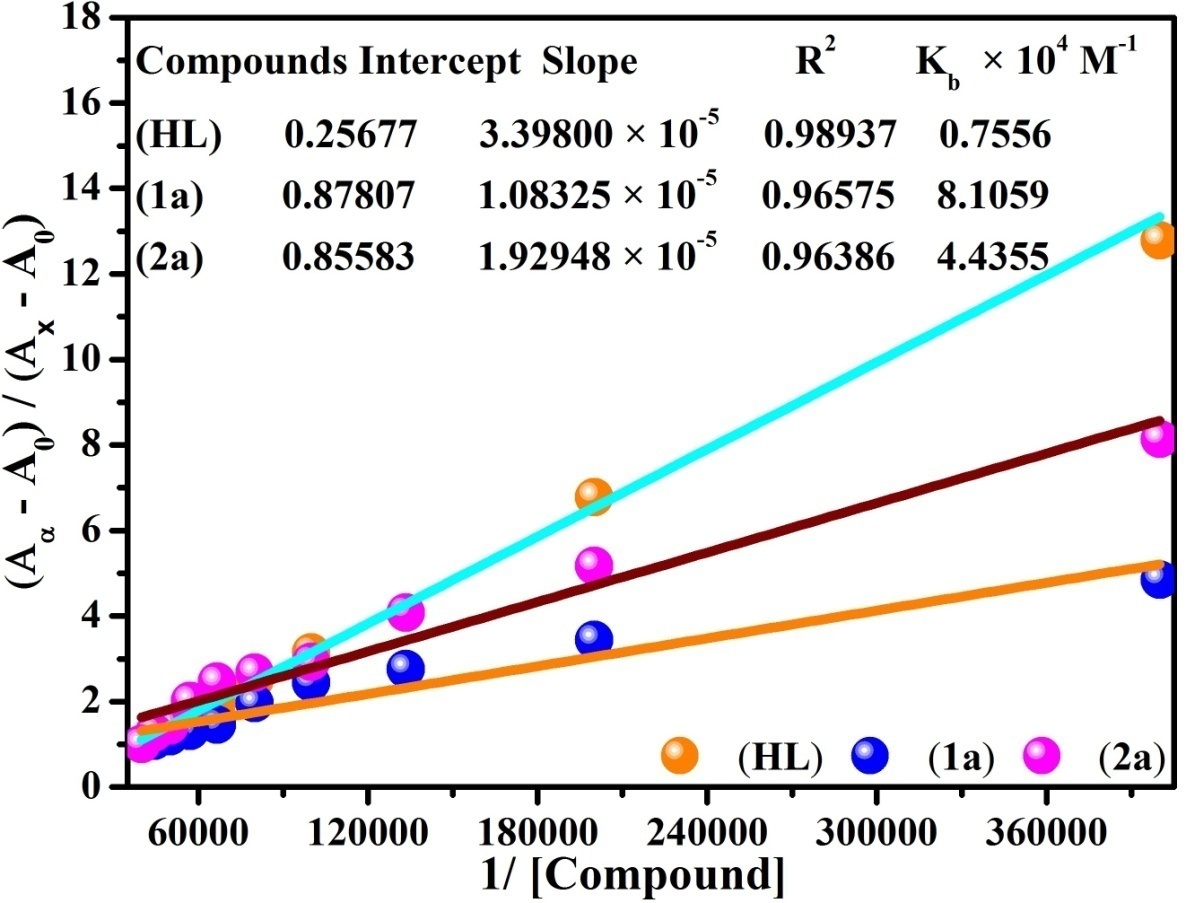**  **Fig.S34.** Benesi-Hildebrand linear plot [(A_∞_–A_0_) / (A_x_–A_0_)] *vs* 1 / [compound] determining binding constant for ligand (**HL**) and mixed ligand complexes (**1a**–2**a**) – BSA at 298 K. |
| --- |

| 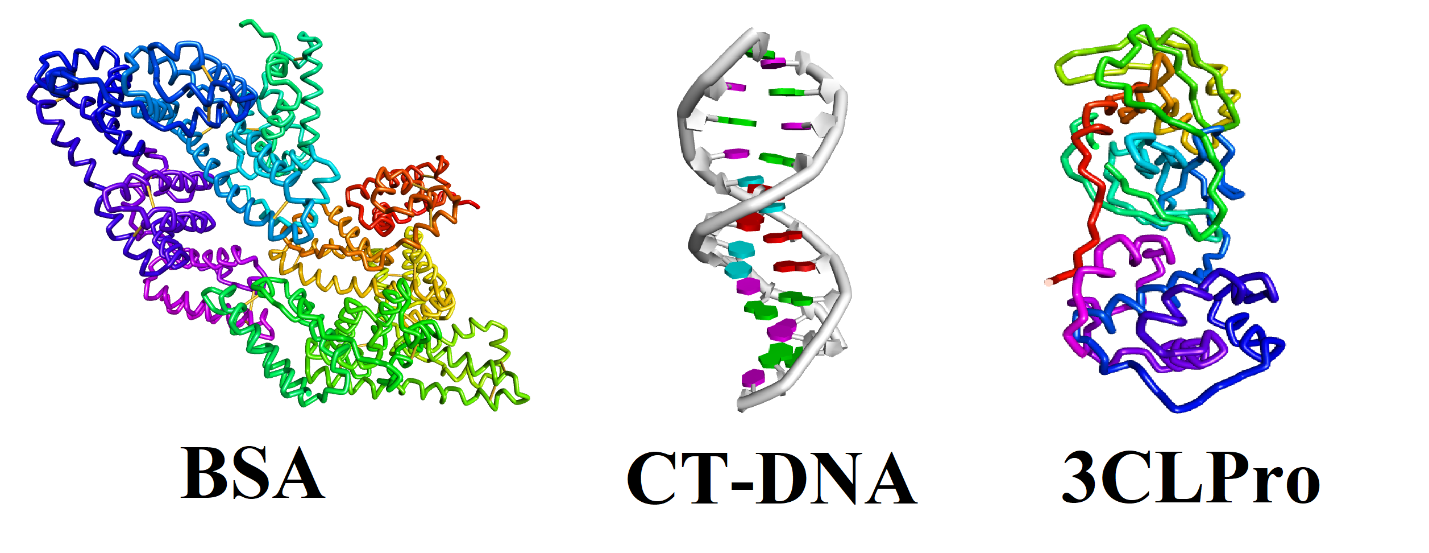  **Fig.S35.** 3D model of the host biomolecules used for docking calculations. . From left to right: BSA, CT-DNA and 3CLPro. |
| --- |

| 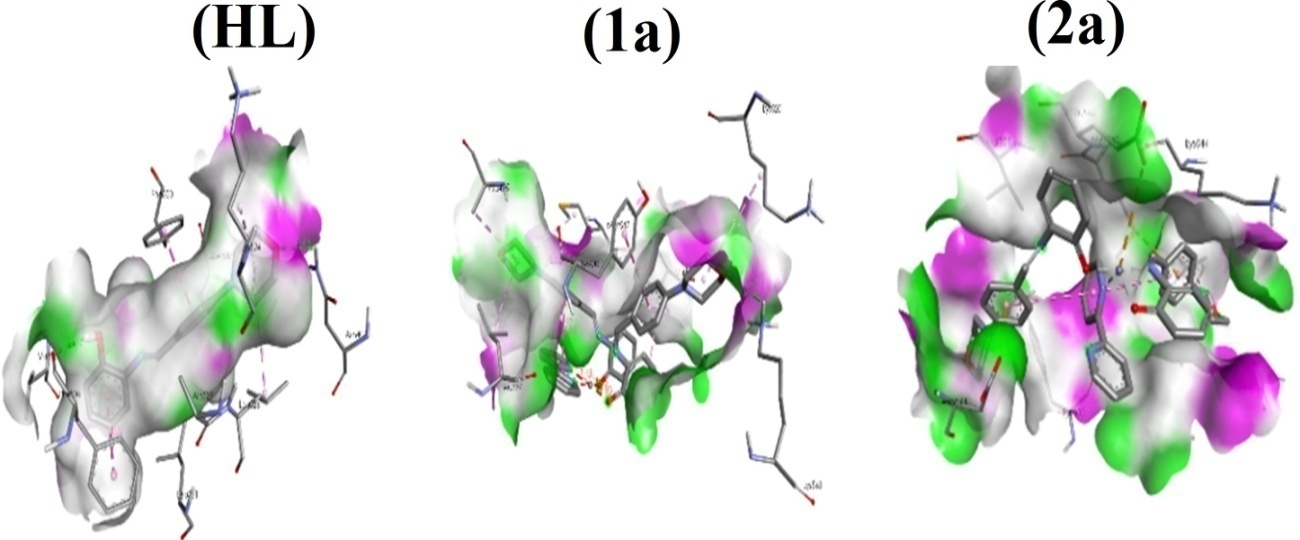  **Fig.S36.** Noncovalent interactions in the active site of the BSA protein. The pink and green colors denote H-bond acceptor and donor sites respectively. |
| --- |

| 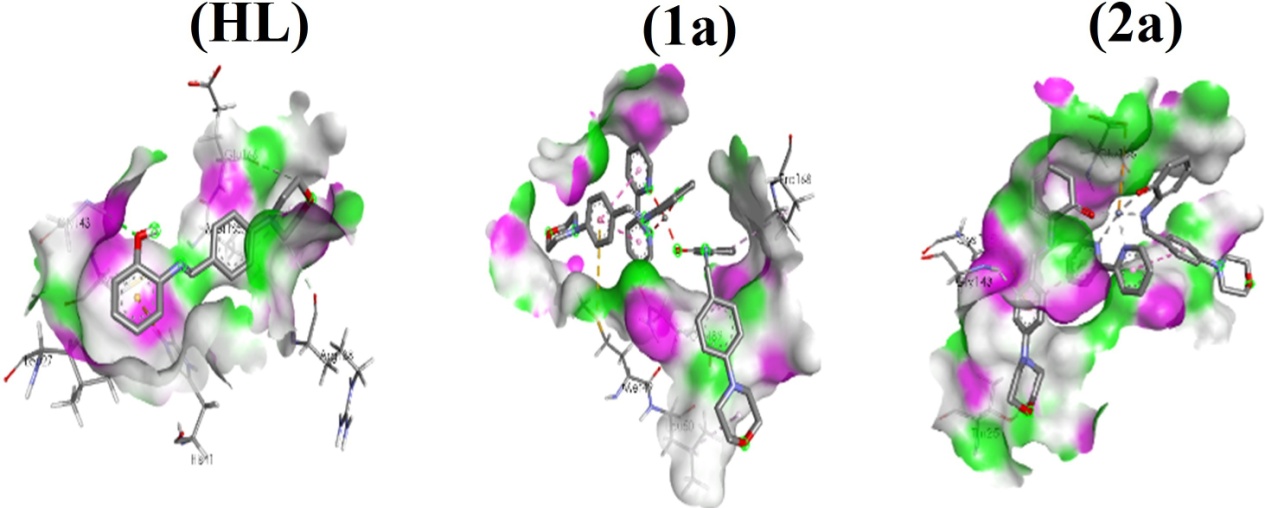  **Fig.S37.** Noncovalent interactions in the active site of the 3CLPro protein. The pink and green colors denote H-bond acceptor and donor sites respectively. |
| --- |

| **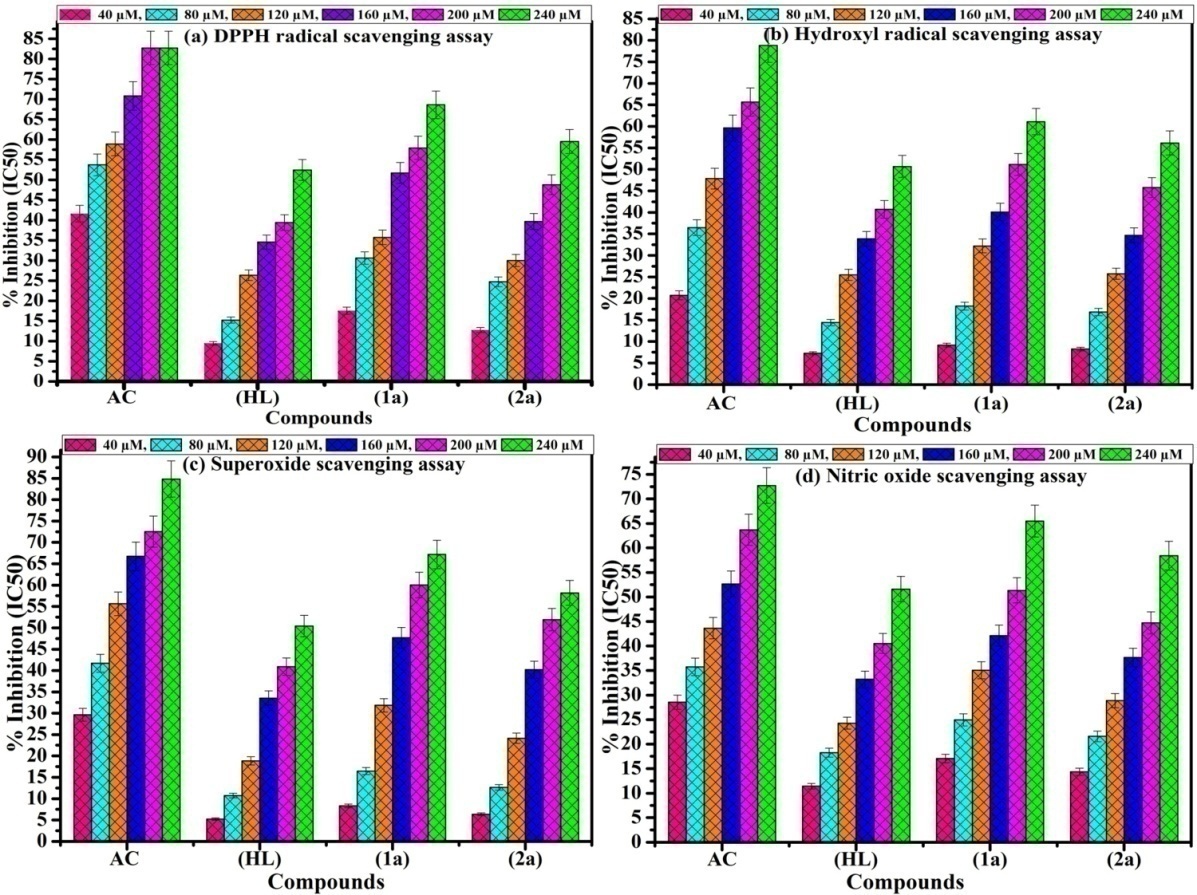**  **Fig. 5**  **Fig.S38.** % inhibition-IC_50_ of of (**a**) DPPH, (**b**) Hydroxyl, (**c**) Superoxide and (**d**) Nitric oxide scavenging assay for ligand (**HL**), complexes (**1a**–2**a**) and standard ascorbic acid (AC-control at various concentration (40, 80,120,160, 200, 240 µM). Error limits ± 2.5–5.0. |
| --- |

**Supplementary Tables**

| **Table S14** IC_50_ values of DPPH radical scavenging assay at 517 nm. | | | | | | |
| --- | --- | --- | --- | --- | --- | --- |
| **Compounds** | % Inhibition (IC_50_) | | | | | |
|  | **40 µM** | **80 µM** | **120 µM** | **160 µM** | **200 µM** | **240 µM** |
| Ascorbic acid | 41.63 | 53.74 | 58.95 | 70.86 | 82.75 | 85.65 |
| (**HL**) | 09.45 | 15.21 | 26.36 | 34.56 | 39.41 | 52.45 |
| (**1a**) | 17.53 | 30.62 | 35.74 | 51.72 | 57.95 | 68.64 |
| (**2a**) | 12.75 | 24.73 | 30.02 | 39.68 | 48.79 | 59.55 |
| $\mathrm{Scavanging}\left( \% \right)=\left[ \frac{(A_{0} -A_{S})}{A_{0}} \right]\times100$ (**43**); Where, A_0_ → absorbance of the control or (DPPH alone in ethanol) and A_S_ → absorbance of the sample or (Mixture of DPPH and compounds in ethanol). Error limits ± 2.5 - 5.0 %. | | | | | | |

| **Table S15** IC_50_ values of Hydroxyl radical scavenging assay at 230 nm. | | | | | | |
| --- | --- | --- | --- | --- | --- | --- |
| **Compounds** | % Inhibition (IC_50_) | | | | | |
|  | **40 µM** | **80 µM** | **120 µM** | **160 µM** | **200 µM** | **240 µM** |
| Ascorbic acid | 20.73 | 36.46 | 47.87 | 59.65 | 65.64 | 78.83 |
| (**HL**) | 07.26 | 14.43 | 25.52 | 33.86 | 40.73 | 50.68 |
| (**1a**) | 09.16 | 18.23 | 32.22 | 40.12 | 51.14 | 61.10 |
| (**2a**) | 08.25 | 16.86 | 25.78 | 34.66 | 45.78 | 56.14 |

| **Table S16** IC_50_ values of Superoxide scavenging assay at 590 nm. | | | | | | |
| --- | --- | --- | --- | --- | --- | --- |
| **Compounds** | % Inhibition (IC_50_) | | | | | |
|  | **40 µM** | **80 µM** | **120 µM** | **160 µM** | **200 µM** | **240 µM** |
| Ascorbic acid | 29.64 | 41.72 | 55.63 | 66.75 | 72.55 | 84.85 |
| (**HL**) | 05.23 | 10.74 | 18.85 | 33.55 | 40.91 | 50.42 |
| (**1a**) | 08.32 | 16.45 | 31.86 | 47.69 | 60.03 | 67.17 |
| (**2a**) | 06.32 | 12.67 | 24.14 | 40.20 | 51.92 | 58.18 |

| **Table S17** IC_50_ values of Nitric oxide scavenging assay at 546 nm. | | | | | | |
| --- | --- | --- | --- | --- | --- | --- |
| **Compounds** | % Inhibition (IC_50_) | | | | | |
|  | **40 µM** | **80 µM** | **120 µM** | **160 µM** | **200 µM** | **240 µM** |
| Ascorbic acid | 28.55 | 35.75 | 43.65 | 52.68 | 63.72 | 72.73 |
| (**HL**) | 11.42 | 18.26 | 24.28 | 33.21 | 40.53 | 51.62 |
| (**1a**) | 17.07 | 24.95 | 35.06 | 42.14 | 51.35 | 65.47 |
| (**2a**) | 14.37 | 21.59 | 28.87 | 37.69 | 44.73 | 58.43 |
